# Microbial β-glucosidases from cow rumen metagenome enhance the saccharification of lignocellulose in combination with commercial cellulase cocktail

**DOI:** 10.1186/1754-6834-5-73

**Published:** 2012-09-21

**Authors:** Mercedes V Del Pozo, Lucía Fernández-Arrojo, Jorge Gil-Martínez, Alejandro Montesinos, Tatyana N Chernikova, Taras Y Nechitaylo, Agnes Waliszek, Marta Tortajada, Antonia Rojas, Sharon A Huws, Olga V Golyshina, Charles J Newbold, Julio Polaina, Manuel Ferrer, Peter N Golyshin

**Affiliations:** 1 CSIC, Institute of Catalysis, 28049, Madrid, Spain; 2 Abengoa Bioenergía Nuevas Tecnologías S.A., 41012, Sevilla, Spain; 3 Biopolis S.L, 48980, Paterna, Valencia, Spain; 4 School of Biological Sciences, Bangor University, LL57 2UW, Gwynedd, UK; 5 Insect Symbiosis Research Group, Max Planck Institute for Chemical Ecology, 07745, Jena, Germany; 6 Environmental Microbiology Department, HZI-Helmholtz Centre for Infection Research, D-38124, Braunschweig, Germany; 7 Institute of Biological, Environmental and Rural Sciences, Aberystwyth University, Aberystwyth, SY23 3DA, UK; 8 Centre for Integrated Research in the Rural Environment, Aberystwyth University-Bangor University Partnership (CIRRE), Aberystwyth, SY23 3DA, UK; 9 CSIC, Instituto de Agroquímica y Tecnología de Alimentos, 46980, Paterna, Valencia, Spain

**Keywords:** Beta-glucosidases, Bio-ethanol, Glycosyl hydrolase, Lignocellulose, Metagenome, Rumen

## Abstract

**Background:**

A complete saccharification of plant polymers is the critical step in the efficient production of bio-alcohols. Beta-glucosidases acting in the degradation of intermediate gluco-oligosaccharides produced by cellulases limit the yield of the final product.

**Results:**

In the present work, we have identified and then successfully cloned, expressed, purified and characterised 4 highly active beta-glucosidases from fibre-adherent microbial community from the cow rumen. The enzymes were most active at temperatures 45–55°C and pH 4.0-7.0 and exhibited high affinity and activity towards synthetic substrates such as *p*-nitrophenyl-beta-D-glucopyranoside (*p*NPbetaG) and *p*NP-beta-cellobiose, as well as to natural cello-oligosaccharides ranging from cellobiose to cellopentaose. The apparent capability of the most active beta-glucosidase, herein named LAB25g2, was tested for its ability to improve, at low dosage (31.25 units g^-1^ dry biomass, using *p*NPbetaG as substrate), the hydrolysis of pre-treated corn stover (dry matter content of 20%; 350 g glucan kg^-1^ dry biomass) in combination with a beta-glucosidase-deficient commercial *Trichoderma reseei* cellulase cocktail (5 units g^-1^ dry biomass in the basis of *p*NPbetaG). LAB25g2 increased the final hydrolysis yield by a factor of 20% (44.5 ± 1.7% vs. 34.5 ± 1.5% in control conditions) after 96–120 h as compared to control reactions in its absence or in the presence of other commercial beta-glucosidase preparations. The high stability (half-life higher than 5 days at 50°C and pH 5.2) and 2–38000 fold higher (as compared with reported beta-glucosidases) activity towards cello-oligosaccharides may account for its performance in supplementation assays.

**Conclusions:**

The results suggest that beta-glucosidases from yet uncultured bacteria from animal digestomes may be of a potential interest for biotechnological processes related to the effective bio-ethanol production in combination with low dosage of commercial cellulases.

## Background

Cellulose and hemicellulose are the major components of plant cell walls and the most abundant biopolymeric materials on our Planet [1-3]. The natural breakdown of plant matter performed by hemi-cellulases [4,5], has been exploited by biotechnologists to produce bio-fuels, e.g. bio-ethanol [5-7]. Although existing technological developments for biomass pre-treatment facilitate the saccharification process [8], there are some technological barriers hindering an efficient scale-up of the technology. First, the high costs and the low specific activity of the enzymatic cocktails available and actually used; second, the limited number of companies producing the enzymes for the saccharification of plant polymers. These factors weaken the worldwide production of bio-ethanol from plant matter and increase the costs of bio-ethanol production [2]. The high costs of enzymes for biomass decomposition have been identified as a major impediment to the economic conversion of lignocellulosic feedstocks into the bio-ethanol [9,10]. In that context, in 2004 the National Laboratory of Renewable Energy of USA, in association with the two major producers of industrial enzymes (Genencor International and Novozymes A/S), have achieved a drastic reduction in the cellulase price, namely by a factor of 20–30. The new technology utilized an enzyme cocktail with three major enzymes needed to hydrolyse cellulose (endoglucanases, exoglucanases and β-glucosidases). The two first enzymes, working in a synergetic manner, perform the hydrolysis of cellulose chains to cellobiose, destroying the crystalline structure, while the third enzyme transforms the cellobiose to glucose [5]. Therefore, it is crucial to further reduce the enzyme costs in order to develop and maintain sustainable industrial installations that would use cellulose for the bio-ethanol production as common as petroleum in refineries [11]. For example, in industrial settings that use the plant starch-rich materials instead of cellulose as the feedstock, the cost of amylase needed for the degradation of starch is lower than 0,002-0,004 € per litre; by contrast, the most affordable enzyme cocktails that use cellulose do cost 10 times higher [12]. Furthermore, it is worth mentioning that to hydrolyse 1 g of pre-treated plant biomass *ca.* 25 mg enzyme cocktail is needed and that the actual market objective is to achieve a decrease in the enzyme cost from 250 € to 100 € per m^3^ of produced bio-ethanol.

To deal with the objective of reducing the enzyme costs, two different approaches are complementarily used, namely, the production of new or improved transgenic yeasts/fungi capable to secrete wild-type or engineered enzymes, and the identification of new or improved enzymes in combination with low-dosage of commercial cellulases. Since the currently used commercial cellulases generally exhibit low β-glucosidase activity (despite recent developments), it has been suggested that the β-glucosidase of the glycosyl hydrolase family 3 (GHF3; β-glucosidase/xylosidase) acting in the degradation of cellulosic glucans can be used for the saccharification of intermediate gluco-oligosaccharides produced by endoglucanases [13]. Certainly, the addition of β-glucosidases, endoxylanases and α-arabinofuranosidases for the hydrolysis of steam-exploded wheat straw has been found to improve the hydrolysis from 10% to 29.5% as compared to the non-supplemented reactions [14-17].

Bacterial GHF3 contain about 2,081 known enzymes (http://www.cazy.org; [18]). Microbial communities operating in gastrointestinal tracts of herbivorous animals, termites and earthworms, are continuously exposed to a strong diet-driven selective pressure by chemically diverse and complex plant polymeric compounds, and thus represent a rich hotspot for diverse β-glucosidases. The latter can be identified and characterized using functional metagenomics approach, a promising technique to assess the genetic content of complex microbial communities without culturing individual members of microbial communities. Nevertheless, despite recent discoveries and availability of novel β-glucosidases from uncultured microbes (for examples see ref. [19-24]), their potential for the enzymatic conversion of lignocellulose into fermentable sugars has not been assessed as yet.

Here, four β-glucosidases from cow rumen microbial community were identified and characterized, and their potential utilization for supplementation of enzyme cocktails for hydrolysis of plant biomass, namely pre-treated corn stover, was tested. Our findings are pointing at the importance of rumen digestomes as rich resources for novel lignocellulases potentially useful for the effective enzymatic conversion of plant biomass into fermentable sugars.

## Results

### Library screening and general characteristics

The rumen libraries (approx. 17,000 clones harbouring ca. 600 Mbp of metagenomic DNA) were screened for the ability to hydrolyse *p*-nitrophenyl-β-D-glucopyranoside (*p*NPβG) and *p*-nitrophenyl-β-D-cellobioside (*p*NPβC). We identified three positives (designated SRF2, LAB20 and LAB25) as being highly active against both substrates. The fosmids with inserts SRF2 (34,226 bp; G + C content of 50.04%), LAB20 (38,523 bp; G + C 61.78%) and LAB25 (6,892 bp; G + C 52.16%) were fully sequenced [NCBI acc. nr: JX163905, JX163906 and JX163904, in the same order]. In total, 68 predicted coding sequences (CDS) were identified, which were named according to the fosmid ID and the number of the CDS in the genomic fragment sequenced (provided in Additional file 1: Table S1). Eight of them corresponded to putative glycosyl hydrolase (GH)-like polypeptides with fosmids SRF2, LAB20 and LAB25 containing 4, 3 and 1, in the same order. Those included a GHF31 α-glucosidase (SRF2g9), four GHF3 β-glucosidases (SRF2g14, SRF2g18, LAB25g2 and LAB20g4), a GHF10 β-xylanase (LAB20g10), an α-amylase/β-xylanase/carbohydrate esterase (SRF2g13) and a GHF43/62/32/68 β-1,4-xylosidase/α-L-arabinofuranosidase (LAB20g2). These putative proteins exhibited from 55 to 92% amino acid (AA) sequence identities and 70-96% AA sequence similarities (provided in Additional file 1: Table S1) to homologous proteins found in genomes of bacteria of the Bacteroidetes phylum (e.g. *Prevotella ruminicola*). Those microbes are known to be abundant in the ruminal environment and are thought to play key roles in the breakdown of proteins and carbohydrate polymers [25].

### Catalytic parameters of GHF3 β-glucosidases from cow rumen

The four putative GHF3 β-glucosidases (SRF2g14, SRF2g18, LAB20g4 and LAB25g2) were cloned, expressed in *Escherichia coli* BL21 (DE3) and purified. Furthermore, their activities were tested with a battery of substrates and the half-saturation (Michaelis) coefficient (*K*_m_), the catalytic rate constant (*k*_cat_), the catalytic efficiency (*k*_cat_/*K*_m_) values and/or specific activities, were determined.

As shown in Table 1, in terms of catalytic efficiencies, *p*NPβG and *p*NPβC were the preferred substrates for 3 and 1 β-glucosidases, respectively: [(*k*_cat_/*K*_m_)]_*p*NPβG_/[(*k*_cat_/*K*_m_)]_*p*NPβC_ factor of ~26/1 (for SRF2g14), 0.7/1 (for SRF2g18), 356/1 (for LAB20g4) and 2/1 (for LAB25g2). All enzymes showed higher *k*_cat_ values for *p*NPβG (from 2.0 to 7.3-fold), and therefore, the divergence in catalytic efficiencies were mainly due to differences in substrate affinity: 2 enzymes (SRF2g14 and LAB20g4) showed a significantly (from 14 to 49-fold) lower *K*_m_ value for *p*NPβG substrate whereas LAB25g2 showed similar affinities and SRF2g18 circa 4 times higher affinity for *p*NPβC. The catalytic efficiency (*k*_cat_/*K*_m_) while using the non activated substrate cellobiose was from 44 to 1026-fold lower than that found for the best *p*NP substrate, mainly due to a significant increase of *K*_m_ values (from 7 to 260-fold, depending on the enzyme) for the disaccharide. Enzymes SRF2g14 and SRF2g18 were the most- and less-efficient, respectively, for the hydrolysis of the disaccharide (*k*_cat_/*K*_m_ ratio for SRF2g14:SRF2g18 of 14:1).

**Table 1:** Kinetic parameters of the purified β-glucosidases

**Enzyme**	**Substrate**	***K***_**m**_**(mM)**^**a**^	***k***_**cat**_**(s**^**-1**^**)**^**a**^	***k***_***cat***_***/K***_**m**_**(s**^**-1**^** M**^**-1**^**)**^**a**^
SRF2g14	*p*NPβGlu	0.034 ±0.003	0.37 ± 1.34	10.76 × 10^3^
	*p*NPβC	0.48 ±0.054	0.19 ± 0.36	4.16 × 10^2^
	Cellobiose	8.02 ±0.939	0.38 ± 0.55	47.64
SRF2g18	*p*NPβGlu	1.73 ± 0.004	0.27 ± 0.32	1.59 × 10^2^
	*p*NPβC	0.48 ±0.072	0.11 ± 0.22	2.32 × 10^2^
	Cellobiose	25.51 ±2.46	0.09 ± 0.31	3.35
LAB20g4	*p*NPβGlu	0.03 ±0.003	0.88 ± 4.05	29.49 × 10^3^
	*p*NPβC	1.47 ± 0.13	0.12 ± 0.20	82.73
	Cellobiose	7.80 ± 0.67	0.22 ± 0.35	28.75
LAB25g2	*p*NPβGlu	0.45 ± 0.06	0.41 ± 0.68	9.20 × 10^2^
	*p*NPβC	0.39 ± 0.03	0.17 ± 0.36	4.38 × 10^2^
	Cellobiose	4.88 ± 0.59	0.10 ± 0.23	20.84

^a^Parameters were determined at 40°C and pH 5.6 (50 mM sodium acetate) using substrate and enzyme concentrations as described in Methods.

The purified recombinant β-glucosidases were also assayed for their activities toward different polymeric substrates. By meaning of specific activity determination, β-glucosidases hydrolysed all short cello-oligosaccharides tested (degree of polymerisation [DP] from 2 to 5), with longer substrates being preferred (Table 2). According to the activity level the following order could be established: LAB25g2 > SRF2g18 > SRF2g14 > LAB20g4, with LAB25g2 being 9-fold more active against cellopentaose as compared to LAB20g4. Two out of four β-glucosidases exhibited activity against lichenan, namely, SRF2g14 and LAB20g4 (Table 2), suggesting that they are able to hydrolyze substrates with mixed β-1,3/4 linkages; a [(mU mg^-1^)]_cellobiose_/[(mU mg^-1^)]_lichenan_ factor of ~6/1 (for SRF2g14) and 13/1 (for LAB20g4) was observed. No activity was detected using avicel, filter paper, as well as towards substrates without β-1,4 linkages such as β-1,3 glucan, or mixed β-1,3/6 linkages like laminarin. Accordingly, the enzymes showed a clear preference for short cello-oligosaccharide substrates, which may likely be produced in natural settings from the cellulose components of plant cell walls due to the action of glucanases.

**Table 2:** Specific activity of the purified β-glucosidases towards various cello-oligosaccharides (degree of polymerisation [DP] from 2 to 5) and carbohydrate polymers

**Substrate**	**Specific activity (U mg**^**-1**^**)**			
	**Pure β-glucosidases**			
	**SRF2g14**	**SRF2g18**	**LAB20g4**	**LAB25g2**
Cellobiose^a^	369.9 ± 0,04	852.8 ± 0.03	305.9 ± 0.05	1537*.*2 ± 0.01
Cellotriose^a^	317.6 ± 0.04	789.4 ± 0.03	60.2 ± 0.01	2170*.*5 ± 0.02
Cellotetraose^a^	343.0 ± 0.01	944.4 ± 0.01	283.9 ± 0.01	2154*.*3 ± 0.01
Cellopentaose^a^	397.4 *±* 0.001	1154.8 *±* 0.03	313.5 *±* 0.02	2682*.*6 ± 0.01
Lichenan^a^	60*.*8 ± 0.13	n.d	22.8 ± 0.16	n.d
	*E. coli* BL21 (DE3) cell extracts expressing β-glucosidases^b^			
*p*NPβGlu^c^	0.39	5.1×10^-3^	0.40	1.5
Cellobiose^d^	115.2	16.2	105.9	231.5

^a^Reaction assays contain 2 μg pure enzyme, 200 μL 50 mM sodium acetate buffer (pH 5.6) and 100 μL of a 100 mM cellobiose stock solution (final concentration of cellobiose of 20 mM). For lichenan a concentration of 10 mg ml^-1^ was used. Samples were incubated at 40°C during 5 h, after which the glucose concentration was measured using the glucose oxidase kit (Sigma Chemical Co., St. Louis, MO, USA). All of the measurements were analysed in triplicate; n.d. no activity detected.

^b^Quantification of proteins in *E. coli* BL21 (DE3) cell extracts expressing β-glucosidases was performed according to BCA Protein Assay kit (Thermoscientific), and are as follows: 552, 426, 458 and 508 μg ml^-1^ for cell extracts of SRF2g14, SRF2g18, LAB20g4 and LAB25g2, respectively.

^c^Activity measured by using 100 μl cell extract, 50 μl *p*NPβG solution (0.22% w/v) and 150 μL buffer McIlvane (100 mM, pH 5). Incubation: 20 min at 40°C, after which 250 μl Na_2_CO_3_ (1 M) was added and absorbance measured at 405 nm. Specific activity was measured as follows: mU ml^-1^ enzyme = A/KtV (A = absorbance at 405 nm; K = 0.0328 (value determined by calibration); V = volume in ml. The standard deviation was lower than 5%.

^d^Reaction assays contain 200 μl enzyme extract, 200 μl McIlvaine buffer and 100 μl of a 100 mM cellobiose stock solution (final concentration of cellobiose of 20 mM). Sample were incubated at 40°C during 4 h, alter which the glucose concentration was measured using the glucose oxidase kit (Sigma Chemical Co., St. Louis, MO, USA). The standard deviation was lower than 5%.

### Effect of temperature and pH on specific activity and stability

**Figure 1:**
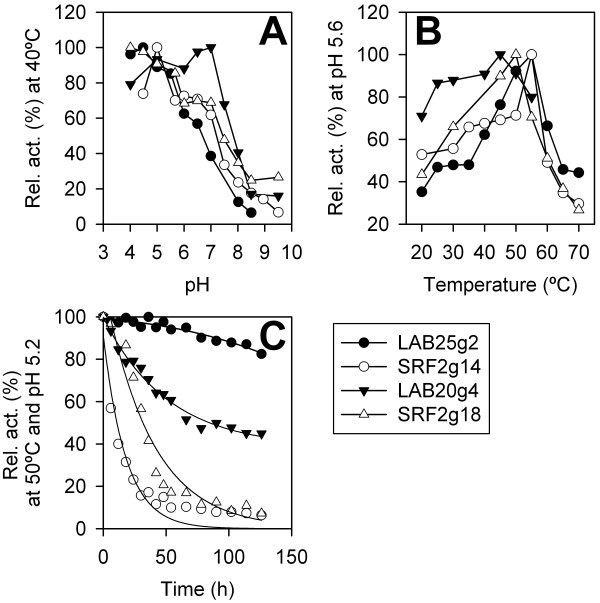
**pH (A) and temperature (B) optima and stability (C) of the purified β-glucosidases.** The parameters were determined using *p*NPβG as the substrate. (**A**) The optimum pH was determined in the range of pH 4.0–8.5 at 40°C. The buffers (20 mM) used were as follows: acetate (pH 4.0-6.0), MES (pH 6.0-7.0), HEPES (pH 7.0-8.0) and Tris–HCl (pH 8.0-8.5). (**B**) For the optimum temperature determination, the pH was adjusted to 5.6 (sodium acetate 20 mM). In both cases, the *k*_cat_ value was determined using an [enzyme] ranging from 0 to 12 nM and a substrate concentration of 70 mM. Activity at 100% refers to the *k*_cat_ values described in Table 1. (**C**) The time lost normalised quantification of the β-glucosidase activity levels (with *p*NPβG) at conditions reassembling the supplementation assays (100 ml flasks with 12 g of pre-treated biomass and 5 g β-glucosidase solution at 25 U ml^-1^ (sodium acetate 20 mM), 50°C, pH 5.2; for details see Methods). For activity test at different time points, protein solution was separated from soil particles by ultrafiltration through low-adsorption hydrophilic 500000 NMWL cutoff membranes (regenerated cellulose, Amicon) and directly used for activity tests using *p*NPβG as the substrate. All measurements were analysed in triplicate as described in Methods. Error bars are not indicated because the standard deviation was lower than 5%.

The optimum activity for GHF3 β-glucosidases was observed within a narrow range of temperatures, between 45 and 55°C, and within a narrow pH range (4.0-7.0) being most active at pH close to 5.0 (Figure 1). Stability assays (measured at 50°C and pH 5.2 in conditions reassembling the supplementation assays – see details below) revealed LAB25g2 to be substantially stable, with only 18% loss of activity after 5 days of incubation; under similar conditions, the half life for SRF2g14, SRF2g18 and LAB20g4 was 18.06 ± 1.9 h, 37.5 ± 3.9 h and 136.1 ± 9.4 h, respectively (Figure 1C). The stability features, namely for LAB25g2 enzyme, are better than those of many other microbial β-glucosidases (provided in Additional file 2: Table S2).

### Enzymatic conversion of lignocellulose in supplementation assays

Saccharification of the cellulose in biomass results in sugar-rich liquid streams useful for the production of bio-ethanol [5]. To estimate the capability of herein discovered β-glucosidases from cow rumen in such process, crude extracts of *E. coli* BL21 (DE3) cells expressing β-glucosidases were produced as described in Methods and used in saccharification of pre-treated corn stover biomass. First, their protein concentrations and activity levels using *p*NPβG and cellobiose as substrates, were examined. As shown in Table 2 the cell extract containing β-glucosidase LAB25g2 showed the higher activity level toward both substrates which agrees with activity tests using pure proteins; using cellobiose (58 g l^-1^) as substrate, β-glucosidase LAB25g2 produced approx. 2.5 g glucose mg^-1^ cell extract h^-1^ at 40°C and pH 5.0. The differences in activity performance for the three other β-glucosidases when using cell extracts as compared to assays using pure proteins may be due to differences in expression level and protein solubility during production in *E. coli* BL21 (DE3), which was evidenced by SDS-PAGE analysis (not shown).

Crude extract of β-glucosidase LAB25g2 was then selected to investigate its effect in supplementation assays under the standard industrial conditions described in Methods: 50°C and pH 5.2. Crude cell extracts containing LAB25g2 (31.25 units g^-1^ dry biomass, using *p*NPβG as substrate, or 89.28 units g^-1^ glucan) were supplemented to β-glucosidase-deficient commercial cellulase product Celluclast, derived from *Trichoderma reesei* (Novozymes A/S, Bagsvaerd, Denmark) (5 units in the basis of *p*NPβG hydrolysis g^-1^ dry biomass); control reactions without LAB25g2 as well as with two commercial β-glucosidase preparations [G0395 from almond (Sigma Chemical Co., St. Louis, MO, USA) and E-BGOSAG from *Agrobacterium* sp. (Megazyme; Bray, Ireland)] were also performed under similar conditions. It should be noticed that the glucan content of the biomass used is about 35% of dry matter content (measured accordingly to the Standard Biomass Analytical Procedures; http://www.nrel.gov/biomass/analytical_procedures.html), that is, 350 g glucan kg^-1^ dry matter content.

**Figure 2:**
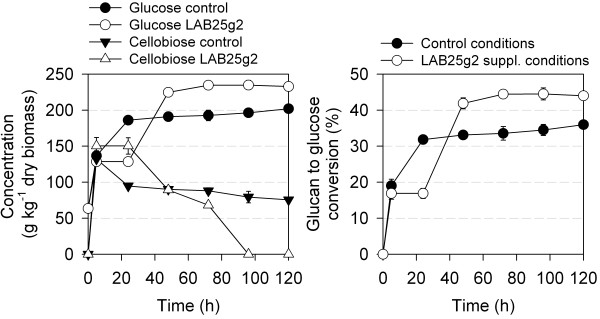
**Supplementation of commercial cellulases with β-glucosidase LAB25g2.** The dosage of β-glucosidases was performed on the basis of same activity towards pNPβG, namely, 31.25 U g^-1^ dry biomass (minimum dose of LAB25g2 to achieve saccharification of 20% w/w pre-treated corn stover under assay conditions). Saccharification of lignocellulose is shown by meaning of glucose and cellobiose concentration in assay tests (**A**) or glucan to glucose conversion (**B**). Hydrolysis reactions: 100 ml flasks, pH 5.2, 12 g of pre-treated corn stover, 3 g of enzymatic mixture (Celluclast) and 5 g LAB25g2 β-glucosidase solution at 25 U ml^-1^ (in the basis of *p*NPβG assay). Control reactions in the absence of LAB25g2 were performed. Commercial β-glucosidases G0395 (from almond) and E-BGOSAG (from *Agrobacterium* sp.) were used as control test as for LAB25g2. For that protein solutions at 25 U ml^-1^ were prepared and added to reaction mixtures to achieve a final activity of 31.25 U g^-1^ dry biomass (*p*NPβG), as for LAB25g2. Specific activity of those preparations is shown in Additional file 2: Table S2. In both cases, results were similar to those found in the control experiment without β-glucosidase and are not shown. Determination of glucose and cellobiose concentrations was followed by RID-HPLC. Glucose concentration was also measured by the glucose oxidase-peroxidase D-Glucose Assay Kit (Megazyme). All measurements were analysed in triplicate as described in Methods. Error bars are indicated. Note: under similar dosage conditions β-glucosidase Novo-188 (Novozymes A/S) consumed all cellobiose in the assay after 24 h (not shown).

Figure 2A shows the enzymatic hydrolysis profiles of control and supplementation assays. As shown, LAB25g2 addition resulted in higher production of glucose and higher cellobiose consumption, which was noticeable above 48 h of treatment, and optimal at 96 h: glucose production increased from 196.3 ± 6.0 to 234.8 ± 6.4 g glucose kg^-1^ dry biomass, most likely as a consequence of the improved cellobiose hydrolysis by the addition of LAB25g2 β-glucosidase. Thus, whereas control reaction contained 79.3 ± 8.0 g cellobiose kg^-1^ dry biomass, no appreciable amount was detected in supplemented reaction. It should be noticed that other commercial β-glucosidase preparations such as those from almond (G0395; Sigma Chemical Co.; 42 and 2.3 units mg^-1^ using *p*NPβG and cellobiose, respectively at 50°C and pH 5.0) and *Agrobacterium* sp. (E-BGOSAG; Megazyme; (119 ± 8) x 10^3^ and 0.29 units mg^-1^ using *p*NPβG and cellobiose, respectively at 50°C and pH 5.0), did not produce hydrolysis improvements under similar conditions as those used with LAB25g2 (not shown). To calculate the saccharification yield at an indicated time (SYt) we applied the following formula: SYt (%) = [Glucose at t (g kg^-1^) – Glucose initial (g kg^-1^)] / [Glucose maximal (g kg^-1^)] x 100. According to this equation, LAB25g2 increased the final hydrolysis yield by a factor of 20% (44.5 ± 1.7% vs. 34.5 ± 1.5% in control conditions) after 96–120 h treatment (Figure 2B). This suggests the potential of accessory β-glucosidase activity from cow rumen to improve the enzymatic hydrolysis of pre-treated corn stover.

### 3D structural analysis of biochemically characterised GHF3

**Figure 3:**
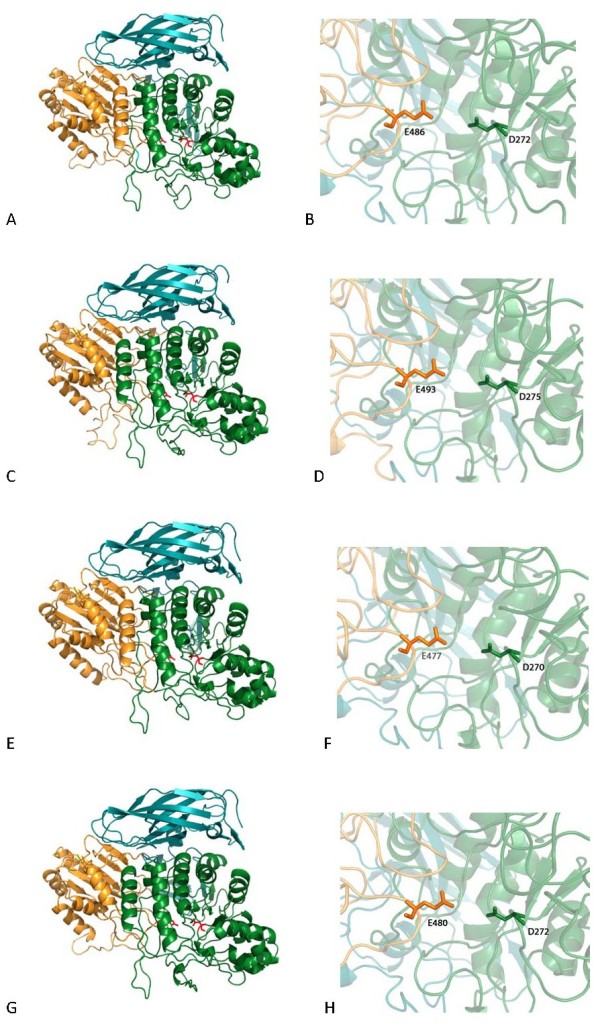
**Structural models of β-glucosidases characterised in this work:LAB25g2 (Aand B,) SRFg14 (C and D**)**, SRFg18 (E and F) and LAB20g4 (G and H).** Panels **A**, **C**, **E** and **G** show overall representation of the enzyme structures. The three protein domains, α/β barrel, α/β sandwich and fibronectin-like, are coloured green, yellow and blue, respectively. Panels **B**, **D**, **F** and **H** show closeup of the catalytic centres, indicating the position of the catalytic nucleophile (Asp) and proton donor (Glu).

Enzymes belonging to family GHF3 are multi-domain proteins whose domains may differ in number and arrangement [26-28]. The four enzymes analysed in this work show good end-to-end sequence alignment (ca. 50% identity) and consequently high structural resemblance with the β-glucosidase of *Thermotoga napolitana*[26]. As shown in Figure 3, they are composed by two domains, an α/β sandwich and an α/β barrel that contribute each one of the two catalytic residues, and a carboxy-terminal fibronectin-like domain, present in some but not all enzymes of this family. As expected by the high similarity of the four protein sequences, the modelling yielded nearly superimposable structures with no obvious trait (eg. amino acid insertions, loops) that could account for the observed difference in catalytic efficiency. Therefore, the structural basis of the superior performance of LAB25g2 may rely on subtle amino acid differences, possibly of residues located in the vicinity of the catalytic site, involved in substrate binding. The models should be helpful in pinpointing these residues whose role can be tested by site directed mutagenesis.

## Discussion

In the present work, an expression metagenomic library was used to identify β-glucosidases in the DNA fragments from uncultured microorganisms populating the cow rumen. We detected four β-glucosidases that have been subcloned, expressed, purified and characterised; these enzymes were likely derived from representatives of the genus *Prevotella ruminicola* (Bacteroidetes phylum) known to be abundant in the ruminal environment [25].

β-Glucosidases could be employed in combination with low dosages of commercial cellulase enzymes, in applications such as bio-ethanol production [14,16], which requires efficient enzyme cocktails to achieve a high degree of conversion of lignocellulose [9,10,29-31]. Bioconversion of lignocellulose into bio-ethanol consists of four major steps: pre-treatment, hydrolysis, fermentation, and product separation/distillation [32]. Cellulases and hemicellulases act synergistically in the second step of enzymatic hydrolysis. In this study, LAB25g2 β-glucosidase showed synergistic effects with Celluclast, one of the most common enzymatic cocktails actually produced (Novozymes A/S, Bagsvaerd, Denmark), which can release fermentable sugars from oligosaccharides obtained during the breakdown of lignocellulose by endo-hydrolases. Such synergistic effect is of a special interest as it is known that cellulose is difficult to be hydrolysed even after pre-treatment [2,3]. Although the enzyme LAB25g2 did not show appreciable activity towards filter paper where the cellulose is mostly crystalline, it would promote the hydrolysis of short- to medium-sized cello-oligosaccharides (Table 2) that are likely produced by endo- and exo-glucanases present in the commercial cocktail. The final concentration of glucose after 96 h of treatment was 234.8 ± 6.4 g glucose kg^-1^ dry biomass which is approximately equal to 20% increase in the glucan hydrolysis yield. More interestingly, the enzyme was also able to contribute to the complete hydrolysis of cellobiose in the slurry (containing 20% dry biomass) after 96 h hydrolysis. This result seems to be highly valuable, since *Saccharomyces* strains are commonly used for fermentation of ethanol and they utilize glucose more efficiently than cellobiose, because they have no β-glucosidases [7]. Also, LAB25g2 displayed a high stability under experimental conditions (t_1/2_ > 5 days at 50°C and pH 5.2; Figure 1C) likely similar to those required for the industrial bio-ethanol production.

To understand the reasons of the high performance of LAB25g2 in supplementation assays, a detailed comparison of the properties and kinetic studies of about hundred β-glucosidases from various sources, in which affinity constants (*K*_m_), maximal specific activity (V_max_), catalytic rate constant (*k*_cat_), catalytic efficiency (*k*_cat_/*K*_m_) and/or specific activities (units mg^-1^) reported for assays using *p*NPβG, *p*NPβC and cello-oligosaccharides ranging from cellobiose to cellohexaose, was undertaken (data provided in Additional file 2: Table S2). They include those from known bacteria, archaea, yeasts, fungi, plants and insects, as well as from unknown and uncultured microbial resources. The β-glucosidase properties from *Aspergillus niger*, generally used to complement the cellulolytic cocktail of *Trichoderma reseei*[13] actually used in hydrolysis tests and commercial preparations, were also included. Compared with reported or commercially available β-glucosidases (*K*_m_: 0.004-68 mM for *p*NPβG; 4.8-17.6 mM for *p*NPβC; and 0.31-66 mM for cellobiose) our enzymes exhibit similar affinity towards synthetic *p*NPβG (from 0.034 to 1.73 mM) and *p*NPβC (from 0.48 to 1.47) as well as for cellobiose (from 4.88 to 25.51 mM). The *k*_cat_ values (≤ 0.88 s^-1^) were in the same range or lower than those reported (from 0.022 to 87400 s^-1^ for *p*NPβG, 18.2-17500 s^-1^ for *p*NPβC and 4.5-699 s^-1^ for cellobiose). However, the enzyme kinetic parameters from previous studies demonstrated significant differences in their hydrolytic capacity against natural cello-oligosaccharides, with notably better values for the β-glucosidases reported in present study. Thus, for the enzyme LAB25g2, the specific activity for cellobiose (1537.2 units mg^-1^ when used as pure protein or 231.5 units mg^-1^ as crude *E. coli* BL21 (DE3) cell extracts expressing β-glucosidase) was up to 38000 fold higher, as compared with the reported or commercially available β-glucosidases (see Additional file 2: TableS 2); only the β-glucosidase from the fungus *Penicillium funiculosum* NCL1 (1796 units mg^-1^) had a close activity, followed at much lower extent, by that of *Aspergillus oryzae* (938 units mg^-1^). Additionally, it is noteworthy that LAB25g2 did show 27–1166 fold higher specific activity towards cellotriose-cellopentaose (up to 2682.6 ± 0.01 units mg^-1^), as compared to known β-glucosidase, with that of *Aspergillus oryzae* (512 units mg^-1^), being the most-active among enzymes and commercial preparations reported so far. Compared with Novozymes 188, a β-glucosidase derived from *Aspergillus niger* and one of the most commonly used β-glucosidases in lignocellulose conversion [15], we found that crude extract and pure preparations of LAB25g2 were 7 and 46 fold, respectively, more active towards cellobiose (see Additional file 2: Table S2). Accordingly, to the best of our knowledge, LAB25g2 has the highest activity reported for short-chain cello-oligosaccharides. This finding is of a special significance, as the activity towards cello-oligosaccharides has only been reported for a limited number for β-glucosidases.

Results further indicated that LAB25g2 crude extract preparation was more effective that two commercial β-glucosidases (selected on the basis of their high β-glucosidase activity as compared to other commercially available preparations) for the saccharification of pre-treated corn stover, as they both produced similar results as the control tests without supplement. This may be described to the activity characteristics differences. Thus, using *p*NPβG as substrates both enzymes were 28 (from almond) and 79000 (from *Agrobacterium* sp.) times more active than crude extract preparation of LAB25g2; however, using cellobiose, almond and *Agrobacterium* sp. preparations were 100–798 less active that LAB25g2 preparation. Additionally, whereas almond β-glucosidase did show low activity at 50°C and pH 5.0 that from *Agrobacterium* sp. was quite stable (Additional file 3: Figure S1), indicating that both activity and stability features accounted for the LAB25g2 performance. It has been also reported that Novozymes 188 decreased the remaining cellulose concentration in supplementation assays using *T. reesei* cellulase by 10.1% (dry matter content of 10%; 37°C, 120 h) at low dosage (3.76 units g^-1^ dry biomass, using *p*NPβG as substrate) [15]; this factor is about half of that produced by LAB25g2, although it should be taken into consideration that in our experiments 8.3-fold higher β-glucosidase supplemented activity was used. Under similar dosage conditions β-glucosidase Novo-188 (Novozymes A/S) was found to consume all cellobiose in our experimental conditions after 24 h (not shown), whereas LAB25g2 required several days. Other characteristics of LAB25g2 β-glucosidase (i.e., optimum temperature, 50°C; pH 4.5-5.5, and high stability under supplementation assay conditions (50°C, pH 5.2)) suggest this enzyme has an outstanding position among the reported β-glucosidases isolated from different sources. Most likely, the high performance of LAB25g2 towards oligosaccharides occurring during the breakdown of lignocellulose by exo- and endo-hydrolases, together with its thermostability, seem to be major factors contributing to the biomass hydrolysis improvement when used in combination with the β-glucosidase-deficient commercial cocktail Celluclast (Novozymes A/S).

The discovery of a high performance novel β-glucosidase for the saccharification of lignocellulose under industrial operational conditions is a clear example of the utility of function-centred enzyme discovery in complex microbial communities. The natural selection by the great polymeric substrate diversity imposed on a complex microbial community is likely a key factor driving the evolution of family 3 β-glucosidases, which has been recognized to represent about 19% of the total carbohydrate hydrolases in the bovine rumen [25]. Taking into account that many proteins of this family share a significant degree of homology with enzyme LAB25g2 reported here, we suggest that the enzymatic potential of the microorganisms populating animal gastrointestinal tracts remains underestimated and underexploited and that animal digestomes may be a potent bio-resource for novel lignocellulases for bio-ethanol production. Although, the yield improvements reported here are far from being scaled up at the industrial level (and are lower than that reported for Novo-188 [15], one of the most commonly used β-glucosidases in lignocellulose conversion), the present study highlights the need for more extensive experimental work to accurately identify enzymes potentially applicable for bio-fuel production [33]. That might also result in the production of efficient setups or *a-la-carte* cocktails mixed in-house by own technological facilities upon demand and upon the choice of the feedstock.

## Conclusions

The catalytic performance of four β-glucosidases from cow rumen was examined for their applicability for the saccharification of lignocellulose in combination with a β-glucosidase-deficient commercial cellulase cocktail. One of the β-glucosidases was demonstrated to be effective and thermo-stable for the enzymatic breakdown of pre-treated corn stover, with a substantial role in cellobiose hydrolysis and the concomitantly increase production of glucose. Having said that, recent developments for the production of commercial cocktails with improved Î²-glucosidases have been achieved and many factors account for the cost-effective production of bio-ethanol; whatever the case, to the best of our knowledge, this study provides the first clear experimental evidences that β-glucosidases from ruminal bacterial metagenome are of a great potential interest for the efficient hydrolysis of lignocellulosic biomass, and confirms the necessity of isolating and characterizing new β-glucosidases from this bio-resource.

## Methods

### Materials and strains

Chemicals, biochemicals and solvents were purchased from Sigma Chemical Co. (St. Louis, MO, USA) and were of p.a. (pro-analysis) quality. The oligonucleotides used for DNA amplification, mutagenesis and sequencing were synthesised by Sigma Genosys Ltd. (Pampisford, Cambs, UK). The restriction and modifying enzymes were from New England Biolabs (Beverly, MA, USA). The Ni-NTA His·Bind chromatographic media were from QIAGEN (Hilden, Germany). The *E. coli* EPI300-T1^R^ strain (Epicentre Biotechnologies; Madison, WI, USA) used for the fosmid library construction and screening and *E. coli* GigaSingles for the cloning and BL21 (DE3) for the expression using the pET-41 Ek/LIC vector (Novagen, Darmstadt, Germany) were cultured and maintained according to the recommendations of the suppliers. *p*NP-β-D-glucopyranoside (*p*NPβG), *p*NP-β-D-cellobioside (*p*NPβC), the cello-oligosaccharides (ranging from cellobiose to cellopentaose), the D-glucose assay kit and the almond β-glucosidase G0395, were all provided by Sigma Chemical Co. (St. Louis, MO, USA). *Agrobacterium* sp. β-glucosidase E-BGOSAG was provided by Megazyme (Bray, Ireland).

### Metagenomic library construction and enzyme screening

Rumen contents were collected from four rumen-fistulated, non-lactating Holstein cows (average weight of 731 kg) housed at Trawsgoed experimental farm (Aberystwyth, Ceredigion, Wales). Samples were retrieved under the authorities of the UK Animal (Scientific Procedures) Act (1986). The animals were fed a diet composed of a mixture of grass silage and straw (75:25) *ad libitum* and ~1 kg of sugar beet nuts at 07:00 am; the cows had constant access to fresh water. Sampling was done 2 h after concentrate feeding. Approximately 1 liter of mixed liquid and solid ruminal content was sampled from each cow pooled together to give a composite sample from all cows. The samples were then processed to produce two fractions: strained ruminal fluid (SRF) and liquid-attached bacteria (LAB). For SRF retrieval, total ruminal content was strained through four layers of muslin in order to remove large particles, and SRF was then frozen at −80°C until use. For LAB retrieval, approximately 1 liter of mixed total rumen content was hand squeezed to get rumen liquor, and the solid fraction was put in a large foil tray. The liquid fraction was spun at 2,000 × *g*, 10 min, 4°C (MSE Europa 24 M, Berthold Hermle KG, Weisbaden, Germany); the supernatant was then strained through a 1 mm^2^ pore-sized nylon mesh to remove feed particles, and spun again at 13,000 × *g*, 25 min, 4°C. The pellet was washed in a saline solution (made from 180 g NaCl dissolved in 20 L distilled water), and subsequently centrifuged at 13,000 x *g*, 25 min, 4°C. The pellet was re-washed with distilled water, and spun down at 13,000 x *g*, 15 min, 4°C. The pellet, containing LAB, was then transferred into a sterile jar and kept at −80°C until DNA extraction.

Total DNA was extracted from LAB and SRF microbial communities as described previously [19], using the G’NOME® DNA Isolation Kit (Qbiogene, Heidelberg, Germany). Purified and size-fractioned DNA was ligated into the pCCFOS fosmid vector and further cloned in *E. coli* EPI300-T1^R^ according to the instructions of Epicentre Biotechnologies (WI, USA) and a procedure described earlier [22]. Fosmid clones (16,896) harbouring approximately 600 Mbp of community genomes were arrayed using the QPix2 colony picker (Genetix Co., UK) and grown in 384-microtitre plates containing Luria Bertani (LB) medium with chloramphenicol (12.5 μg ml^-1^) and 15% (*v/v*) glycerol and stored at −80°C.

To screen for GH activity, the clones were plated onto large (22.5 × 22.5 cm) Petri plates with LB agar containing chloramphenicol (12.5 μg ml^-1^) and the induction solution (Epicentre Biotechnologies; WI, USA) as recommended by the supplier to induce a high fosmid copy number. An array of 2,304 clones per plate was set. After overnight incubation, each library was screened for the ability to hydrolyse *p*NPβG and *p*NPβC. For screens, the plates (22 × 22 ccm) were covered with an agar buffer substrate solution (40 ml of 50 mM sodium acetate, pH 5.6, 0.4% agarose and 5 mg ml^-1^ of *p*NPβG and *p*NPβC as substrates). Positive clones appeared due to the formation of a yellow colour. The positive clones were selected and their DNA inserts fully sequenced with a Roche 454 GS FLX Ti sequencer (454 Life Sciences, Branford, CT, USA) at Life Sequencing S.L (Valencia, Spain).

### Cloning, expression, purification and characterisation of plant polymeric substance hydrolases

The gene cloning was performed by PCR using goTaq® DNA polymerase and custom oligonucleotide primers. To amplify the hydrolase genes, the corresponding fosmid was used as the template; the vectors and the pairs of primers are described as follows: SRF2g14 Fwd 5′-gacgacgacaagATGAAGAAGACTCTGTTTTTCGCCTTTGGC-3′ and SRF2g14 Rev 5′-gaggagaagcccggTTATCGTTTCAGGAGGTTCATCTGAACCTGTGG-3′ (total gene length 2361 bp); SRF2g18 Fwd 5′-gacgacgacaagATGAGAAAATCGATTCATCAGATTAGTTTGG-3′ and SRF2g18 Rev 5′-gaggagaagcccggTTATCGCTTCAGTAGGTTGAGTTTCAATTTG-3′ (total gene length 2343 bp); LAB20g4 Fwd 5′-gacgacgacaagATGAAGAAAATCATGCTCCTCTCCGCCACC-3′ and LAB20g4 Rev 5′-gaggagaagcccggTTATTTCATTAAGAGCACGCGGTTGGCGGGC-3′ (total gene length 2295 bp); LAB25g2 Fwd 5′gacgacgacaagATGAAAAAATTACTAACAATTTGCTTCGTAGC-3′ and LAB25g2 Rev 5′-gaggagaagcccggTTACTGTTTCAGAAGATTGAGTTTCTGTTTTG-3′ (total gene length 2340 bp). The PCR conditions were as follows: 95°C for 120 s, followed by 30 cycles of 95°C for 30s, 55°C for 45 s and 72°C for 120 s, with a final annealing at 72°C for 500 s. The PCR products were analysed from agarose gel-purified using the Mini Elute Gel Purification Kit (Qiagen, Hilden, Germany) and cloned into pET-41-Ek/LIC (Novagen, Darmstadt, Germany) according to the manufacturer’s instructions. The resulting plasmids were introduced into the non-expressiong *E. coli* GigaSingles host and then, after plasmid extraction, into *E. coli* BL21 (DE3) for protein expression; the clones were selected on LB agar supplemented with kanamycin (30 μg ml^-1^). For the enzyme expression and purification, clones were grown overnight at 37°C with shaking at 200 rpm in 100 ml of LB medium containing kanamycin. Afterwards, 25 ml of this culture was used to inoculate 1 l of LB medium, which was then incubated for 4 hours to an OD_600nm_ of ~0.6 at 37°C. Protein expression was induced by 1 mM isopropyl-β-D-galactopyranoside (IPTG) followed by incubation for 16 h at 16°C. The cells were harvested by centrifugation at 5000 × *g* for 15 min to yield 2–3 g l^-1^ of pellet (wet weight). The cell pellet was frozen at −80°C overnight, thawed and resuspended in 10 ml 20 mM 4-(2-hydroxyethyl)piperazine-1-ethanesulfonic acid (HEPES) [pH 7.0] g^-1^ wet cells; Lysonase Bioprocessing Reagent (Novagen, Darmstadt, Germany) was then added (4 μl g^-1^ wet cells) and incubated for 30 min on ice with rotating mixing. The cell suspension was then sonicated for a total of 1.2 min and centrifuged at 15000 × *g* for 15 min at 4°C; the supernatant was retained. The His_6_-tagged enzymes were purified at 25°C after binding to a Ni-NTA His·Bind resin (Novagen, Darmstadt, Germany). The columns were pre-washed with 20 mM HEPES (pH 7.0), 0.15 M NaCl and 50 mM imidazole, and the enzymes were eluted with 20 mM HEPES (pH 7.0), 0.15 M NaCl and 250 mM imidazole. The monitoring of the enzyme elution was performed by SDS-PAGE and/or activity measurements, using standard assays. Pure enzyme thus obtained was treated with enterokinase, as recommended by the supplier (Novagen, Darmstadt, Germany) to remove the His tags. The purity was assessed as >95% using SDS-PAGE, which was performed with 12% (v/v) polyacrylamide gels, using a Bio-Rad Mini Protean system.

To produce *E. coli* BL21 (DE3) cell extracts expressing β-glucosidases, clones were grown overnight at 37°C with shaking at 200 rpm in LB medium containing kanamycin (30 μg ml^-1^). Afterwards, 0.3 ml of this culture was used to inoculate 30 ml of LB medium in a 250 ml flask, which was then incubated to an OD_600nm_ of ~0.8. Protein expression was induced by 1 mM IPTG followed by incubation for 4 h. The cells were harvested by centrifugation at 5000 × *g* for 15 min and re-suspended in 10 ml McIlvaine buffer (100 mM pH 5.0) containing lysozyme (0.1 mg ml^-1^). The cell suspensions were then sonicated until a completed cell lysis was observed by microscope and centrifuged at 15000 × *g* for 15 min; the supernatants were retained and their protein concentrations and activity levels using *p*NPβG and cellobiose as substrates, examined.

For the enzyme characterisation, the absorbance was measured using a BioTek Synergy HT spectrophotometer under the following conditions: [Enzyme] ranging from 0 to 300 nM, [substrate] ranging from 0 to 50 mM in 100 mM buffer, *T* = 40°C. For the hydrolysis of the *p*NP derivatives, the corresponding volume of a *p*NP derivative stock solution (150 mM) was incubated for 2–20 min in appropriate buffer and measured at 410 nm in 96-well microtiter plates [22]. The substrates tested included *p*NPβG and *p*NPβC. For cello-oligosaccharides the level of released glucose was determined, under the same conditions, using a glucose oxidase kit (Sigma Chemical Co., St. Louis, MO, USA). The initial rates were fitted to the Michaelis–Menten kinetic equation using non-linear regression to determine the apparent *K*_m_ and *k*_cat_; kinetic parameter calculations were performed based on the molecular masses listed in Additional file 1: Table S1. Substrate specificity was investigated also using carboxymethylcellulose (CMC), lichenan, barley glucan, laminarin and avicel (all from Sigma Chemical Co., [St. Louis, MO, USA]) and filter paper (Whatman, England). Enzymatic activity was quantified by measuring release of reducing sugars according to Miller [34] using [enzyme] of 20 nM, [substrate] of 10 mg ml^-1^ in 50 mM sodium acetate buffer pH 5.6, *T* = 40°C. In all cases, one unit (U) of enzyme activity was defined as the amount of enzyme producing 1 μmol of reducing sugars in 1 min under the assay conditions. Unless otherwise stated, standard assays were performed using [enzyme] of 20 nM, *p*NPβG] of 10 mg ml^-1^ in 50 mM sodium acetate buffer pH 5.6, *T* = 40°C.

The pH and temperature optima were determined in the range of pH 4.0–8.5 and 20–70°C in assays containing [enzyme] of 12 nM and [*p*NPβG]_o_ of 10 mg ml^-1^. Optimal pH was measured at 40°C; the following buffers (20 mM) were used: acetate (pH 4.0-6.0), 2-(*N*-morpholino)ethanesulfonic acid (MES) (pH 6.0-7.0), HEPES (pH 7.0-8.0), Tris–HCl (pH 8.0-8.5). pH was always adjusted at 25°C. Optimal temperature was determined in 50 mM sodium acetate buffer pH 5.6. All of the values were determined in triplicate and were corrected for the spontaneous hydrolysis of the substrate. The results shown are the averages of three independent assays ± the standard deviation.

pH and thermal stability assays were performed in 100 ml flasks with 12 g of pre-treated biomass (see below) and 5 g β-glucosidase solution at 25 U ml^-1^ (sodium acetate 20 mM), 50°C, pH 5.2). For activity test at different time points, protein solution was separated from soil particles by ultrafiltration through low-adsorption hydrophilic 500000 NMWL cutoff membranes (regenerated cellulose, Amicon) and directly used for activity tests using *p*NPβG as the substrate, using the standard protocol. All measurements were analysed in triplicate.

### *In silico* analysis of proteins

The MetaGeneMark tool with refined heuristic models for metagenomes (http://exon.gatech.edu/GeneMark/metagenome/index.cgi; [35]) was used to predict genes in the cloned DNA fragments. The deduced proteins were analysed using blastp and psi-blast [36] against the non-redundant databases. The translation products were further analysed for protein domains using the Pfam-A database [37]. Models of rumen β-glucosidases were obtained from the SWISS-MODEL server (http://swissmodel.expasy.org/) using the coordinates of *Thermotoga napolitana* β-glucosidase structure [26] (PDB identity: 2×41) as the template. Figures were created with PyMOL (http://www.pymol.org/).

### Supplementation of commercial cellulases with β-glucosidase LAB25g2

The activity of LAB25g2 on the hydrolysis of pre-treated corn stover biomass was tested by the supplementation of enzymatic hydrolysis reactions using a β-glucosidase-deficient commercial cocktail (Celluclast, Novozymes A/S, Denmark). Pre-treatment of the biomass was performed by diluted acid soaking followed by steam explosion as described previously [38]. Hydrolysis reactions were performed at 50°C in 100 ml flasks with 12 g of pre-treated biomass, 3 g of enzymatic mixture and 5 g LAB25g2 β-glucosidase solution at 25 U ml^-1^ (for details see Results), to reach 20 g final reaction mass. Pre-treated corn stover was adjusted to an initial pH of 5.2 by the addition of 2 N NaOH, reaching a dry matter content of 20% after addition of enzymes. Concentration of β-glucosidase activity during enzymatic hydrolysis was calculated in the basis of *p*NPβG assay (the standard substrate used in supplementation assays), and corresponds to 5 U g^-1^ dry biomass in the absence of LAB25g2 enzyme, and a supplementation of 31.25 U g^-1^ dry biomass in the presence of the enzyme. This was the minimum dose of LAB25g2 enzyme to achieve saccharification of pre-treated corn stover at a dry matter content of 20%, under conditions reassembling industrial operations. Three control reactions were considered: (i) assay in the absence of LAB25g2 enzyme; (ii) assay in the presence of commercial β-glucosidases G0395 (from almond; Sigma Chemical Co.) with specific activity, measured using *p*NPβG at 50°C and pH 5.0, of 0.042 ± 0.008 mU mg^-1^; (iii) assay in the presence of commercial β-glucosidases E-BGOSAG (from *Agrobacterium* sp.; Megazyme) with specific activity, measured using *p*NPβG at 50°C and pH 5.0, of 119 ± 8 mU mg^-1^. For control tests protein solutions at 25 U ml^-1^ were prepared and added to reaction mixtures as for the LAB25g2, to achieve a final activity value of 31.25 units g^-1^ dry biomass (in the basis of *p*NPβG assay). Enzymatic hydrolysis reactions were followed by determining glucose and cellobiose concentrations by HPLC (Agilent Technologies, 1200 Series) using a Refractive Index Detector (RID) and a Aminex HPX-87 H column. The system was operated at 65°C and a flow rate of 6 ml min^-1^ of 5 mM H_2_SO_4_ for product elution as described elsewhere [39]. Glucose concentration was also measured by the glucose oxidase-peroxidase D-Glucose Assay Kit (Megazyme; Bray, Ireland).

Note: the synthetic substrate *p*NPβG is specific for β-glucosidases, while not being hydrolyzed by cellobiohydrolases or endoglucanases. The numerous assays comparing activity towards *p*NPβG and cellobiose have demonstrated that both activities are, overall, linear functions one to another (β-glucosidases with high activity towards *p*NPβG also have high activity for cellobiose; for examples see Additional file 2: Table S2). Thus, supplementation per units of activity towards synthetic *p*NPβG for all enzymes used in saccharification ensured the same activity towards cellobiose in all cases, and for this reason, this substrate was used in our supplementation assays.

## Abbreviations

*p*NPβG: *p*-nitrophenyl-β-D-glucopyranoside; *p*NPβC: *p*-nitrophenyl-β-D-cellobioside; LAB: liquid-attached bacteria; SRF: strained ruminal fluid.

## Competing interests

The authors declare that they have no competing interests.

## Authors’ contributions

MF and PNG designed and coordinated the study and write the manuscript. MVP, LF-A, JG-M, AM, OVG, TNG, TYN, AW, MT, AR, SAH, CJN, JP and MF carried out the experiments and analysed the results. OVG and CJN contributed preparing the manuscript. All authors read and approved the final manuscript. MF and PNG contributed equally to this work.

## Supplementary Material

Additional file 1 **Table S1.**Annotation of genes predicted in fosmid clones from cow rumen. Selected fosmids were sequenced and predicted proteins ORFs were annotated by homology using BLAST aligment tool. The theoretical molecular weight (MW) and isoelectric point (p*I*) were calculated for the gene product using ExPASy ProtParam online tool (http://www.expasy.org/tools/pi_tool.html). Nucleotide and amino acid sequences are available at NCBI under acc. nr: JX163905, JX163906 and JX163904. Click here for file

Additional file 2 **Table S2.**Biochemical information of reported β-glucosidases. Data are based on bibliographic records that are specifically cited. Click here for file

Additional file 3 **Figure S1.**Temperature optima of commercial β-glucosidases from almond (G0395; Sigma Chemical Co.) and *Agrobacterium* sp. (E-BGOSAG; Megazyme). The parameters were determined using *p*NPβG (0.1 mg ml^-1^) as the substrate at pH 5.0 (sodium acetate). All measurements were analysed in triplicate and error bars are indicated. Left panel shows the specific activity and right panel the relative activity referred to that at 30°C. Click here for file
