# Supplementary material for: Microbial β-glucosidases from cow rumen metagenome enhance the saccharification of lignocellulose in combination with commercial cellulase cocktail
Source: Biotechnol Biofuels. 2012 Sep 21;5:73. doi: 10.1186/1754-6834-5-73 (PMC3477023; doi:10.1186/1754-6834-5-73)
Supplement: Additional file 1 — Table S1.Annotation of genes predicted in fosmid clones from cow rumen. Selected fosmids were sequenced and predicted proteins ORFs were annotated by homology using BLAST aligment tool. The theoretical molecular weight (MW) and isoelectric point (pI) were calculated for the gene product using ExPASy ProtParam online tool (http://www.expasy.org/tools/pi_tool.html). Nucleotide and amino acid sequences are available at NCBI under acc. nr: JX163905, JX163906 and JX163904. [file 1754-6834-5-73-S1.doc]

**Additional file 1: Table S1 Annotation of genes predicted in fosmid clones from cow rumen.** Selected fosmids were sequenced and predicted proteins ORFs were annotated by homology using BLAST aligment tool. The theoretical molecular weight (MW) and isoelectric point (p*I*) were calculated for the gene product using ExPASy ProtParam online tool (<http://www.expasy.org/tools/pi_tool.html>). Nucleotide and amino acid sequences are available at NCBI under acc. nr: JX163905, JX163906 and JX163904.

Fosmid SRF 2

| **Gene name** | **Strand** | **Left End** | **Right End** | **Length (AA)** | **Gene product name** | **MW (Da)*** | **pI*** | **Organism (best hit)** | **Expected Value (E), Identities (I), Positives (P)** | **Top homolog** |
| --- | --- | --- | --- | --- | --- | --- | --- | --- | --- | --- |
| SRF2g1 | + | 1 | 474 | 157 | glycogen debranching enzyme GlgX | 17905.82 | 4.42 | *Streptomyces sp. C* | E=6e-47; I=87/143 (60%); P=109/143 (76%) | ZP_07285673.1 |
| SRF2g2 | + | 450 | 698 | 82 | putative lipoprotein | 9695.09 | 9.84 | *Prevotella ruminicola 23* | E=1e-29; I=59/81 (72%); P=73/81 (90%) | YP_003575480.1 |
| SRF2g3 | + | 676 | 2406 | 576 | hypothetical protein PRU_2218 | 66948 | 9.34 | *Prevotella ruminicola 23* | E=e-178; I=318/533 (59%); P=388/533 (72%) | YP_003575479.1 |
| SRF2g4 | + | 2470 | 3942 | 490 | hypothetical protein PRU_2217 | 56439.11 | 4.81 | *Prevotella ruminicola 23* | E=0.0; I=397/474 (83%); P=431/474 (90%) | YP_003575478.1 |
| SRF2g5 | - | 3970 | 5418 | 482 | putative lipoprotein | 54598.01 | 5.94 | *Prevotella ruminicola 23* | E=0.0; I=385/475 (81%); P=435/475 (91%) | YP_003575477.1 |
| SRF2g6 | - | 5449 | 6852 | 467 | hypothetical protein PRU_2215 | 55321.6 | 8.58 | *Prevotella ruminicola 23* | E=0.0; I=328/468 (70%); P=379/468 (80%) | YP_003575476.1 |
| SRF2g7 | - | 6849 | 7877 | 342 | low specificity L-threonine aldolase | 38818.14 | 5.95 | *Prevotella bergensis DSM 17361* | E=e-129; I=221/341 (64%); P=280/341 (82%) | ZP_06006332.1 |
| SRF2g8 | + | 7876 | 8943 | 355 | conserved hypothetical protein | 40131.07 | 8.01 | *Prevotella ruminicola 23* | E=e-121; I=209/324 (64%); P=251/324 (77%) | YP_003575474.1 |
| SRF2g9 | - | 8966 | 10897 | 643 | **neopullulanase** | 73411.34 | 6.85 | *Prevotella ruminicola 23* | E=0.0; I=554/643 (86%); P=596/643 (92%) | YP_003575947.1 |
| SRF2g10 | - | 10922 | 14944 | 1340 | sensor histidine kinase/DNA-binding response regulator | 151498.52 | 5.12 | *Prevotella ruminicola 23* | E=0.0; I=1009/1345 (75%); P=1139/1345 (84%) | YP_003575946.1 |
| SRF2g11 | + | 15208 | 18258 | 1016 | conserved hypothetical protein | 111594.02 | 5.81 | *Prevotella ruminicola 23* | E=0.0; I=787/1000 (78%); P=879/1000 (87%) | YP_003575944.1 |
| SRF2g12 | + | 18314 | 19879 | 521 | putative lipoprotein | 58927.13 | 4.61 | *Prevotella ruminicola 23* | E=0.0; I=398/528 (75%); P=454/528 (85%) | YP_003575943.1 |
| **SRF2g13** | **+** | **19951** | **21159** | **402** | **isoamylase domain/esterase family protein** | **44853.33** | **6.32** | ***Prevotella ruminicola 23*** | **E=0.0; I=370/402 (92%); P=383/402 (95%)** | **YP_003575941.1** |
| **SRF2g14** | **+** | **21166** | **23526** | **786** | **glycol-hydro-3-superfamily/glycol-hydro-3C-superfamily protein** | **86202.55** | **4.93** | ***uncultured microorganism*** | **E=0.0; I=717/786 (91%); P=754/786 (95%)** | **ADR64671.1** |
| SRF2g15 | + | 23640 | 24941 | 433 | hypothetical protein BACSTE_01634 | 49486.23 | 9.55 | *Bacteroides stercoris ATCC 43183* | E=e-180; I=305/431 (70%); P=353/431 (81%) | ZP_02435388.1 |
| SRF2g16 | + | 24945 | 25736 | 263 | conserved hypothetical protein | 31043.57 | 6.80 | *Bacteroides thetaiotaomicron VPI-5482* | E=4e-73; I=134/261 (51%); P=181/261 (69%) | NP_813394.1 |
| SRF2g17 | + | 25748 | 26539 | 263 | hydrolase, Cof-like protein | 28574.64 | 4.60 | *Prevotella ruminicola 23* | E=1e-97; I=168/261 (64%); P=208/261 (79%) | YP_003575657.1 |
| **SRF2g18** | **+** | **26688** | **29030** | **780** | **glucan 1,4-beta-glucosidase** | **86363.04** | **6.94** | ***Prevotella ruminicola 23*** | **E=0.0; I=687/780 (88%); P=732/780 (93%)** | **YP_003575902.1** |
| SRF2g19 | - | 29016 | 30044 | 342 | NAD-dependent epimerase/dehydratase | 38311.69 | 5.72 | *Paenibacillus sp. oral taxon 786 str. D14* | E=e-127; I=219/335 (65%); P=260/335 (77%) | ZP_04851368.1 |
| SRF2g20 | - | 30041 | 31255 | 404 | hypothetical protein BACCELL_00512 | 44748.21 | 5.18 | *Bacteroides cellulosilyticus DSM 14838* | E=e-152; I=261/392 (66%); P=300/392 (76%) | ZP_03676187.1 |
| SRF2g21 | - | 31382 | 33298 | 638 | sensor histidine kinase | 72092.35 | 5.48 | *Prevotella ruminicola 23* | E=e-129; I=246/644 (38%); P=380/644 (59%) | YP_003574607.1 |
| SRF2g22 | - | 33295 | 33975 | 226 | sensor histidine kinase | 25516.67 | 5.64 | *Prevotella ruminicola 23* | E=6e-20; I=68/193 (35%); P=100/193 (51%) | YP_003574535.1 |
| SRF2g23 | - | 33982 | 34266 | 94 | hypothetical protein BACEGG_02987 | 10642.88 | 7.60 | *Bacteroides eggerthii DSM 20697* | E=1e-41; I=76/90 (84%); P=83/90 (92%) | ZP_03460176.1 |

Table S1 cont. Fosmid LAB25

| **Gene**  **name** | **Strand** | **Left**  **End** | **Right**  **End** | **Length (AA)** | **Gene product name** | **MW (Da)*** | **pI*** | **Organism (best hit)** | **Expected Value (E), Identities (I), Positives (P)** | **Top homolog** |
| --- | --- | --- | --- | --- | --- | --- | --- | --- | --- | --- |
| LAB25g1 | + | <3 | 434 | 143 | polysaccharide deacetylase family protein | 14937.02 | 10.12 | *Prevotella ruminicola 23* | E=3e-61; I=106/126 (84%); P=121/126 (96%) | YP_003575100.1 |
| **LAB25g2** | **+** | **437** | **2776** | **779** | **glucan 1,4-beta-glucosidase** | **85055.49** | **6.53** | ***Prevotella ruminicola 23*** | **E=0.0; I=662/767 (86%); P=709/767 (92%)** | **YP_003575101.1** |
| LAB25g3 | - | 2862 | 3299 | 145 | conserved hypothetical protein | 16477.16 | 8.88 | *Prevotella ruminicola 23* | E=4e-43; I=83/147 (56%); P=114/147 (77%) | YP_003575107.1 |
| LAB25g4 | - | 3296 | 3751 | 151 | biopolymer transport protein, ExbD/TolR family | 17431.79 | 5.04 | *Prevotella ruminicola 23* | E=6e-37; I=80/149 (53%); P=103/149 (69%) | YP_003575108.1 |
| LAB25g5 | - | 3781 | 4137 | 118 | putative membrane protein | 13117.78 | 8.50 | *Prevotella ruminicola 23* | E=3e-21; I=54/103 (52%); P=62/103 (60%) | YP_003575109.1 |
| LAB25g6 | - | 4141 | 4971 | 276 | MotA/TolQ/ExbB proton channel family protein | 29874.21 | 5.38 | *Prevotella ruminicola 23* | E=2e-92; I=171/253 (67%); P=191/253 (75%) | YP_003575110.1 |
| LAB25g7 | - | 5189 | 5971 | 260 | hydrolase, TatD family | 29506.72 | 4.76 | *Prevotella ruminicola 23* | E=2e-91; I=162/254 (63%); P=188/254 (74%) | YP_003575111.1 |
| LAB25g8 | - | 5968 | 6627 | 219 | TonB family protein | 24930.69 | 4.12 | *Prevotella ruminicola 23* | E=7e-63; I=120/199 (60%); P=151/199 (75%) | YP_003575113.1 |

Table S1 cont. Fosmid LAB 20

| Gene name | Strand | Left End | Right End | Length (AA) | Gene product name | MW (Da)* | pI* | Organism (best hit) | Expected Value (E), Identities (I), Positives (P) | Top homolog |
| --- | --- | --- | --- | --- | --- | --- | --- | --- | --- | --- |
| LAB20g1 | - | 3 | 200 | 66 | hypothetical protein BACPEC_03231 | 7412.18 | 4.82 | *Bacteroides pectinophilus ATCC 43243* | E=0.052; I=16/39 (41%); P=28/39 (71%) | ZP_03464130.1 |
| **LAB20g2** | **-** | **197** | **2134** | **645** | **putative glycosyl hydrolase** | **71900.07** | **5,86** | ***Prevotella ruminicola 23*** | **E=0.0; I=447/611 (73%); P=490/611 (80%)** | **YP_003575564.1** |
| LAB20g3 | + | 2178 | 3083 | 301 | transcriptional regulator | 34353.87 | 9,29 | *Prevotella sp. oral taxon 317 str. F0108* | E=3e-72; I=126/284 (44%); P=189/284 (66%) | ZP_06421662.1 |
| **LAB20g4** | **+** | **3090** | **5384** | **764** | **beta-glucosidase** | **82034.31** | **5,08** | ***uncultured rumen bacterium*** | **E=0.0; I=643/766 (83%); P=673/766 (87%)** | **ADD17009.1** |
| LAB20g5 | + | 5390 | 6157 | 255 | hydroxysteroid dehydrogenase 12 | 27868.77 | 10,39 | *Bacteroides sp. 2_1_33B* | E=3e-48; I=106/242 (43%); P=142/242 (58%) | ZP_06077382.1 |
| LAB20g6 | + | 6139 | 7092 | 317 | conserved hypothetical protein | 35116.06 | 8,06 | *Bacteroides xylanisolvens SD CC 1b* | E=2e-68; I=135/311 (43%); P=194/311 (62%) | ZP_06767206.1 |
| LAB20g7 | + | 7096 | 7866 | 256 | hypothetical protein ALIPUT_01817 | 29926.95 | 9,12 | *Alistipes putredinis DSM 17216* | E=1e-69; I=136/252 (53%); P=162/252 (64%) | ZP_02425667.1 |
| LAB20g8 | + | 7868 | 9100 | 410 | NADH flavin oxidoreductase | 45722.87 | 8,37 | *Bacteroides eggerthii 1_2_48FAA* | E=e-157; I=266/409 (65%); P=324/409 (79%) | EFV30009.1 |
| LAB20g9 | + | 9097 | 9912 | 271 | hypothetical protein BACCAC_03160 | 29443.72 | 8,97 | *Bacteroides caccae ATCC 43185* | E=6e-37; I=95/251 (37%); P=135/251 (53%) | ZP_01961528.1 |
| **LAB20g10** | **+** | **10041** | **12338** | **765** | **xylanase** | **85359.8** | **6,77** | ***uncultured microorganism*** | **E=0.0; I=410/459 (89%); P=425/459 (92%)** | **ADN44261.1** |
| LAB20g11 | + | 12454 | 14241 | 595 | ABC transporter related protein | 69014.53 | 5,38 | *Paludibacter propionicigenes WB4* | E=e-152; I=282/639 (44%); P=412/639 (64%) | YP_004043707.1 |
| LAB20g12 | - | 14280 | 14993 | 237 | hypothetical protein HMPREF0973_02029 | 26604.23 | 9,28 | *Prevotella veroralis F0319* | E=0.28; I=46/180 (25%); P=79/180 (43%) | ZP_05858039.1 |
| LAB20g13 | - | 15006 | 15452 | 148 | hypothetical protein HMPREF0665_02087 | 16642.26 | 8,22 | *Prevotella oris C735* | E=1e-09; I=41/125 (32%); P=67/125 (53%) | ZP_07035624.1 |
| LAB20g14 | - | 15449 | 15880 | 143 | hypothetical protein BACCOPRO_01312 | 15705.78 | 4,81 | *Bacteroides coprophilus DSM 18228* | E=6e-07; I=41/139 (29%); P=66/139 (47%) | ZP_03642952.1 |
| LAB20g15 | - | 15870 | 16499 | 209 | hypothetical protein BACCOPRO_01313 | 24489.21 | 8,05 | *Bacteroides coprophilus DSM 18228* | E=1e-21; I=70/163 (42%); P=98/163 (60%) | ZP_03642953.1 |
| LAB20g16 | + | 16667 | 17269 | 200 | hypothetical protein ALIPUT_02763 | 21559.76 | 4,90 | *Alistipes putredinis DSM 17216* | E=2e-24; I=68/201 (33%); P=116/201 (57%) | ZP_02426594.1 |
| LAB20g17 | + | 17280 | 17924 | 214 | Protein of unknown function (DUF2764). | 25029.18 | 4,43 | *Alistipes shahii WAL 8301* | E=6e-15; I=53/160 (33%); P=82/160 (51%) | CBK64734.1 |
| LAB20g18 | + | 17945 | 19687 | 580 | hypothetical protein ALIPUT_02765 | 64478.98 | 4,77 | *Alistipes putredinis DSM 17216* | E=0.0; I=409/580 (70%); P=478/580 (82%) | ZP_02426596.1 |
| LAB20g19 | + | 19692 | 21005 | 437 | hypothetical protein ALIPUT_02766 | 48170.78 | 4,62 | *Alistipes putredinis DSM 17216* | E=0.0; I=345/434 (79%); P=388/434 (89%) | ZP_02426597.1 |
| LAB20g20 | + | 21010 | 21621 | 203 | H(+)-transporting ATP synthase, vacuolar type, subunit D | 23351.77 | 9,33 | *Alistipes shahii WAL 8301* | E=9e-68; I=125/200 (62%); P=157/200 (78%) | CBK64736.1 |
| LAB20g21 | + | 21618 | 23405 | 595 | V-type ATPase 116kDa subunit family protein | 65714.58 | 5,50 | *Bacteroides sp. 4_1_36* | E=4e-89; I=210/610 (34%); P=306/610 (50%) | ZP_06202742.1 |
| LAB20g22 | + | 23419 | 23829 | 136 | ATP synthase subunit C | 13885.5 | 7,25 | *Bacteroides sp. 4_1_36* | E=4e-27; I=64/104 (61%); P=74/104 (71%) | ZP_02070389.1 |
| LAB20g23 | + | 23920 | 24582 | 220 | hypothetical protein HMPREF9720_2312 | 24550.57 | 8,54 | *Alistipes sp. HGB5* | E=0.36; I=38/147 (25%); P=68/147 (46%) | EFR58638.1 |
| LAB20g24 | + | 24584 | 25306 | 240 | conserved hypothetical protein | 26871.63 | 5,17 | *Clostridiales bacterium 1_7_47FAA* | E=2e-23; I=71/236 (30%); P=111/236 (47%) | ZP_04669361.1 |
| LAB20g25 | - | 25272 | 26321 | 349 | hypothetical protein BACDOR_01257 | 39339.17 | 6,25 | *Bacteroides dorei DSM 17855* | E=6e-74; I=136/320 (42%); P=205/320 (64%) | ZP_03299890.1 |
| LAB20g26 | - | 26430 | 26681 | 83 | ribosomal protein L31 | 9633.14 | 9,90 | *Bacteroidetes oral taxon 274 str. F0058* | E=1e-30; I=62/81 (76%); P=71/81 (87%) | ZP_06982030.1 |
| LAB20g27 | - | 26794 | 27861 | 355 | hypothetical protein LA_0544 | 40332.27 | 6,24 | *Leptospira interrogans serovar Lai str. 56601* | E=3e-30; I=103/383 (26%); P=166/383 (43%) | NP_710725.1 |
| LAB20g28 | - | 27869 | 29281 | 470 | Phosphomannomutase | 50534.76 | 4,79 | *Alistipes shahii WAL 8301* | E=e-167; I=296/469 (63%); P=360/469 (76%) | CBK63149.1 |
| LAB20g29 | + | 29372 | 29749 | 125 | conserved hypothetical protein | 14392.4 | 9,30 | *Prevotella ruminicola 23* | E=7e-42; I=80/123 (65%); P=97/123 (78%) | YP_003575311.1 |
| LAB20g30 | + | 29915 | 31156 | 413 | Arylsulfatase regulator (Fe-S oxidoreductase) | 47173 | 7,24 | *Alistipes shahii WAL 8301* | E=e-140; I=233/416 (56%); P=304/416 (73%) | CBK64060.1 |
| LAB20g31 | - | 31284 | 31793 | 169 | conserved hypothetical protein | 19182.18 | 5,34 | *Bacillus pumilus ATCC 7061* | E=8e-12; I=40/113 (35%); P=56/113 (49%) | ZP_03055818.1 |
| LAB20g32 | - | 32132 | 33097 | 321 | conserved hypothetical protein | 36686.04 | 8,29 | *Sulfurovum sp. NBC37-1* | E=2e-06; I=48/193 (24%); P=93/193 (48%) | YP_001358335.1 |
| LAB20g33 | - | 33094 | 33654 | 186 | alanine-glyoxylate/serine-pyruvate aminotransferase, aminotransferase class V | 21167.8 | 4,48 | *Cyanothece sp. ATCC 51142* | E=0.65; I=19/94 (20%); P=40/94 (42%) | YP_001804285.1 |
| LAB20g34 | + | 35142 | 36536 | 464 | MFS family major facilitator transporter, glycerol-3-phosphate:cation symporter | 51265.9 | 9,36 | *Prevotella bergensis DSM 17361* | E=0.0; I=323/464 (69%); P=361/464 (77%) | ZP_06006329.2 |
| LAB20g35 | + | 36640 | 37218 | 192 | GrpE protein | 21198.26 | 4,67 | *Spirosoma linguale DSM 74* | E=3e-37; I=74/133 (55%); P=102/133 (76%) | YP_003386369.1 |
| LAB20g36 | + | 37223 | 38386 | 387 | hypothetical protein BACCOPRO_03390 | 42457.91 | 8,46 | *Bacteroides coprophilus DSM 18228* | E=2e-88; I=172/390 (44%); P=233/390 (59%) | ZP_03644999.1 |
