# Supplementary material for: Microbial β-glucosidases from cow rumen metagenome enhance the saccharification of lignocellulose in combination with commercial cellulase cocktail
Source: Biotechnol Biofuels. 2012 Sep 21;5:73. doi: 10.1186/1754-6834-5-73 (PMC3477023; doi:10.1186/1754-6834-5-73)
Supplement: Additional file 2 — Table S2.Biochemical information of reported β-glucosidases. Data are based on bibliographic records that are specifically cited. [file 1754-6834-5-73-S2.doc]

**Additional file 2: Table S2 Biochemical information of reported beta-glucosidases.** Data are based on bibliographic records that are specifically cited.

| **Source** | **Origin** | **Substrates tested, kinetic parameters and pH and temperature optima^1^** | **Reference** |
| --- | --- | --- | --- |
| **Bacteria** | | | |
| *Oenococcus oeni* ST81 | Bacteria | K_m_: 0.38 mM (pNPβGlu)  V_max_: 5.21 nmolmin^-1^ (pNPβGlu)  Spec. act.: 297.4 mmolmin^-1^mg^-1^ (pNPβGlu)  Spec. act.: 23.6 mmolmin^-1^mg^-1^ (pNPβCel)  No activity reported for cellooligosaccharides  Opt. pH: 5.0  Opt Temp.: 40ºC | [Mesas JM](http://www.ncbi.nlm.nih.gov/pubmed?term=Mesas JM%5BAuthor%5D&cauthor=true&cauthor_uid=22748149), [Rodríguez MC](http://www.ncbi.nlm.nih.gov/pubmed?term=Rodríguez MC%5BAuthor%5D&cauthor=true&cauthor_uid=22748149), [Alegre MT](http://www.ncbi.nlm.nih.gov/pubmed?term=Alegre MT%5BAuthor%5D&cauthor=true&cauthor_uid=22748149): **Basic characterization and partial purification of β-glucosidase from cell-free extracts of *Oenococcus oeni* ST81.** *[Lett Appl Microbiol](http://www.ncbi.nlm.nih.gov/pubmed/22748149#%23)* 2012 doi: 10.1111/j.1472-765X.2012.03285.x. [Epub ahead of print] |
| *Lactobacillus casei* | Bacteria | K_m_: 0.07 mM (pNPβGlu)  V_max_: 71.4 nmolmin^-1^ (pNPβGlu)  Spec. act.: 73.5 μmolmin^-1^mg^-1^ (pNPβGlu)  No activity against cellobiose  Opt. pH: 5.5  Opt Temp.: 50ºC | Gueguen Y, Chemardin P, Labrot P, Arnaud A, Galzy P: **Purification and characterization of an intracellular b-glucosidase from a new strain of *Leuconostoc mesenteroides* isolated from cassava**. *J Appl Microbiol* 1997, **82:**469-476. |
| *Leuconostoc mesenteroides* | Bacteria | Spec. act.: 11 mmolmin^-1^mg^-1^ (pNPβGlu)  Spec. act.: 50 mmolmin^-1^mg^-1^ (cellobiose)  No activity against pNPβCel  Opt. pH: 6.3  Opt Temp.: 35ºC | Coulon S, Chemardin P, Arnaud A, Galzy P: **Purification and characterization of an intracellular b-glucosidase from *Lactobacillus casei* ATCC 393**. *Appl Biochem Biotechnol* 1998, **74:**105-114 |
| *Lactobacillus* *plantarum* | Bacteria | K_m_: 1.82 mM (pNPβGlu)  V_max_: 4.89 nmolmin^-1^ml^-1^ (pNPβGlu)  No activity reported for cellooligosaccharides  Opt. pH: 5.0 (stable between pH 4.5 and 7.5)  Opt Temp.: 45ºC | Sestelo ABF, Poza M, Villa TG: **b-glucosidase activity in a *Lactobacillus plantarum* wine strain**. *World J Microbiol Biotechnol* 2004, **20:**633–637. |
| *Lactobacillus brevis* | Bacteria | K_m_: 0.22 mM (pNPβGlu)  Spec. act.: 71 μmolmin^-1^mg^-1^ (pNPβGlu)  Spec. act.: 9.4 μmolmin^-1^mg^-1^ (pNPβCel)  No activity reported for cellooligosaccharides  Opt. pH: 6.0 (stable at pH 7.0 (t_1/2_ of 50 day) but not at pH 4.0 (t_1/2_ of 4 days).  Opt Temp.: 45ºC | [Michlmayr H](http://www.ncbi.nlm.nih.gov/pubmed?term=Michlmayr H%5BAuthor%5D&cauthor=true&cauthor_uid=19702863), [Schümann C](http://www.ncbi.nlm.nih.gov/pubmed?term=Schümann C%5BAuthor%5D&cauthor=true&cauthor_uid=19702863), [da Silva NM](http://www.ncbi.nlm.nih.gov/pubmed?term=da Silva NM%5BAuthor%5D&cauthor=true&cauthor_uid=19702863), [Kulbe KD](http://www.ncbi.nlm.nih.gov/pubmed?term=Kulbe KD%5BAuthor%5D&cauthor=true&cauthor_uid=19702863), [del Hierro AM](http://www.ncbi.nlm.nih.gov/pubmed?term=del Hierro AM%5BAuthor%5D&cauthor=true&cauthor_uid=19702863). **Isolation and basic characterization of a beta-glucosidase from a strain of *Lactobacillus brevis* isolated from a malolactic starter culture.** *[J Appl Microbiol](http://www.ncbi.nlm.nih.gov/pubmed/19702863#%23)* 2010, 108:550-559. |
| *Thermotoga petrophila* | Bacteria (hyperthermophilic) | K_m_: 2.8 mM (pNPβGlu)  V_max_: 42.7 mmolmin^-1^ (pNPβGlu)  *k*_cat_: 87400 s^-1^ (pNPβGlu)  *k*_cat_: 17500 s^-1^ (pNPβCel)  *k*_cat_/Km: 30800 s^-1^mM^-1^ (pNPβGlu)  *k*_cat_/Km: 4100 s^-1^mM^-1^ (pNPβCel)  Spec. act.: 30400 μmolmin^-1^mg^-1^ (pNPβGlu)  Spec. act.: 9400 μmolmin^-1^mg^-1^ (pNPβCel)  No activity against cellobiose and cellooligosaccharides  Opt. pH: 7.0-8.0  Opt Temp.: 80-90ºC | [Haq IU](http://www.ncbi.nlm.nih.gov/pubmed?term=Haq IU%5BAuthor%5D&cauthor=true&cauthor_uid=22714267), [Khan MA](http://www.ncbi.nlm.nih.gov/pubmed?term=Khan MA%5BAuthor%5D&cauthor=true&cauthor_uid=22714267), [Muneer B](http://www.ncbi.nlm.nih.gov/pubmed?term=Muneer B%5BAuthor%5D&cauthor=true&cauthor_uid=22714267), [Hussain Z](http://www.ncbi.nlm.nih.gov/pubmed?term=Hussain Z%5BAuthor%5D&cauthor=true&cauthor_uid=22714267), [Afzal S](http://www.ncbi.nlm.nih.gov/pubmed?term=Afzal S%5BAuthor%5D&cauthor=true&cauthor_uid=22714267), [Majeed S](http://www.ncbi.nlm.nih.gov/pubmed?term=Majeed S%5BAuthor%5D&cauthor=true&cauthor_uid=22714267), [Rashid N](http://www.ncbi.nlm.nih.gov/pubmed?term=Rashid N%5BAuthor%5D&cauthor=true&cauthor_uid=22714267), [Javed MM](http://www.ncbi.nlm.nih.gov/pubmed?term=Javed MM%5BAuthor%5D&cauthor=true&cauthor_uid=22714267), [Ahmad I](http://www.ncbi.nlm.nih.gov/pubmed?term=Ahmad I%5BAuthor%5D&cauthor=true&cauthor_uid=22714267): **Cloning, characterization and molecular docking of a highly thermostable β-1,4-glucosidase from *Thermotoga petrophila*.** *[Biotechnol Lett](http://www.ncbi.nlm.nih.gov/pubmed/22714267#%23)* 2012 [Epub ahead of print] |
| *Cellulomonas biazotea* | Bacteria | K_m_: 0.025 mM (pNPβGlu)  K_m_: 0.73 mM (cellobiose)  V_max_: 4.79 μmolmin^-1^mg^-1^ (pNPβGlu)  V_max_: 0.00033 mmolmin^-1^mg^-1^ (cellobiose)  Spec. act.: 133 μmolmin^-1^mg^-1^ (pNPβGlu)  *k*_cat_: - s^-1^ (pNPβGlu)  Opt. pH: 4.8  Opt Temp.: 70ºC | Lau ATY, Wong WKR: **Purification and characterization of a major secretory cellobiase, Cba2, from *Cellulomonas biazotea***. *Protein Expr Purif* 2001, **23:**159–166 |
| *Micrococcus antarcticus* | Bacteria (psychrotolerant) | K_m_: 7.0 mM (pNPβGlu)  *k*_cat_: 7.85 × 10^3^ s^-1^ (pNPβGlu)  Spec. act.: 385.54 μmolmin^-1^mg^-1^ (U/mg) (pNPβGlu)  Able to hydrolase cellobiose (396.6 U/mg), cellotriose (140.4 U/mg), cellotetraose (126.9 U/mg) and cellopentaose (98.7 U/mg)  Opt. pH: 6.5  Opt Temp.: 25ºC (t_1/2_ of 0.5 h at 30ºC) | [Fan HX](http://www.ncbi.nlm.nih.gov/pubmed?term=Fan HX%5BAuthor%5D&cauthor=true&cauthor_uid=22112277), [Miao LL](http://www.ncbi.nlm.nih.gov/pubmed?term=Miao LL%5BAuthor%5D&cauthor=true&cauthor_uid=22112277), [Liu Y](http://www.ncbi.nlm.nih.gov/pubmed?term=Liu Y%5BAuthor%5D&cauthor=true&cauthor_uid=22112277), [Liu HC](http://www.ncbi.nlm.nih.gov/pubmed?term=Liu HC%5BAuthor%5D&cauthor=true&cauthor_uid=22112277), [Liu ZP](http://www.ncbi.nlm.nih.gov/pubmed?term=Liu ZP%5BAuthor%5D&cauthor=true&cauthor_uid=22112277): **Gene cloning and characterization of a cold-adapted β-glucosidase belonging to glycosyl hydrolase family 1 from a psychrotolerant bacterium *Micrococcus antarcticus*.** *[Enzyme Microb Technol](http://www.ncbi.nlm.nih.gov/pubmed/22112277#%23)* 2011, **49:**94-99. |
| *Dictyoglomus turgidum* | Bacteia (thermophilic) | K_m_: 1.3 mM (pNPβGlu)  *k*_cat_: 13900 s^-1^ (pNPβGlu)  Spec. act.: 31.1 μmolmin^-1^mg^-1^ (U/mg) (pNPβGlu)  *k*_cat_/Km: 10400 s^-1^mM^-1^ (pNPβGlu)  No activity reported for cellooligosaccharides  Opt. pH: 5.0  Opt Temp.: 35-40ºC (t_1/2_ of 334 min at 85ºC) | [Kim YS](http://www.ncbi.nlm.nih.gov/pubmed?term=Kim YS%5BAuthor%5D&cauthor=true&cauthor_uid=21919440), [Yeom SJ](http://www.ncbi.nlm.nih.gov/pubmed?term=Yeom SJ%5BAuthor%5D&cauthor=true&cauthor_uid=21919440), [Oh DK](http://www.ncbi.nlm.nih.gov/pubmed?term=Oh DK%5BAuthor%5D&cauthor=true&cauthor_uid=21919440): **Characterization of a GH3 family β-glucosidase from *Dictyoglomus turgidum* and its application to the hydrolysis of isoflavone glycosides in spent coffee grounds.** *[J Agric Food Chem](http://www.ncbi.nlm.nih.gov/pubmed/21919440#%23)* 2011, **59:**11812-11818. |
| *Sphingomonas* sp. 2F2 | Bacteria | K_m_: 2.9 mM (pNPβGlu)  V_max_: 515.4 μmolmin^-1^mg^-1^ (pNPβGlu)  No activity reported for cellobiose and cellooligosaccharides  Opt. pH: 5.0  Opt Temp.: 37ºC | [Wang L](http://www.ncbi.nlm.nih.gov/pubmed?term=Wang L%5BAuthor%5D&cauthor=true&cauthor_uid=21906640), [Liu QM](http://www.ncbi.nlm.nih.gov/pubmed?term=Liu QM%5BAuthor%5D&cauthor=true&cauthor_uid=21906640), [Sung BH](http://www.ncbi.nlm.nih.gov/pubmed?term=Sung BH%5BAuthor%5D&cauthor=true&cauthor_uid=21906640), [An DS](http://www.ncbi.nlm.nih.gov/pubmed?term=An DS%5BAuthor%5D&cauthor=true&cauthor_uid=21906640), [Lee HG](http://www.ncbi.nlm.nih.gov/pubmed?term=Lee HG%5BAuthor%5D&cauthor=true&cauthor_uid=21906640), [Kim SG](http://www.ncbi.nlm.nih.gov/pubmed?term=Kim SG%5BAuthor%5D&cauthor=true&cauthor_uid=21906640), [Kim SC](http://www.ncbi.nlm.nih.gov/pubmed?term=Kim SC%5BAuthor%5D&cauthor=true&cauthor_uid=21906640), [Lee ST](http://www.ncbi.nlm.nih.gov/pubmed?term=Lee ST%5BAuthor%5D&cauthor=true&cauthor_uid=21906640), [Im WT](http://www.ncbi.nlm.nih.gov/pubmed?term=Im WT%5BAuthor%5D&cauthor=true&cauthor_uid=21906640): **Bioconversion of ginsenosides Rb(1), Rb(2), Rc and Rd by novel β-glucosidase hydrolyzing outer 3-O glycoside from *Sphingomonas* sp. 2F2: cloning, expression, and enzyme characterization*.*** *[J Biotechnol](http://www.ncbi.nlm.nih.gov/pubmed/21906640#%23)* 2011, 156:125-133. |
| Streptomyces matensis DIC-108 | Bacteria | K_m_: 1.60 mM (pNPβlaminaripentaose)  *k*_cat_: 8.1 s^-1^ (pNPβlaminaripentaose)  *k*_cat_/Km: 5063 s^-1^M^-1^(pNPβlaminaripentaose)  No activity reported for cellobiose and cellooligosaccharides  Opt. pH: 7.0 (stable up to 4 h)  Opt Temp.: 60ºC (rapidly lost its activity at temperature > 65°C) | [Shrestha KL](http://www.ncbi.nlm.nih.gov/pubmed?term=Shrestha KL%5BAuthor%5D&cauthor=true&cauthor_uid=21705773), [Liu SW](http://www.ncbi.nlm.nih.gov/pubmed?term=Liu SW%5BAuthor%5D&cauthor=true&cauthor_uid=21705773), [Huang CP](http://www.ncbi.nlm.nih.gov/pubmed?term=Huang CP%5BAuthor%5D&cauthor=true&cauthor_uid=21705773), [Wu HM](http://www.ncbi.nlm.nih.gov/pubmed?term=Wu HM%5BAuthor%5D&cauthor=true&cauthor_uid=21705773), [Wang WC](http://www.ncbi.nlm.nih.gov/pubmed?term=Wang WC%5BAuthor%5D&cauthor=true&cauthor_uid=21705773), [Li YK](http://www.ncbi.nlm.nih.gov/pubmed?term=Li YK%5BAuthor%5D&cauthor=true&cauthor_uid=21705773): **Characterization and identification of essential residues of the glycoside hydrolase family 64 laminaripentaose-producing-β-1, 3-glucanase.** *[Protein Eng Des Sel](http://www.ncbi.nlm.nih.gov/pubmed/21705773#%23)* 2011, **24:**617-625. |
| *Marinomonas* MWYL1 | Bacteria | K_m_: 0.91 mM (pNPβGlu)  V_max_: 555 μmolmin^-1^mg^-1^ (pNPβGlu)  *k*_cat_: 475.4 s^-1^ (pNPβGlu)  *k*_cat_/Km: 500.5 s^-1^mM^-1^ (pNPβGlu)  K_m_: 1.1 mM (cellobiose)  V_max_: 508 μmolmin^-1^mg^-1^ (cellobiose)  *k*_cat_: 435 s^-1^ (cellobiose)  *k*_cat_/Km: 395.8 s^-1^mM^-1^ (cellobiose)  Spec. act.: 396 μmolmin^-1^mg^-1^ (U/mg) (pNPβGlu)  Spec. act.: 367 μmolmin^-1^mg^-1^ (U/mg) (cellobiose)  No activity reported for cellooligosaccharides longer than cellobiose  Opt. pH: 7.0  Opt Temp.: 40ºC | [Zhao W](http://www.ncbi.nlm.nih.gov/pubmed?term=Zhao W%5BAuthor%5D&cauthor=true&cauthor_uid=21681424), [Peng R](http://www.ncbi.nlm.nih.gov/pubmed?term=Peng R%5BAuthor%5D&cauthor=true&cauthor_uid=21681424), [Xiong A](http://www.ncbi.nlm.nih.gov/pubmed?term=Xiong A%5BAuthor%5D&cauthor=true&cauthor_uid=21681424), [Fu X](http://www.ncbi.nlm.nih.gov/pubmed?term=Fu X%5BAuthor%5D&cauthor=true&cauthor_uid=21681424), [Tian Y](http://www.ncbi.nlm.nih.gov/pubmed?term=Tian Y%5BAuthor%5D&cauthor=true&cauthor_uid=21681424), [Yao Q](http://www.ncbi.nlm.nih.gov/pubmed?term=Yao Q%5BAuthor%5D&cauthor=true&cauthor_uid=21681424): **Expression and characterization of a cold-active and xylose-stimulated β-glucosidase from *Marinomonas* MWYL1 in *Escherichia coli*.** *[Mol Biol Rep](http://www.ncbi.nlm.nih.gov/pubmed/21681424#%23)* 2012, **39:**2937-2943. |
| *Thermotoga neapolitana* | Bacteria (thermophilic) | K_m_: 0.09 mM (pNPβGlu)  *k*_cat_: 89.6 s^-1^ (pNPβGlu)  *k*_cat_/Km: 996 s^-1^mM^-1^ (pNPβGlu)  Active against cellobiose (data not given); no information reported for cellooligosaccharides longer than cellobiose  Opt. pH: 5.6  Opt Temp.: 70ºC | [Pozzo T](http://www.ncbi.nlm.nih.gov/pubmed?term=Pozzo T%5BAuthor%5D&cauthor=true&cauthor_uid=20138890), [Pasten JL](http://www.ncbi.nlm.nih.gov/pubmed?term=Pasten JL%5BAuthor%5D&cauthor=true&cauthor_uid=20138890), [Karlsson EN](http://www.ncbi.nlm.nih.gov/pubmed?term=Karlsson EN%5BAuthor%5D&cauthor=true&cauthor_uid=20138890), [Logan DT](http://www.ncbi.nlm.nih.gov/pubmed?term=Logan DT%5BAuthor%5D&cauthor=true&cauthor_uid=20138890): Structural and functional analyses of beta-glucosidase 3B from *Thermotoga neapolitana*: a thermostable three-domain representative of glycoside hydrolase 3*.* ***[J Mol Biol](http://www.ncbi.nlm.nih.gov/pubmed?term=397%3A724-739 Pozzo" \l "%23)*** 2010, 397:724-739. |
| *Thermotoga maritima* | Bacteria (thermophilic) | K_m_: 0.0039 mM (pNPβGlu)  *k*_cat_: 6.4 s^-1^ (pNPβGlu)  *k*_cat_/Km: 1630 s^-1^mM^-1^ (pNPβGlu)  No activity reported for cellooligosaccharides  Opt. pH: 3.2-3.5  Opt Temp.: fully active and stable at 75ºC after 30 min incubation and only 75 and 45% activity  was lost at 80 and 85 ◦C, respectively | Kim BJ, Singh SP, Hayashi K: **Characteristics of chimeric enzymes constructed between *Thermotoga maritima* and *Agrobacterium tumefacien*s β-glucosidases: role of C-terminal domain in catalytic activity.** *Enzyme Microb Technol* 2006, **38:**952–959 |
| *Agrobacterium tumefacien* | Bacteria | K_m_: 0.01 mM (pNPβGlu)  *k*_cat_: 95.4 s^-1^ (pNPβGlu)  *k*_cat_/K_m_: 7950 s^-1^mM^-1^ (pNPβGlu)  No activity reported for cellooligosaccharides  Opt. pH: 6.5-8.0  Opt Temp.: 55ºC (stable) | Kim BJ, Singh SP, Hayashi K: **Characteristics of chimeric enzymes constructed between *Thermotoga maritima* and *Agrobacterium tumefacien*s β-glucosidases: role of C-terminal domain in catalytic activity.** *Enzyme Microb Technol* 2006, 38:952–959 |
| *Martelella mediterranea* | Bacteria (physhrophilic) | K_m_: 0.18 mg/ml (pNPβGlu)  V_max_: 196.08 μmolmin^-1^mg^-1^ (pNPβGlu)  Spec. act.: 196.1 μmolmin^-1^mg^-1^ (U/mg) (pNPβGlu)  Active against cellobiose too low (data not given); no information reported for cellooligosaccharides longer than cellobiose  Opt. pH: 11.0 (stable 24 h)  Opt Temp.: 45ºC (85% activity at 4ºC) | [Mao X](http://www.ncbi.nlm.nih.gov/pubmed?term=Mao X%5BAuthor%5D&cauthor=true&cauthor_uid=20503105), [Hong Y](http://www.ncbi.nlm.nih.gov/pubmed?term=Hong Y%5BAuthor%5D&cauthor=true&cauthor_uid=20503105), [Shao Z](http://www.ncbi.nlm.nih.gov/pubmed?term=Shao Z%5BAuthor%5D&cauthor=true&cauthor_uid=20503105), [Zhao Y](http://www.ncbi.nlm.nih.gov/pubmed?term=Zhao Y%5BAuthor%5D&cauthor=true&cauthor_uid=20503105), [Liu Z](http://www.ncbi.nlm.nih.gov/pubmed?term=Liu Z%5BAuthor%5D&cauthor=true&cauthor_uid=20503105): A novel cold-active and alkali-stable β-glucosidase gene isolated from the marine bacterium *Martelella mediterranea*. ***[Appl Biochem Biotechnol](http://www.ncbi.nlm.nih.gov/pubmed/20503105#%23)*** 2010, 162:2136-2148. |
| *Terrabacter ginsenosidimutans* sp. nov. | Bacteria | K_m_: 0.14-4.2 mM (pNPβGlu)  V_max_: 100.6-329 μmolmin^-1^mg^-1^ (pNPβGlu)  Spec. act.: 44.3 μmolmin^-1^mg^-1^ (U/mg) (pNPβGlu)  No activity reported for cellobiose and cellooligosaccharides  Opt. pH: 7.0  Opt Temp.: 45ºC (stable <37°C; 32% activity lost >45°C for 30 min) | [An DS](http://www.ncbi.nlm.nih.gov/pubmed?term=An DS%5BAuthor%5D&cauthor=true&cauthor_uid=20622122), [Cui CH](http://www.ncbi.nlm.nih.gov/pubmed?term=Cui CH%5BAuthor%5D&cauthor=true&cauthor_uid=20622122), [Lee HG](http://www.ncbi.nlm.nih.gov/pubmed?term=Lee HG%5BAuthor%5D&cauthor=true&cauthor_uid=20622122), [Wang L](http://www.ncbi.nlm.nih.gov/pubmed?term=Wang L%5BAuthor%5D&cauthor=true&cauthor_uid=20622122), [Kim SC](http://www.ncbi.nlm.nih.gov/pubmed?term=Kim SC%5BAuthor%5D&cauthor=true&cauthor_uid=20622122), [Lee ST](http://www.ncbi.nlm.nih.gov/pubmed?term=Lee ST%5BAuthor%5D&cauthor=true&cauthor_uid=20622122), [Jin F](http://www.ncbi.nlm.nih.gov/pubmed?term=Jin F%5BAuthor%5D&cauthor=true&cauthor_uid=20622122), [Yu H](http://www.ncbi.nlm.nih.gov/pubmed?term=Yu H%5BAuthor%5D&cauthor=true&cauthor_uid=20622122), [Chin YW](http://www.ncbi.nlm.nih.gov/pubmed?term=Chin YW%5BAuthor%5D&cauthor=true&cauthor_uid=20622122), [Lee HK](http://www.ncbi.nlm.nih.gov/pubmed?term=Lee HK%5BAuthor%5D&cauthor=true&cauthor_uid=20622122), [Im WT](http://www.ncbi.nlm.nih.gov/pubmed?term=Im WT%5BAuthor%5D&cauthor=true&cauthor_uid=20622122), [Kim SG](http://www.ncbi.nlm.nih.gov/pubmed?term=Kim SG%5BAuthor%5D&cauthor=true&cauthor_uid=20622122): **Identification and characterization of a novel *Terrabacter ginsenosidimutans* sp. nov. beta-glucosidase that transforms ginsenoside Rb1 into the rare gypenosides XVII and LXXV.** *[Appl Environ Microbiol](http://www.ncbi.nlm.nih.gov/pubmed/20622122#%23)* 2010, **76:**5827-5836. |
| *Bacillus halodurans* | Bacteria | K_m_: 4 mM (pNPβGlu)  *k*_cat_: 0.75 s^-1^ (pNPβGlu)  Spec. act.: 38.5 μmolmin^-1^mg^-1^ (U/mg) (pNPβGlu)  No activity reported for cellobiose and cellooligosaccharides  Opt. pH: 8.0  Opt Temp.: 45ºC | [Naz S](http://www.ncbi.nlm.nih.gov/pubmed?term=Naz S%5BAuthor%5D&cauthor=true&cauthor_uid=20618142), [Ikram N](http://www.ncbi.nlm.nih.gov/pubmed?term=Ikram N%5BAuthor%5D&cauthor=true&cauthor_uid=20618142), [Rajoka MI](http://www.ncbi.nlm.nih.gov/pubmed?term=Rajoka MI%5BAuthor%5D&cauthor=true&cauthor_uid=20618142), [Sadaf S](http://www.ncbi.nlm.nih.gov/pubmed?term=Sadaf S%5BAuthor%5D&cauthor=true&cauthor_uid=20618142), [Akhtar MW](http://www.ncbi.nlm.nih.gov/pubmed?term=Akhtar MW%5BAuthor%5D&cauthor=true&cauthor_uid=20618142): **Enhanced production and characterization of a beta-glucosidase from *Bacillus halodurans* expressed in *Escherichia coli*.** *[Biochemistry](http://www.ncbi.nlm.nih.gov/pubmed/20618142#%23)* (Mosc) 2010, 75:513-525. |
| *Stereum hirsutum* | Bacteria | *S. hirsutum* BGL showed the highest activity toward pNPβGlu.  K_m_: 0.54-2.11 mM (pNPβGlu)  V_max_: 3028 μmolmin^-1^mg^-1^ (pNPβGlu)  *k*_cat_: 4945 s^-1^ (pNPβGlu)  Spec. act.: 1730 μmolmin^-1^mg^-1^ (U/mg) (pNPβGlu)  Spec. act.: 112.5 μmolmin^-1^mg^-1^ (U/mg) (pNPβCel)  Spec. act.: 16.6 μmolmin^-1^mg^-1^ (U/mg) (cellobiose)  No activity for cellooligosaccharides longer than cellobiose  Opt. pH: 3.0-5.5  Opt Temp.: 65ºC (85% at 60C and 91% at 70ºC) | [Nguyen NP](http://www.ncbi.nlm.nih.gov/pubmed?term=Nguyen NP%5BAuthor%5D&cauthor=true&cauthor_uid=20532763), [Lee KM](http://www.ncbi.nlm.nih.gov/pubmed?term=Lee KM%5BAuthor%5D&cauthor=true&cauthor_uid=20532763), [Lee KM](http://www.ncbi.nlm.nih.gov/pubmed?term=Lee KM%5BAuthor%5D&cauthor=true&cauthor_uid=20532763), [Kim IW](http://www.ncbi.nlm.nih.gov/pubmed?term=Kim IW%5BAuthor%5D&cauthor=true&cauthor_uid=20532763), [Kim YS](http://www.ncbi.nlm.nih.gov/pubmed?term=Kim YS%5BAuthor%5D&cauthor=true&cauthor_uid=20532763), [Jeya M](http://www.ncbi.nlm.nih.gov/pubmed?term=Jeya M%5BAuthor%5D&cauthor=true&cauthor_uid=20532763), [Lee JK](http://www.ncbi.nlm.nih.gov/pubmed?term=Lee JK%5BAuthor%5D&cauthor=true&cauthor_uid=20532763): **One-step purification and characterization of a beta-1,4-glucosidase from a newly isolated strain of *Stereum hirsutum*.** *[Appl Microbiol Biotechnol](http://www.ncbi.nlm.nih.gov/pubmed/20532763#%23)* 2010, 87:2107-2116. |
| *Streptomyces* sp. S27 | Bacteria | K_m_: 1.89 mg/ml (laminarin)  Spec. act.: 236 μmolmin^-1^mg^-1^ (laminarin)  No activity reported for cellobiose and cellooligosaccharides  Opt. pH: 5.5 (retained full activity at 37°C at pH 5.0–9.0 for 1 h)  Opt Temp.: 65ºC (>80% activity retained at 60°C for 30 min, and <40% activity retained at 70°C and 80°C) | [Shi P](http://www.ncbi.nlm.nih.gov/pubmed?term=Shi P%5BAuthor%5D&cauthor=true&cauthor_uid=19697016), [Yao G](http://www.ncbi.nlm.nih.gov/pubmed?term=Yao G%5BAuthor%5D&cauthor=true&cauthor_uid=19697016), [Yang P](http://www.ncbi.nlm.nih.gov/pubmed?term=Yang P%5BAuthor%5D&cauthor=true&cauthor_uid=19697016), [Li N](http://www.ncbi.nlm.nih.gov/pubmed?term=Li N%5BAuthor%5D&cauthor=true&cauthor_uid=19697016), [Luo H](http://www.ncbi.nlm.nih.gov/pubmed?term=Luo H%5BAuthor%5D&cauthor=true&cauthor_uid=19697016), [Bai Y](http://www.ncbi.nlm.nih.gov/pubmed?term=Bai Y%5BAuthor%5D&cauthor=true&cauthor_uid=19697016), [Wang Y](http://www.ncbi.nlm.nih.gov/pubmed?term=Wang Y%5BAuthor%5D&cauthor=true&cauthor_uid=19697016), [Yao B](http://www.ncbi.nlm.nih.gov/pubmed?term=Yao B%5BAuthor%5D&cauthor=true&cauthor_uid=19697016): **Cloning, characterization, and antifungal activity of an endo-1,3-beta-D: -glucanase from *Streptomyces* sp. S27.** *[Appl Microbiol Biotechnol](http://www.ncbi.nlm.nih.gov/pubmed/19697016#%23)* 2010, **85:**1483-1490. |
| *Caldicellulosiruptor saccharolyticus* | Bacteria (thermophilic) | K_m_: 0.67 mM (pNPβGlu)  *k*_cat_: 387 s^-1^ (pNPβGlu)  *k*_cat_/Km: 579 s^-1^mM^-1^ (pNPβGlu)  Spec. act.: 13.0 μmolmin^-1^mg^-1^ (U/mg) (pNPβGlu)  Spec. act.: 1.3 μmolmin^-1^mg^-1^ (U/mg) (pNPβCel)  Spec. act.: 16.2 μmolmin^-1^mg^-1^ (U/mg) (cellobiose)  Spec. act.: 9.5 μmolmin^-1^mg^-1^ (U/mg) (cellotriose)  Spec. act.: 10.8 μmolmin^-1^mg^-1^ (U/mg) (cellotetraose)  Spec. act.: 6.8 μmolmin^-1^mg^-1^ (U/mg) (cellopentaose)  Opt. pH: 5.5  Opt Temp.: 70ºC (t_1/2_ of 250, 24.3 and 0.4 h at 60, 70 and 80ºC) | [Hong MR](http://www.ncbi.nlm.nih.gov/pubmed?term=Hong MR%5BAuthor%5D&cauthor=true&cauthor_uid=19577189), [Kim YS](http://www.ncbi.nlm.nih.gov/pubmed?term=Kim YS%5BAuthor%5D&cauthor=true&cauthor_uid=19577189), [Park CS](http://www.ncbi.nlm.nih.gov/pubmed?term=Park CS%5BAuthor%5D&cauthor=true&cauthor_uid=19577189), [Lee JK](http://www.ncbi.nlm.nih.gov/pubmed?term=Lee JK%5BAuthor%5D&cauthor=true&cauthor_uid=19577189), [Kim YS](http://www.ncbi.nlm.nih.gov/pubmed?term=Kim YS%5BAuthor%5D&cauthor=true&cauthor_uid=19577189), [Oh DK](http://www.ncbi.nlm.nih.gov/pubmed?term=Oh DK%5BAuthor%5D&cauthor=true&cauthor_uid=19577189): **Characterization of a recombinant beta-glucosidase from the thermophilic bacterium *Caldicellulosiruptor saccharolyticus*.** *[J Biosci Bioeng](http://www.ncbi.nlm.nih.gov/pubmed/19577189#%23)* 2009, **108:**36-40. |
| *Thermoplasma acidophilum* | Bacteria (thermophilic) | K_m_: 0.4 mM (pNPβGlu)  *k*_cat_: 141 s^-1^ (pNPβGlu)  *k*_cat_/Km: 381 s^-1^mM^-1^ (pNPβGlu)  Spec. act.: 83 μmolmin^-1^mg^-1^ (pNPβGlu)  No activity reported for cellobiose and cellooligosaccharides  Opt. pH: 6.0 (unstable <pH 6.0: at pH 6, t_1/2_ at 75ºC is 28 days but at pH 4.5 only 13 h)  Opt Temp.: 42ºC | [Kim HJ](http://www.ncbi.nlm.nih.gov/pubmed?term=Kim HJ%5BAuthor%5D&cauthor=true&cauthor_uid=19458917), [Park AR](http://www.ncbi.nlm.nih.gov/pubmed?term=Park AR%5BAuthor%5D&cauthor=true&cauthor_uid=19458917), [Lee JK](http://www.ncbi.nlm.nih.gov/pubmed?term=Lee JK%5BAuthor%5D&cauthor=true&cauthor_uid=19458917), [Oh DK](http://www.ncbi.nlm.nih.gov/pubmed?term=Oh DK%5BAuthor%5D&cauthor=true&cauthor_uid=19458917): **Characterization of an acid-labile, thermostable beta-glycosidase from *Thermoplasma acidophilum*.** *[Biotechnol Lett](http://www.ncbi.nlm.nih.gov/pubmed/19458917#%23)* 2009, **31:**1457-1462. |
| Archaea | | | |
| *Sulfolobus solfataricus* | Archaea | K_m_: 54 mM (pNPβGlu)  *k*_cat_: 4.9 s^-1^ (pNPβGlu)  *k*_cat_/Km: 0.09 s^-1^mM^-1^ (pNPβGlu)  No activity for pNPβCel and cellooligosaccharides  Opt. pH: 5.5  Opt Temp.: 65ºC | [**Cobucci-Ponzano B**](http://www.ncbi.nlm.nih.gov/pubmed?term=Cobucci-Ponzano B%5BAuthor%5D&cauthor=true&cauthor_uid=20427274), [**Aurilia V**](http://www.ncbi.nlm.nih.gov/pubmed?term=Aurilia V%5BAuthor%5D&cauthor=true&cauthor_uid=20427274), [**Riccio G**](http://www.ncbi.nlm.nih.gov/pubmed?term=Riccio G%5BAuthor%5D&cauthor=true&cauthor_uid=20427274), [**Henrissat B**](http://www.ncbi.nlm.nih.gov/pubmed?term=Henrissat B%5BAuthor%5D&cauthor=true&cauthor_uid=20427274), [**Coutinho PM**](http://www.ncbi.nlm.nih.gov/pubmed?term=Coutinho PM%5BAuthor%5D&cauthor=true&cauthor_uid=20427274), [**Strazzulli A**](http://www.ncbi.nlm.nih.gov/pubmed?term=Strazzulli A%5BAuthor%5D&cauthor=true&cauthor_uid=20427274), [**Padula A**](http://www.ncbi.nlm.nih.gov/pubmed?term=Padula A%5BAuthor%5D&cauthor=true&cauthor_uid=20427274), [**Corsaro MM**](http://www.ncbi.nlm.nih.gov/pubmed?term=Corsaro MM%5BAuthor%5D&cauthor=true&cauthor_uid=20427274), [**Pieretti G**](http://www.ncbi.nlm.nih.gov/pubmed?term=Pieretti G%5BAuthor%5D&cauthor=true&cauthor_uid=20427274), [**Pocsfalvi G**](http://www.ncbi.nlm.nih.gov/pubmed?term=Pocsfalvi G%5BAuthor%5D&cauthor=true&cauthor_uid=20427274), [**Fiume I**](http://www.ncbi.nlm.nih.gov/pubmed?term=Fiume I%5BAuthor%5D&cauthor=true&cauthor_uid=20427274), [**Cannio R**](http://www.ncbi.nlm.nih.gov/pubmed?term=Cannio R%5BAuthor%5D&cauthor=true&cauthor_uid=20427274), [**Rossi M**](http://www.ncbi.nlm.nih.gov/pubmed?term=Rossi M%5BAuthor%5D&cauthor=true&cauthor_uid=20427274), [**Moracci M**](http://www.ncbi.nlm.nih.gov/pubmed?term=Moracci M%5BAuthor%5D&cauthor=true&cauthor_uid=20427274): A new archaeal beta-glycosidase from *Sulfolobus solfataricus*: seeding a novel retaining beta-glycan-specific glycoside hydrolase family along with the human non-lysosomal glucosylceramidase GBA2. ***[J Biol Chem](http://www.ncbi.nlm.nih.gov/pubmed/20427274#%23)*** 2010, 285:20691-20703. |
| *Pyrococcus furiosus* | Archaea (hyperthermophilic) | K_m_: 2.8 mg/ml (laminarin)  Spec. act.: 1073 μmolmin^-1^mg^-1^ (laminarin)  K_m_: 4.7 mg/ml (lichenan)  Spec. act.: 85 μmolmin^-1^mg^-1^ (lichenan)  No activity reported for cellobiose and cellooligosaccharides  Opt. pH: 6.0-6.5 (stable from pH 5.0 to 7.0)  Opt Temp.: 100-105ºC (almost inactive at 40 °C and began to show significant activity above 60°C) | [Ippel JH](http://www.ncbi.nlm.nih.gov/pubmed?term=Ippel JH%5BAuthor%5D&cauthor=true&cauthor_uid=19913513), [Koutsopoulos S](http://www.ncbi.nlm.nih.gov/pubmed?term=Koutsopoulos S%5BAuthor%5D&cauthor=true&cauthor_uid=19913513), [Nabuurs SM](http://www.ncbi.nlm.nih.gov/pubmed?term=Nabuurs SM%5BAuthor%5D&cauthor=true&cauthor_uid=19913513), [van Berkel WJ](http://www.ncbi.nlm.nih.gov/pubmed?term=van Berkel WJ%5BAuthor%5D&cauthor=true&cauthor_uid=19913513), [van der Oost J](http://www.ncbi.nlm.nih.gov/pubmed?term=van der Oost J%5BAuthor%5D&cauthor=true&cauthor_uid=19913513), [van Mierlo CP](http://www.ncbi.nlm.nih.gov/pubmed?term=van Mierlo CP%5BAuthor%5D&cauthor=true&cauthor_uid=19913513): **NMR characterization of a 264-residue hyperthermostable endo-beta-1,3-glucanase.** *[Biochem Biophys Res Commun](http://www.ncbi.nlm.nih.gov/pubmed/19913513#%23)* 2010, **391:**370-375. |
| **Yeast** | | | |
| *Pichia pastoris* X33 | Yeast | K_m_: - mM (pNPβGlu)  Spec. act.: 101.7-103.5 units mg^-1^ (pNPβGlu)  Spec. act.: 58.4-64.1 μmolmin^-1^mg^-1^ (U/mg) (cellobiose)  Spec. act.: 38.6-41.4 μmolmin^-1^mg^-1^ (U/mg) (cellotriose)  Spec. act.: 32.2-35.5 μmolmin^-1^mg^-1^ (U/mg) (cellotetraose)  Spec. act.: 23.3-29.5 μmolmin^-1^mg^-1^ (U/mg) (cellopentaose)  Opt. pH: 6.0 (stable for a pH range of 4-7)  Opt Temp.: 60ºC (stable at 50-70°C) | [Liu D](http://www.ncbi.nlm.nih.gov/pubmed?term=Liu D%5BAuthor%5D&cauthor=true&cauthor_uid=22340848), [Zhang R](http://www.ncbi.nlm.nih.gov/pubmed?term=Zhang R%5BAuthor%5D&cauthor=true&cauthor_uid=22340848), [Yang X](http://www.ncbi.nlm.nih.gov/pubmed?term=Yang X%5BAuthor%5D&cauthor=true&cauthor_uid=22340848), [Zhang Z](http://www.ncbi.nlm.nih.gov/pubmed?term=Zhang Z%5BAuthor%5D&cauthor=true&cauthor_uid=22340848), [Song S](http://www.ncbi.nlm.nih.gov/pubmed?term=Song S%5BAuthor%5D&cauthor=true&cauthor_uid=22340848), [Miao Y](http://www.ncbi.nlm.nih.gov/pubmed?term=Miao Y%5BAuthor%5D&cauthor=true&cauthor_uid=22340848), [Shen Q](http://www.ncbi.nlm.nih.gov/pubmed?term=Shen Q%5BAuthor%5D&cauthor=true&cauthor_uid=22340848): **Characterization of a thermostable β-glucosidase from *Aspergillus fumigatus* Z5, and its functional expression in *Pichia pastoris* X33.** *[Microb Cell Fact](http://www.ncbi.nlm.nih.gov/pubmed/22340848#%23)* 2012, **11:**25. |
| *Debaryomyces vanrijiae* | Yeast | K_m_: 0.77 mM (pNPβGlu)  V_max_: 668 μmolmin^-1^mg^-1^ (pNPβGlu)  K_m_: 57.9 mM (cellobiose)  V_max_: 84.3 μmolmin^-1^mg^-1^ (cellobiose)  No activity for cellooligosaccharides longer than cellobiose  Opt. pH: 5.0  Opt Temp.: 40ºC | Belancic A, Gunata Z, Vallier MJ, Agosin E**: β-Glucosidase from the grape native yeast *Debaryomyces vanrijiae*: Purification, characterization, and its effect on monoterpene content of a muscat grape juice**. *J Agric Food Chem* 2003, **51:**1453-1459 |
| *Candida peltata* | Yeast | K_m_: 2.3 mM (pNPβGlu)  V_max_: 221 μmolmin^-1^mg^-1^ (pNPβGlu)  K_m_: 66 mM (cellobiose)  K_m_: 39 mM (cellotriose)  K_m_: 35 mM (cellotetraose)  K_m_: 21 mM (cellopentaose)  K_m_: 18 mM (cellohexaose)  Spec. act.: 108 units mg^-1^ (pNPβGlu)  V_max_: 75-5 μmolmin^-1^mg^-1^ (cellobiose-cellohexaose)  Opt. pH: 5.0  Opt Temp.: 50ºC | Galas E, Romanowska I: **Production purification and characterization of a highly glucose-tolerant novel ß-glucosidase from *Candida peltata***. *Appl Environ Microbiol* 1996, **62:**3165–3170 |
| Marine yeast *Williopsis saturnus* WC91-2 | Yeast | K_m_: 3.07 mg/ml (laminarin)  V_max_: 4.02 mgmin^-1^ml^-1^ (laminarin)  No activity reported for cellobiose and cellooligosaccharides  Opt. pH: 4.0  Opt Temp.: 40ºC | [Peng Y](http://www.ncbi.nlm.nih.gov/pubmed?term=Peng Y%5BAuthor%5D&cauthor=true&cauthor_uid=19513709), [Chi ZM](http://www.ncbi.nlm.nih.gov/pubmed?term=Chi ZM%5BAuthor%5D&cauthor=true&cauthor_uid=19513709), [Wang XH](http://www.ncbi.nlm.nih.gov/pubmed?term=Wang XH%5BAuthor%5D&cauthor=true&cauthor_uid=19513709), [Li J](http://www.ncbi.nlm.nih.gov/pubmed?term=Li J%5BAuthor%5D&cauthor=true&cauthor_uid=19513709): **Purification and molecular characterization of exo-beta-1,3-glucanases from the marine yeast *Williopsis saturnus* WC91-2.** *[Appl Microbiol Biotechnol](http://www.ncbi.nlm.nih.gov/pubmed/19513709#%23)* 2009, **85:**85-94. |
| *Monascus purpureus* | Yeast | K_m_: 0.39 mM (pNPβGlu)  V_max_: 6.51 μmolmin^-1^mg^-1^ (pNPβGlu)  K_m_: 2.86 mM (cellobiose)  V_max_: 4.71 μmolmin^-1^mg^-1^ (cellobiose)  Spec. act.: 84.4 units mg^-1^ (pNPβGlu)  Spec. act.: 18.5 units mg^-1^ (pNPβCel)  Spec. act.: 84 units mg^-1^ (cellobiose)  No activity for cellooligosaccharides longer than cellobiose  Opt. pH: 5.5  Opt Temp.: 50ºC | [Daroit DJ](http://www.ncbi.nlm.nih.gov/pubmed?term=Daroit DJ%5BAuthor%5D&cauthor=true&cauthor_uid=18633294), [Simonetti A](http://www.ncbi.nlm.nih.gov/pubmed?term=Simonetti A%5BAuthor%5D&cauthor=true&cauthor_uid=18633294), [Hertz PF](http://www.ncbi.nlm.nih.gov/pubmed?term=Hertz PF%5BAuthor%5D&cauthor=true&cauthor_uid=18633294), [Brandelli A](http://www.ncbi.nlm.nih.gov/pubmed?term=Brandelli A%5BAuthor%5D&cauthor=true&cauthor_uid=18633294): **Purification and characterization of an extracellular beta-glucosidase from *Monascus purpureus*.** *[J Microbiol Biotechnol](http://www.ncbi.nlm.nih.gov/pubmed/18633294#%23)* 2008, **18:**933-941. |
| *Metschnikowia pulcherrima* | Yeast | K_m_: 1.5 mM (pNPβGlu)  V_max_: 0.8 μmolmin^-1^mg^-1^ (pNPβGlu)  Spec. act.: 1.3 units mg^-1^ (pNPβGlu)  Spec. act.: 0.28 units mg^-1^ (cellobiose)  Opt. pH: 4.5  Opt Temp.: 50ºC | [González-Pombo P](http://www.ncbi.nlm.nih.gov/pubmed?term=González-Pombo P%5BAuthor%5D&cauthor=true&cauthor_uid=18414804), [Pérez G](http://www.ncbi.nlm.nih.gov/pubmed?term=Pérez G%5BAuthor%5D&cauthor=true&cauthor_uid=18414804), [Carrau F](http://www.ncbi.nlm.nih.gov/pubmed?term=Carrau F%5BAuthor%5D&cauthor=true&cauthor_uid=18414804), [Guisán JM](http://www.ncbi.nlm.nih.gov/pubmed?term=Guisán JM%5BAuthor%5D&cauthor=true&cauthor_uid=18414804), [Batista-Viera F](http://www.ncbi.nlm.nih.gov/pubmed?term=Batista-Viera F%5BAuthor%5D&cauthor=true&cauthor_uid=18414804), [Brena BM](http://www.ncbi.nlm.nih.gov/pubmed?term=Brena BM%5BAuthor%5D&cauthor=true&cauthor_uid=18414804): **One-step purification and characterization of an intracellular beta-glucosidase from *Metschnikowia pulcherrima*.** *[Biotechnol Lett](http://www.ncbi.nlm.nih.gov/pubmed/18414804#%23)* 2008, **30:**1469-1475. |
| **Fungi** | | | |
| *Phoma* sp. | Fungi | K_m_: 0.3 mM (pNPβGlu)  *k*_cat_: 0.5 s^-1^ (pNPβGlu)  *k*_cat_/Km: 1.6 s^-1^M^-1^ (pNPβGlu)  K_m_: 0.32 mM (cellobiose)  *k*_cat_: 699.4 s^-1^ (cellobiose)  *k*_cat_/Km: 215.8 × 10^5^ s^-1^M^-1^ (cellobiose)  Spec. act.: 84.5 units mg^-1^ (pNPβGlu)  Spec. act.: 3.2 units mg^-1^ (pNPβCel)  Spec. act.: 46.4 units mg^-1^ (cellobiose)  No activity for cellooligosaccharides longer than cellobiose  Opt. pH: 4.5  Opt Temp.: 60ºC (t_1/2_ of 53 h at 60ºC) | Choi JY, Ah-Reum P, Yong JK, Jae-Jin K, Chang-Jun C, Jeong-Jun Y: **Purification and characterization of an extracellular b-glucosidase produced by *Phoma* sp. KCTC11825BP isolated from rotten mandarin peel**. *J Microbiol Biotechnol* 2011, **21:**503–508 |
| *Rhizomucor miehei* | Fungi | K_m_: 0.12 mM (pNPβGlu)  Spec. act.: 468.2 μmolmin^-1^mg^-1^ (pNPβGlu)  Spec. act.: 54.3 μmolmin^-1^mg^-1^ (pNPβCel)  Spec. act.: 468.2 μmolmin^-1^mg^-1^ (cellobiose)  No activity for cellooligosaccharides longer than cellobiose  Opt. pH: 5.0-8.0  Opt Temp.: 65-70°C | [Krisch J](http://www.ncbi.nlm.nih.gov/pubmed?term=Krisch J%5BAuthor%5D&cauthor=true&cauthor_uid=22444635), [Bencsik O](http://www.ncbi.nlm.nih.gov/pubmed?term=Bencsik O%5BAuthor%5D&cauthor=true&cauthor_uid=22444635), [Papp T](http://www.ncbi.nlm.nih.gov/pubmed?term=Papp T%5BAuthor%5D&cauthor=true&cauthor_uid=22444635), [Vágvölgyi C](http://www.ncbi.nlm.nih.gov/pubmed?term=Vágvölgyi C%5BAuthor%5D&cauthor=true&cauthor_uid=22444635), [Takó M](http://www.ncbi.nlm.nih.gov/pubmed?term=Takó M%5BAuthor%5D&cauthor=true&cauthor_uid=22444635): **Characterization of a β-glucosidase with transgalactosylation capacity from the zygomycete *Rhizomucor miehei*.** *[Bioresour Technol](http://www.ncbi.nlm.nih.gov/pubmed/22444635#%23)* 2012, **114:**555-160 |
| *Stachybotrys* sp. | Fungi | K_m_: 1.85 mM (pNPβGlu)  V_max_: 211 μmolmin^-1^mg^-1^ (pNPβGlu)  *k*_cat_: 915 s^-1^ (pNPβGlu)  Hydrolase cellobiose to cellotetraose but it form trnasglycosylation products (no activity values given)  Opt. pH: 6.0  Opt Temp.: 50ºC | Saibi W, Amouri B, Gargouri A: **Purification and biochemical characterization of a transglucosilating β-glucosidase of *Stachybotrys* strain**. *Appl Microb Biotechnol* 2007, **77:**293–300 |
| *Stachybotrys* sp. | Fungi | K_m_: 0.30 mM (pNPβGlu)  V_max_: 78 μmolmin^-1^mg^-1^ (pNPβGlu)  *k*_cat_: 118 s^-1^ (pNPβGlu)  *k*_cat_/Km: 3.93 × 10^5^ s^-1^M^-1^  K_m_: 2.2 mM (cellobiose)  V_max_: 59.4 μmolmin^-1^mg^-1^ (cellobiose)  *k*_cat_: 118 s^-1^ (pNPβGlu)  No activity reported for cellooligosaccharides longer than cellobiose  Opt. pH: 6.0  Opt Temp.: 50ºC | Amouri B, Gargouri A: **Characterization of a novel β-glucosidase from a *Stachybotrys* strain**. *Biochem Eng J* 2006, **32:**191–197 |
| *Daldinia eschscholzii* | Fungi | K_m_: 1.52 mM (pNPβGlu)  V_max_: 3.20 μmolmin^-1^mg^-1^ (pNPβGlu)  *k*_cat_: 3.45 s^-1^ (pNPβGlu)  *k*_cat_/Km: 2.27 × 10^3^ s^-1^M^-1^  Spec. act.: 77.8 μmolmin^-1^mg^-1^ (pNPβGlu)  Spec. act.: 77.8 μmolmin^-1^mg^-1^ (cellobiose)  No activity for cellooligosaccharides longer than cellobiose  Opt. pH: 5.0 (stable at pH 5.0)  Opt Temp.: 50ºC (stable up to 50ºC) | Karnchanatat A, Petsom A, Sanvanich P, Piaphukiew J, Whalley AJ, Reynolds CD, et al: **Purification and biochemical characterization of an extracellular β-glucosidase from the wood-decaying fungus *Daldinia eschscholzii* (Ehrenb.:Fr.)Rehm**. *FEMS Microbiol Lett* 2007, **270:**162–170 |
| *Nectria catalinensis* | Fungi | K_m_: 0.25 mM (pNPβGlu)  V_max_: 0.22 μmolmin^-1^mg^-1^ (pNPβGlu)  *k*_cat_: 370 s^-1^ (pNPβGlu)  *k*_cat_/Km: 1.27 × 10^6^ s^-1^M^-1^  Opt. pH: 4.2-5.8  Opt Temp.: 50-55ºC | Pardo AG, Forchiassin F: **Influence of temperature and pH on cellulase activity and stability in *Nectria catalinensis***. *Rev Argent Microbiol* 1999, **31:**3–15 |
| *Scytalidium thermophilum* | Fungi | K_m_: 0.29 mM (pNPβGlu)  V_max_: 13.23μmolmin^-1^mg^-1^ (pNPβGlu)  *k*_cat_: 577 s^-1^ (pNPβGlu)  *k*_cat_/Km: 1.98 × 10^7^ s^-1^M^-1^  (pNPβGlu)  K_m_: 1.61 mM (cellobiose)  V_max_: 4.12 μmolmin^-1^mg^-1^ (cellobiose)  No activity reported for cellooligosaccharides longer than cellobiose  Opt. pH: 6.5  Opt Temp.: 60ºC (t_1/2_ of 20 h at 55ºC) | Zanoelo FF, Polizeli Md Mde L, Terenzi HF, Jorge JA: **β-Glucosidase activity from the thermophilic fungus *Scytalidium thermophilum* is stimulated by glucose and xylose**. *FEMS Microbiol Lett* 2004, **240:**137–143 |
| *Chaetomium thermophilum* | Fungi (thermophilic) | K_m_: 0.76 mM (pNPβGlu)  V_max_: 3390 μmolmin^-1^mg^-1^ (pNPβGlu)  *k*_cat_: 147 s^-1^ (pNPβGlu)  *k*_cat_/Km: 1.93 × 10^5^ s^-1^M^-1^  K_m_: 3.13 mM (cellobiose)  Able to hydrolase cellotriose and cellotetraose (not data given)  Opt. pH: 5.5  Opt Temp.: 65ºC (t_1/2_ of 2 h at 60ºC) | Venturi LL, Tereni HF, Furriel RPM, Jorge JA: **Extracellular β-d-glucosidase from *Chaetomium thermophilum* var. coprophilum: Production, purification and some biochemical properties**. *J Basic Microbiol* 2002, **42:**55–56 |
| *Penicillium funiculosum* NCL1 | Fungi | K_m_: 0.057 mM (pNPβGlu)  Spec. act.: 1.920 units mg^-1^ (pNPβGlu)  Spec. act.: 1796 μmolmin^-1^mg^-1^ (pNPβGlu)  Spec. act.: 1796 μmolmin^-1^mg^-1^ (cellobiose)  No activity for cellooligosaccharides longer than cellobiose  Opt. pH: 4.0-5.0  Opt Temp.: 60ºC (t_1/2_ of 1 h at 60ºC) | [Ramani G](http://www.ncbi.nlm.nih.gov/pubmed?term=Ramani G%5BAuthor%5D&cauthor=true&cauthor_uid=22415789), [Meera B](http://www.ncbi.nlm.nih.gov/pubmed?term=Meera B%5BAuthor%5D&cauthor=true&cauthor_uid=22415789), [Vanitha C](http://www.ncbi.nlm.nih.gov/pubmed?term=Vanitha C%5BAuthor%5D&cauthor=true&cauthor_uid=22415789), [Rao M](http://www.ncbi.nlm.nih.gov/pubmed?term=Rao M%5BAuthor%5D&cauthor=true&cauthor_uid=22415789), [Gunasekaran P](http://www.ncbi.nlm.nih.gov/pubmed?term=Gunasekaran P%5BAuthor%5D&cauthor=true&cauthor_uid=22415789): **Production, Purification, and Characterization of a β-Glucosidase of *Penicillium funiculosum* NCL1.** *[Appl Biochem Biotechnol](http://www.ncbi.nlm.nih.gov/pubmed/22415789#%23)* 2012, **167:**959-972. |
| *Penicillium purpurogenum* KJS506 | Fungi | K_m_: - mM (pNPβGlu)  V_max_: 934 μmolmin^-1^mg^-1^ (pNPβGlu)  Spec. act.: 875 units mg^-1^ (pNPβGlu)  Spec. act.: 102 units mg^-1^ (pNPβCel)  Spec. act.: 432 units mg^-1^ (cellobiose)  Spec. act.: 111 units mg^-1^ (cellotriose)  Spec. act.: 96 units mg^-1^ (cellotetraose)  Spec. act.: 68.4 units mg^-1^ (cellopentaose)  Opt. pH: 5.0  Opt Temp.: 65ºC (t_1/2_ of 10 h at 65ºC) | [Jeya M](http://www.ncbi.nlm.nih.gov/pubmed?term=Jeya M%5BAuthor%5D&cauthor=true&cauthor_uid=20043150), [Joo AR](http://www.ncbi.nlm.nih.gov/pubmed?term=Joo AR%5BAuthor%5D&cauthor=true&cauthor_uid=20043150), [Lee KM](http://www.ncbi.nlm.nih.gov/pubmed?term=Lee KM%5BAuthor%5D&cauthor=true&cauthor_uid=20043150), [Tiwari MK](http://www.ncbi.nlm.nih.gov/pubmed?term=Tiwari MK%5BAuthor%5D&cauthor=true&cauthor_uid=20043150), [Lee KM](http://www.ncbi.nlm.nih.gov/pubmed?term=Lee KM%5BAuthor%5D&cauthor=true&cauthor_uid=20043150), [Kim SH](http://www.ncbi.nlm.nih.gov/pubmed?term=Kim SH%5BAuthor%5D&cauthor=true&cauthor_uid=20043150), [Lee JK](http://www.ncbi.nlm.nih.gov/pubmed?term=Lee JK%5BAuthor%5D&cauthor=true&cauthor_uid=20043150): **Characterization of beta-glucosidase from a strain of *Penicillium purpurogenum* KJS506.** *[Appl Microbiol Biotechnol](http://www.ncbi.nlm.nih.gov/pubmed/20043150#%23)* 2010, **86:**1473-1484. |
| *Penicillium brasilianum* | Fungi | K_m_: 0.09 mM (pNPβGlu)  V_max_: 76 μmolmin^-1^mg^-1^ (pNPβGlu)  K_m_: 1.58 mM (cellobiose)  V_max_: 28 μmolmin^-1^mg^-1^ (cellobiose)  No activity reported for cellooligosaccharides longer than cellobiose  Opt. pH: 4.0-6.0  Opt Temp.: 60ºC (no loss of activity after 24 h; higher stability at these conditions in comparison to Novozym 188 and to other fungal beta-glucosidases) | [Krogh KB](http://www.ncbi.nlm.nih.gov/pubmed?term=Krogh KB%5BAuthor%5D&cauthor=true&cauthor_uid=19756584), [Harris PV](http://www.ncbi.nlm.nih.gov/pubmed?term=Harris PV%5BAuthor%5D&cauthor=true&cauthor_uid=19756584), [Olsen CL](http://www.ncbi.nlm.nih.gov/pubmed?term=Olsen CL%5BAuthor%5D&cauthor=true&cauthor_uid=19756584), [Johansen KS](http://www.ncbi.nlm.nih.gov/pubmed?term=Johansen KS%5BAuthor%5D&cauthor=true&cauthor_uid=19756584), [Hojer-Pedersen J](http://www.ncbi.nlm.nih.gov/pubmed?term=Hojer-Pedersen J%5BAuthor%5D&cauthor=true&cauthor_uid=19756584), [Borjesson J](http://www.ncbi.nlm.nih.gov/pubmed?term=Borjesson J%5BAuthor%5D&cauthor=true&cauthor_uid=19756584), [Olsson L](http://www.ncbi.nlm.nih.gov/pubmed?term=Olsson L%5BAuthor%5D&cauthor=true&cauthor_uid=19756584): **Characterization and kinetic analysis of a thermostable GH3 beta-glucosidase from *Penicillium brasilianum*.** *[Appl Microbiol Biotechnol](http://www.ncbi.nlm.nih.gov/pubmed/19756584#%23)* 2010, 86:143-154. |
| *Penicillium occitanis* | Fungi | K_m_: 0.37 mM (pNPβGlu)  V_max_: 0.55 μmolmin^-1^mg^-1^ (pNPβGlu)  K_m_: 1.43 mM (cellobiose)  V_max_: 0.9 μmolmin^-1^mg^-1^ (cellobiose)  No activity reported for cellooligosaccharides longer than cellobiose  Opt. pH: 4.5  Opt Temp.: 60ºC | [Bhiri F](http://www.ncbi.nlm.nih.gov/pubmed?term=Bhiri F%5BAuthor%5D&cauthor=true&cauthor_uid=18401747), [Chaabouni SE](http://www.ncbi.nlm.nih.gov/pubmed?term=Chaabouni SE%5BAuthor%5D&cauthor=true&cauthor_uid=18401747), [Limam F](http://www.ncbi.nlm.nih.gov/pubmed?term=Limam F%5BAuthor%5D&cauthor=true&cauthor_uid=18401747), [Ghrir R](http://www.ncbi.nlm.nih.gov/pubmed?term=Ghrir R%5BAuthor%5D&cauthor=true&cauthor_uid=18401747), [Marzouki N](http://www.ncbi.nlm.nih.gov/pubmed?term=Marzouki N%5BAuthor%5D&cauthor=true&cauthor_uid=18401747): **Purification and biochemical characterization of extracellular beta-glucosidases from the hypercellulolytic Pol6 mutant of *Penicillium occitanis*.** *[Appl Biochem Biotechnol](http://www.ncbi.nlm.nih.gov/pubmed/18401747#%23)* 2008, **149:**169-182. |
| *Penicillium funiculosum* | Fungi | K_m_: 0.35 mM (pNPβGlu)  V_max_: 2692.5 μmolmin^-1^mg^-1^ (pNPβGlu)  K_m_: 2.59 mM (cellobiose)  V_max_: 533.2 μmolmin^-1^mg^-1^ (cellobiose)  No activity reported for cellooligosaccharides longer than cellobiose  Opt. pH: 4.5 (stable in the pH range of 2.5-6.0)  Opt Temp.: 60ºC | [Karboune S](http://www.ncbi.nlm.nih.gov/pubmed?term=Karboune S%5BAuthor%5D&cauthor=true&cauthor_uid=18177005), [Geraert PA](http://www.ncbi.nlm.nih.gov/pubmed?term=Geraert PA%5BAuthor%5D&cauthor=true&cauthor_uid=18177005), [Kermasha S](http://www.ncbi.nlm.nih.gov/pubmed?term=Kermasha S%5BAuthor%5D&cauthor=true&cauthor_uid=18177005): **Characterization of selected cellulolytic activities of multi-enzymatic complex system from *Penicillium funiculosum*.** *[J Agric Food Chem](http://www.ncbi.nlm.nih.gov/pubmed/18177005#%23)* 2008, **56:**903-909. |
| *Fomitopsis palustris* | Fungi | K_m_: 0.706-0.971 mM (pNPβGlu)  Able to hydrolase from cellobiose to cellopentaose (9-37% librated glucose in 15 min assay; no specific activity data given)  Opt. pH: 2.5  Opt Temp.: 55ºC | [Okamoto K](http://www.ncbi.nlm.nih.gov/pubmed?term=Okamoto K%5BAuthor%5D&cauthor=true&cauthor_uid=22112950), [Sugita Y](http://www.ncbi.nlm.nih.gov/pubmed?term=Sugita Y%5BAuthor%5D&cauthor=true&cauthor_uid=22112950), [Nishikori N](http://www.ncbi.nlm.nih.gov/pubmed?term=Nishikori N%5BAuthor%5D&cauthor=true&cauthor_uid=22112950), [Nitta Y](http://www.ncbi.nlm.nih.gov/pubmed?term=Nitta Y%5BAuthor%5D&cauthor=true&cauthor_uid=22112950), [Yanase H](http://www.ncbi.nlm.nih.gov/pubmed?term=Yanase H%5BAuthor%5D&cauthor=true&cauthor_uid=22112950): **Characterization of two acidic β-glucosidases and ethanol fermentation in the brown rot fungus *Fomitopsis palustris*.** *[Enzyme Microb Technol](http://www.ncbi.nlm.nih.gov/pubmed/22112950#%23)* 2011, **48:**359-364. |
| *Neosartorya fischeri* NRRL181 | Fungi | K_m_: 68.0 mM (pNPβGlu)  V_max_: 886 μmolmin^-1^mg^-1^ (pNPβGlu)  Spec. act.: 710 units mg^-1^ (pNPβGlu)  Spec. act.: 2 units mg^-1^ (pNPβCel)  Spec. act.: 0.04 units mg^-1^ (cellobiose)  No activity for cellooligosaccharides longer than cellobiose  Opt. pH: 6.0  Opt Temp.: 40ºC | [Kalyani D](http://www.ncbi.nlm.nih.gov/pubmed?term=Kalyani D%5BAuthor%5D&cauthor=true&cauthor_uid=22042231), [Lee KM](http://www.ncbi.nlm.nih.gov/pubmed?term=Lee KM%5BAuthor%5D&cauthor=true&cauthor_uid=22042231), [Tiwari MK](http://www.ncbi.nlm.nih.gov/pubmed?term=Tiwari MK%5BAuthor%5D&cauthor=true&cauthor_uid=22042231), [Ramachandran P](http://www.ncbi.nlm.nih.gov/pubmed?term=Ramachandran P%5BAuthor%5D&cauthor=true&cauthor_uid=22042231), [Kim H](http://www.ncbi.nlm.nih.gov/pubmed?term=Kim H%5BAuthor%5D&cauthor=true&cauthor_uid=22042231), [Kim IW](http://www.ncbi.nlm.nih.gov/pubmed?term=Kim IW%5BAuthor%5D&cauthor=true&cauthor_uid=22042231), [Jeya M](http://www.ncbi.nlm.nih.gov/pubmed?term=Jeya M%5BAuthor%5D&cauthor=true&cauthor_uid=22042231), [Lee JK](http://www.ncbi.nlm.nih.gov/pubmed?term=Lee JK%5BAuthor%5D&cauthor=true&cauthor_uid=22042231): **Characterization of a recombinant aryl β-glucosidase from *Neosartorya fischeri* NRRL181.** *[Appl Microbiol Biotechnol](http://www.ncbi.nlm.nih.gov/pubmed/22042231#%23)* 2012, **94:**413-423. |
| *Ustilago esculenta* | Fungi | Spec. act.: 14 units mg^-1^ (pNPβGlu)  Spec. act.: 20 units mg^-1^ (cellobiose)  K_m_: 7.2 mM (cellobiose)  *k*_cat_: 78 s^-1^ (cellobiose)  *k*_cat_/Km: 11 s^-^1mM^-1^ (cellobiose)  Spec. act.: 14 units mg^-1^ (pNPβGlu)  Spec. act.: 20 units mg^-1^ (cellobiose)  No activity reported for cellooligosaccharides longer than cellobiose  Opt. pH: 5.0 (80% activity at pH 3.5–8.0)  Opt Temp.: 37ºC (80% activity at 4–40°C) | [Nakajima M](http://www.ncbi.nlm.nih.gov/pubmed?term=Nakajima M%5BAuthor%5D&cauthor=true&cauthor_uid=21850431), [Yamashita T](http://www.ncbi.nlm.nih.gov/pubmed?term=Yamashita T%5BAuthor%5D&cauthor=true&cauthor_uid=21850431), [Takahashi M](http://www.ncbi.nlm.nih.gov/pubmed?term=Takahashi M%5BAuthor%5D&cauthor=true&cauthor_uid=21850431), [Nakano Y](http://www.ncbi.nlm.nih.gov/pubmed?term=Nakano Y%5BAuthor%5D&cauthor=true&cauthor_uid=21850431), [Takeda T](http://www.ncbi.nlm.nih.gov/pubmed?term=Takeda T%5BAuthor%5D&cauthor=true&cauthor_uid=21850431): **Identification, cloning, and characterization of β-glucosidase from *Ustilago esculenta*.** *[Appl Microbiol Biotechnol](http://www.ncbi.nlm.nih.gov/pubmed/21850431#%23)* 2012, **93:**1989-1998. |
| *Daldinia eschscholzii* | Fungi | K_m_: 1.52 mM (pNPβGlu)  V_max_: 3.21 μmolmin^-1^mg^-1^ (pNPβGlu)  Spec. act.: 85 units mg^-1^ (pNPβGlu)  Spec. act.: 3.2 units mg^-1^ (pNPβCel)  Spec. act.: 47 units mg^-1^ (cellobiose)  No activity reported for cellooligosaccharides longer than cellobiose  Opt. pH: 5.0  Opt Temp.: 50ºC | Karnchanatat A, Petsom A, Sangvanich P, Piaphukiew J, Whalley JS, Reynolds CD, Sihanonth P: **Purification and biochemical characterization of an extracellular β-glucosidase from the wood-decaying fungus *Daldinia eschscholzii* (Ehrenb.:Fr.) Rehm**. *FEMS Microbiol Lett* 2007, **270:**162-170 |
| *Ceriporiopsis subvermispora* | Fungi | K_m_: 3.3 mM (pNPβGlu)  V_max_: 0.13 μmolmin^-1^mg^-1^ (pNPβGlu)  K_m_: 2.63 mM (pNPβGlu)  V_max_: 0.13 μmolmin^-1^mg^-1^ (pNPβGlu)  No activity reported for cellooligosaccharides longer than cellobiose  Opt. pH: 3.5  Opt Temp.: 60ºC | Magalhaes PO, Ferraz A, Milagres AF: **Enzymatic properties of two β-glucosidases from *Ceriporiopsis subvermispora* produced in biopulping conditions***. J Appl Microbiol* 2006, **101:**480–486 |
| *Melamocarpus sp.* | Fungi (thermophilic) | K_m_: 3.3 mM (pNPβGlu)  V_max_: 44 μmolmin^-1^mg^-1^ (pNPβGlu)  Spec. act.: 0.27 units mg^-1^ (pNPβCel)  No activity for cellooligosaccharides longer than cellobiose  Opt. pH: 6.0  Opt Temp.: 60ºC | Kaur J, Bhupinder SC, Badhan AK, Ghatora KS: **Purification and characterization of β-glucosidase from *Melanocarpu*s sp MTCC 3922**. *Electron J Biotechnol* 2007, **10:**261–270 |
| *Phanerochaete chrysosporium* | Fungi | K_m_: 1.0 mM (pNPβGlu)  V_max_: 33 μmolmin^-1^mg^-1^ (pNPβGlu)  No activity reported for cellooligosaccharides  Opt. pH: 4.0-5.2  Opt Temp.: - ºC | Lymar ES, Li B, Renganathan V: **Purification and characterization of a cellulose-binding (beta)-glucosidase from cellulose-degrading cultures of *Phanerochaete chrysosporium***. *Appl Environ Microbiol* 1995, **61:**2976–2980 |
| *Piptoporus betulinus* | Fungi | K_m_: 1.8mM (pNPβGlu)  V_max_: 19 μmolmin^-1^mg^-1^ (pNPβGlu)  Spec. act.: 53.1-19200 units μg^-1^ (pNPβGlu)  Spec. act.: 333-1460 units μg^-1^ (pNPβCel)  No activity reported for cellooligosaccharides  Opt. pH: 4.0  Opt Temp.: 60ºC | Valaskova V, Baldrian P: **Degradation of cellulose and hemicelluloses by the brown rot fungus *Piptoporus betulinus*-production of extracellular enzymes and characterization of the major cellulases**. *Microbiology* 2006, **152:**3613–3622 |
| *Xylaria regalis* | Fungi | K_m_: 1.7 mM (pNPβGlu)  V_max_: 326μmolmin^-1^mg^-1^ (pNPβGlu)  Spec. act.: 65.3 units mg^-1^ (pNPβGlu)  Spec. act.: 65 units mg^-1^ (cellobiose)  No activity reported for cellooligosaccharides longer than cellobiose  Opt. pH: 5.0  Opt Temp.: 50ºC | Wei DL, Kirimura K, Usami S, Lin TH: **Purification and characterization of an extracellular beta-glucosidase from the wood-grown fungus *Xylaria regalis*.** *Curr Microbiol* 1996, **33:**297–301 |
| *Termitomyces clypeatus* | Fungi | K_m_: 0.148-3.448 mM (pNPβGlu)  V_max_: 0.018-0.076 μmolmin^-1^mg^-1^ (pNPβGlu)  *k*_cat_: 0.022-0.52 Umg^-1^mM^-1^ (pNPβGlu)  Spec. act.: 0.54-17.9 units mg^-1^ (pNPβGlu)  Spec. act.: 0.148-21.4365 units mg^-1^ (cellobiose)  No activity reported for cellooligosaccharides longer than cellobiose  Opt. pH: activity 80-100% between pH ranges 5-8  Opt Temp.: 80% stability up to 60ºC | [Pal S](http://www.ncbi.nlm.nih.gov/pubmed?term=Pal S%5BAuthor%5D&cauthor=true&cauthor_uid=20031400), [Banik SP](http://www.ncbi.nlm.nih.gov/pubmed?term=Banik SP%5BAuthor%5D&cauthor=true&cauthor_uid=20031400), [Ghorai S](http://www.ncbi.nlm.nih.gov/pubmed?term=Ghorai S%5BAuthor%5D&cauthor=true&cauthor_uid=20031400), [Chowdhury S](http://www.ncbi.nlm.nih.gov/pubmed?term=Chowdhury S%5BAuthor%5D&cauthor=true&cauthor_uid=20031400), [Khowala S](http://www.ncbi.nlm.nih.gov/pubmed?term=Khowala S%5BAuthor%5D&cauthor=true&cauthor_uid=20031400): **Purification and characterization of a thermostable intra-cellular beta-glucosidase with transglycosylation properties from filamentous fungus *Termitomyces clypeatus*.** *[Bioresour Technol](http://www.ncbi.nlm.nih.gov/pubmed/20031400#%23)* 2010, **101:**2412-2420. |
| *Fomitopsis pinicola* | Fungi | K_m_: 1.76 mM (pNPβGlu)  V_max_: 1708 μmolmin^-1^mg^-1^ (pNPβGlu)  *k*_cat_: 2990 s^-1^ (pNPβGlu)  *k*_cat_/Km: 1700 s^-1^mM^-1^ (pNPβGlu)  Opt. pH: 4.5  Opt Temp.: 50ºC  Spec. act.: 1420 units mg^-1^ (pNPβGlu)  Spec. act.: 6.4 units mg^-1^ (pNPβCel)  Spec. act.: 117 units mg^-1^ (cellobiose)  Spec. act.: 16.1 units mg^-1^ (cellotetraose)  No activity for cellotriose and cellopentaose  Opt. pH: 4.5  Opt Temp.: 50ºC | [Joo AR](http://www.ncbi.nlm.nih.gov/pubmed?term=Joo AR%5BAuthor%5D&cauthor=true&cauthor_uid=19156406), [Jeya M](http://www.ncbi.nlm.nih.gov/pubmed?term=Jeya M%5BAuthor%5D&cauthor=true&cauthor_uid=19156406), [Lee KM](http://www.ncbi.nlm.nih.gov/pubmed?term=Lee KM%5BAuthor%5D&cauthor=true&cauthor_uid=19156406), [Sim WI](http://www.ncbi.nlm.nih.gov/pubmed?term=Sim WI%5BAuthor%5D&cauthor=true&cauthor_uid=19156406), [Kim JS](http://www.ncbi.nlm.nih.gov/pubmed?term=Kim JS%5BAuthor%5D&cauthor=true&cauthor_uid=19156406), [Kim IW](http://www.ncbi.nlm.nih.gov/pubmed?term=Kim IW%5BAuthor%5D&cauthor=true&cauthor_uid=19156406), [Kim YS](http://www.ncbi.nlm.nih.gov/pubmed?term=Kim YS%5BAuthor%5D&cauthor=true&cauthor_uid=19156406), [Oh DK](http://www.ncbi.nlm.nih.gov/pubmed?term=Oh DK%5BAuthor%5D&cauthor=true&cauthor_uid=19156406), [Gunasekaran P](http://www.ncbi.nlm.nih.gov/pubmed?term=Gunasekaran P%5BAuthor%5D&cauthor=true&cauthor_uid=19156406), [Lee JK](http://www.ncbi.nlm.nih.gov/pubmed?term=Lee JK%5BAuthor%5D&cauthor=true&cauthor_uid=19156406): **Purification and characterization of a beta-1,4-glucosidase from a newly isolated strain of *Fomitopsis* *pinicola*.** *[Appl Microbiol Biotechnol](http://www.ncbi.nlm.nih.gov/pubmed/19156406#%23)* 2009, **83:**285-294. |
| *Fomitopsis palustris* | Fungi | K_m_: 0.117 mM (pNPβGlu)  *k*_cat_: 721 s^-1^ (pNPβGlu)  K_m_: 4.81 mM (cellobiose)  *k*_cat_: 101.8 s^-1^ (cellobiose)  Spec. act.: 191 units mg^-1^ (pNPβGlu)  Spec. act.: 42.8 units mg^-1^ (pNPβCel)  Spec. act.: 191 units mg^-1^ (cellobiose)  No activity reported for cellooligosaccharides longer than cellobiose  Opt. pH: 4.5  Opt Temp.: 70ºC (t_1/2_ of 97 h at 55ºC amd 15 h at 65ºC) | [Yoon JJ](http://www.ncbi.nlm.nih.gov/pubmed?term=Yoon JJ%5BAuthor%5D&cauthor=true&cauthor_uid=18337693), [Kim KY](http://www.ncbi.nlm.nih.gov/pubmed?term=Kim KY%5BAuthor%5D&cauthor=true&cauthor_uid=18337693), [Cha CJ](http://www.ncbi.nlm.nih.gov/pubmed?term=Cha CJ%5BAuthor%5D&cauthor=true&cauthor_uid=18337693): **Purification and characterization of thermostable beta-glucosidase from the brown-rot basidiomycete *Fomitopsis palustris* grown on microcrystalline cellulose.** *[J Microbiol](http://www.ncbi.nlm.nih.gov/pubmed/18337693#%23)* 2008, 46:51-55. |
| *Paecilomyces thermophila* | Fungi (thermophilic) | K_m_: 0.27 mM (pNPβGlu)  V_max_: 780.3 μmolmin^-1^mg^-1^ (pNPβGlu)  *k*_cat_: 13 s^-1^ (pNPβGlu)  *k*_cat_/Km: 48.2 s^-1^mg^-1^ml (pNPβGlu)  K_m_: 0.65 mM (cellobiose)  V_max_: 272.1 μmolmin^-1^mg^-1^ (cellobiose)  *k*_cat_: 4.5 s^-1^ (cellobiose)  *k*_cat_/Km: 7.0 s^-1^mg^-1^ml (cellobiose)  Spec. act.: 97.2 units mg^-1^ (pNPβGlu)  Spec. act.: 49.1 units mg^-1^ (cellobiose)  Spec. act.: 43.3 units mg^-1^ (cellotriose)  Spec. act.: 31.2 units mg^-1^ (cellotetraose)  Spec. act.: 29.6 units mg^-1^ (cellopentaose)  Opt. pH: 6.2 (stable in the pH range of 2.5-6.0)  Opt Temp.: 75ºC (stable up to 65°C for 30 min and retained 57.9% of its activity at 70°C) | [Yang S](http://www.ncbi.nlm.nih.gov/pubmed?term=Yang S%5BAuthor%5D&cauthor=true&cauthor_uid=18092750), [Jiang Z](http://www.ncbi.nlm.nih.gov/pubmed?term=Jiang Z%5BAuthor%5D&cauthor=true&cauthor_uid=18092750), [Yan Q](http://www.ncbi.nlm.nih.gov/pubmed?term=Yan Q%5BAuthor%5D&cauthor=true&cauthor_uid=18092750), [Zhu H](http://www.ncbi.nlm.nih.gov/pubmed?term=Zhu H%5BAuthor%5D&cauthor=true&cauthor_uid=18092750): **Characterization of a thermostable extracellular beta-glucosidase with activities of exoglucanase and transglycosylation from *Paecilomyces thermophila*.** *[J Agric Food Chem](http://www.ncbi.nlm.nih.gov/pubmed/18092750#%23)* 2008, 56:602-608. |
| *Trichoderma citrinoviride* | Fungi | K_m_: 0.27 mM (pNPβGlu)  V_max_: 200 μmolmin^-1^mg^-1^ (pNPβGlu)  *k*_cat_: 8.953 s^-1^ (pNPβGlu)  *k*_cat_/Km: 3.19 × 10^7^ s^-1^M^-1^ (pNPβGlu)  Active against cellobiose (data not given); no information reported for cellooligosaccharides longer than cellobiose  Opt. pH: 5.0-5.5  Opt Temp.: 55°C | [Chandra M](http://www.ncbi.nlm.nih.gov/pubmed?term=Chandra M%5BAuthor%5D&cauthor=true&cauthor_uid=22438061), [Kalra A](http://www.ncbi.nlm.nih.gov/pubmed?term=Kalra A%5BAuthor%5D&cauthor=true&cauthor_uid=22438061), [Sangwan NS](http://www.ncbi.nlm.nih.gov/pubmed?term=Sangwan NS%5BAuthor%5D&cauthor=true&cauthor_uid=22438061), [Sangwan RS](http://www.ncbi.nlm.nih.gov/pubmed?term=Sangwan RS%5BAuthor%5D&cauthor=true&cauthor_uid=22438061): **Biochemical and proteomic characterization of a novel extracellular β-glucosidase from *Trichoderma citrinoviride*.** *[Mol Biotechnol](http://www.ncbi.nlm.nih.gov/pubmed/22438061#%23)* 2012, [Epub ahead of print] |
| *Trichoderma reesei* | Fungi | Spec. act.: 197 units mg^-1^ (pNPβGlu)  No activity reported for cellooligosaccharides  Opt. pH: 5.0  Opt Temp.: 70ºC | [Chen P](http://www.ncbi.nlm.nih.gov/pubmed?term=Chen P%5BAuthor%5D&cauthor=true&cauthor_uid=21826396), [Fu X](http://www.ncbi.nlm.nih.gov/pubmed?term=Fu X%5BAuthor%5D&cauthor=true&cauthor_uid=21826396), [Ng TB](http://www.ncbi.nlm.nih.gov/pubmed?term=Ng TB%5BAuthor%5D&cauthor=true&cauthor_uid=21826396), [Ye XY](http://www.ncbi.nlm.nih.gov/pubmed?term=Ye XY%5BAuthor%5D&cauthor=true&cauthor_uid=21826396): **Expression of a secretory β-glucosidase from *Trichoderma reesei* in *Pichia pastoris* and its characterization.** *[Biotechnol Lett](http://www.ncbi.nlm.nih.gov/pubmed/21826396#%23)* 2011, 33:2475-2479. |
| *Trichoderma koningii* AS3.2774 | Fungi | K_m_: 2.67 mM (pNPβGlu)  Spec. act.: 994.6 units mg^-1^ (pNPβGlu)  Active against cellobiose (data not given); no information reported for cellooligosaccharides longer than cellobiose  Opt. pH: 5.0  Opt Temp.: 50ºC | [Lin Y](http://www.ncbi.nlm.nih.gov/pubmed?term=Lin Y%5BAuthor%5D&cauthor=true&cauthor_uid=20691221), [Chen G](http://www.ncbi.nlm.nih.gov/pubmed?term=Chen G%5BAuthor%5D&cauthor=true&cauthor_uid=20691221), [Ling M](http://www.ncbi.nlm.nih.gov/pubmed?term=Ling M%5BAuthor%5D&cauthor=true&cauthor_uid=20691221), [Liang Z](http://www.ncbi.nlm.nih.gov/pubmed?term=Liang Z%5BAuthor%5D&cauthor=true&cauthor_uid=20691221): **A method of purification, identification and characterization of β-glucosidase from *Trichoderma koningii* AS3.2774.** *[J Microbiol Methods](http://www.ncbi.nlm.nih.gov/pubmed/20691221#%23)* 2010, **83:**74-81. |
| Cellobiase 250L (*A. niger* / Trichoderma reesei Rut C30) | Fungi | K_m_: 1.66 mM (cellobiose)  Active against cellobiose (data not given); no information reported for cellooligosaccharides longer than cellobiose  Opt. pH: -  Opt Temp.: 50ºC | [Chauve M](http://www.ncbi.nlm.nih.gov/pubmed?term=Chauve M%5BAuthor%5D&cauthor=true&cauthor_uid=20181208), [Mathis H](http://www.ncbi.nlm.nih.gov/pubmed?term=Mathis H%5BAuthor%5D&cauthor=true&cauthor_uid=20181208), [Huc D](http://www.ncbi.nlm.nih.gov/pubmed?term=Huc D%5BAuthor%5D&cauthor=true&cauthor_uid=20181208), [Casanave D](http://www.ncbi.nlm.nih.gov/pubmed?term=Casanave D%5BAuthor%5D&cauthor=true&cauthor_uid=20181208), [Monot F](http://www.ncbi.nlm.nih.gov/pubmed?term=Monot F%5BAuthor%5D&cauthor=true&cauthor_uid=20181208), [Lopes Ferreira N](http://www.ncbi.nlm.nih.gov/pubmed?term=Lopes Ferreira N%5BAuthor%5D&cauthor=true&cauthor_uid=20181208): **Comparative kinetic analysis of two fungal beta-glucosidases**. *[Biotechnol Biofuels](http://www.ncbi.nlm.nih.gov/pubmed/20181208#%23)* 2010, **3:**3; Grous W, Converse A, Grethlein H, Lynd L: **Kinetics of cellobiose hydrolysis using cellobiase composites from *Trichoderma reesei* and *Aspergillus niger***. *Biotechnol Bioeng* 1985, **27:**463–470 |
| Trichoderma reesei (Culture) | Fungi | K_m_: 0.182 mM (pNPβGlu)  K_m_: 2.1 mM (cellobiose)  *k*_cat_/Km: 2.45 × 10^4^ s^-1^M^-1^ (cellobiose)  *k*_cat_/Km: 7.93 × 10^5^ s^-1^M^-1^ (pNPβGlu)  Spec. act.: 88.5 μmolmin^-1^ml ^-1^ (pNPβGlu)  Spec. act.: 22.8 μmolmin^-1^ml ^-1^ (cellobiose)  No activity reported for cellooligosaccharides longer than cellobiose  Opt. pH: -  Opt Temp.: 50ºC | [Chauve M](http://www.ncbi.nlm.nih.gov/pubmed?term=Chauve M%5BAuthor%5D&cauthor=true&cauthor_uid=20181208), [Mathis H](http://www.ncbi.nlm.nih.gov/pubmed?term=Mathis H%5BAuthor%5D&cauthor=true&cauthor_uid=20181208), [Huc D](http://www.ncbi.nlm.nih.gov/pubmed?term=Huc D%5BAuthor%5D&cauthor=true&cauthor_uid=20181208), [Casanave D](http://www.ncbi.nlm.nih.gov/pubmed?term=Casanave D%5BAuthor%5D&cauthor=true&cauthor_uid=20181208), [Monot F](http://www.ncbi.nlm.nih.gov/pubmed?term=Monot F%5BAuthor%5D&cauthor=true&cauthor_uid=20181208), [Lopes Ferreira N](http://www.ncbi.nlm.nih.gov/pubmed?term=Lopes Ferreira N%5BAuthor%5D&cauthor=true&cauthor_uid=20181208): **Comparative kinetic analysis of two fungal beta-glucosidases**. *[Biotechnol Biofuels](http://www.ncbi.nlm.nih.gov/pubmed/20181208#%23)* 2010, **3:**3; Chen HZ, Hayn M, Esterbauer H: **Purification and characterization of 2 extracellular beta-glucosidases from *Trichoderma-reesei***. *Biochim Biophys Acta* 1992, **1121:**54–60 |
| Trichoderma reesei QM9414 | Fungi | K_m_: 0.102 mM (pNPβGlu)  K_m_: 1.25 mM (cellobiose)  Active against cellobiose (data not given); no information reported for cellooligosaccharides longer than cellobiose  Opt. pH: -  Opt Temp.: 540ºC | [Chauve M](http://www.ncbi.nlm.nih.gov/pubmed?term=Chauve M%5BAuthor%5D&cauthor=true&cauthor_uid=20181208), [Mathis H](http://www.ncbi.nlm.nih.gov/pubmed?term=Mathis H%5BAuthor%5D&cauthor=true&cauthor_uid=20181208), [Huc D](http://www.ncbi.nlm.nih.gov/pubmed?term=Huc D%5BAuthor%5D&cauthor=true&cauthor_uid=20181208), [Casanave D](http://www.ncbi.nlm.nih.gov/pubmed?term=Casanave D%5BAuthor%5D&cauthor=true&cauthor_uid=20181208), [Monot F](http://www.ncbi.nlm.nih.gov/pubmed?term=Monot F%5BAuthor%5D&cauthor=true&cauthor_uid=20181208), [Lopes Ferreira N](http://www.ncbi.nlm.nih.gov/pubmed?term=Lopes Ferreira N%5BAuthor%5D&cauthor=true&cauthor_uid=20181208): **Comparative kinetic analysis of two fungal beta-glucosidases**. *[Biotechnol Biofuels](http://www.ncbi.nlm.nih.gov/pubmed/20181208#%23)* 2010, **3:**3; Chirico WJ, Brown RD: **Purification and characterization of a beta-glucosidase from *Trichoderma reesei***. *Eur J Biochem* 1987, **165:**333–341 |
| *Trichoderma reesei* (NS 50013) | Fungi | Spec. act.: 5.0 μmolmin^-1^ml ^-1^ (pNPβGlu)  Spec. act.: 10.8 μmolmin^-1^ml ^-1^ (cellobiose)  No activity reported for cellooligosaccharides longer than cellobiose | Commercial cellulase (Novozymes AS) |
| *Aspergillus oryzae* | Fungi | K_m_: 0.29 mM (pNPβGlu)  *k*_cat_: 370 s^-1^ (pNPβGlu)  *k*_cat_/Km: 1.3 × 10^6^ s^-1^M^-1^ (pNPβGlu)  K_m_: 1.96 mM (cellobiose)  *k*_cat_: 1000 s^-1^ (cellobiose)  *k*_cat_/Km: 5.1 × 10^6^ s^-1^M^-1^ (cellobiose)  No activity reported for cellooligosaccharides longer than cellobiose  Opt. pH: 5.0  Opt Temp.: 60°C (t_1/2_ of 1 h at 65ºC) | Langston J, Sheehy N, Xu F: **Substrate specificity of *Aspergillus oryzae* family 3 β-glucosidase**. *Biochim Biophys Acta* 2006, **1764:**972–978 |
| *Aspergillus niger* | Fungi | K_m_: 1.0 mM (pNPβGlu)  *k*_cat_: 92 s^-1^ (pNPβGlu)  K_m_: 2.7 mM (cellobiose)  *k*_cat_: 103 s^-1^ (cellobiose)  K_m_: 0.5 mM (cellotriose)  *k*_cat_: 85 s^-1^ (cellotriose)  K_m_: 0.3 mM (cellotetraose)  *k*_cat_: 55 s^-1^ (cellotetraose)  K_m_: 0.5 mM (cellopentaose)  *k*_cat_: 52 s^-1^ (cellopentaose)  Opt. pH: -  Opt Temp.: - | Seidle H F, McKenzie K, Marten I, Shoseyov O, Huber RE: **Trp-262 is a key residue for the hydrolytic and transglucosidic reactivity of the *Aspergillus niger* family 3 β-glucosidase: Substitution results in enzymes with mainly transglucosidic activity**. *Arch Biochem Biophys* 2005, **444:**66–75 |
| *Aspergillus phoenicis* | Fungi | K_m_: 0.58 mM (pNPβGlu)  V_max_: - μmolmin^-1^mg^-1^ (pNPβGlu)  *k*_cat_: - s^-1^ (pNPβGlu)  No activity reported for cellooligosaccharides  Opt. pH: 5.0 (stable 4.0-8.0)  Opt Temp.: 60ºC (stable up to 50ºC) | Zhang C, Li D, Yu H, Zhang B, Jin F: **Purification and characterization of piceid-β-d-glucosidase from *Aspergillus oryzae***. *Process Biochemistry* 2007*,* **42:**83–88 |
| *Aspergillus niger* | Fungi | K_m_: 0.48 mM (pNPβGlu)  V_max_: - μmolmin^-1^mg^-1^ (pNPβGlu)  *k*_cat_: - s^-1^ (pNPβGlu)  No activity reported for cellooligosaccharides  Opt. pH: 4.5-5.0 (stable 4.0-8.0)  Opt Temp.: 60ºC (stable up to 60ºC) | Zhang C, Li D, Yu H, Zhang B, Jin F: **Purification and characterization of piceid-β-d-glucosidase from *Aspergillus oryzae***. *Process Biochemistry* 2007*,* **42:**83–88 |
| *Aspergillus oryzae* sp. *100* | Fungi | K_m_: 0.92 mM (pNPβGlu)  V_max_: - μmolmin^-1^mg^-1^ (pNPβGlu)  *k*_cat_: - s^-1^ (pNPβGlu)  No activity reported for cellooligosaccharides  Opt. pH: 5.0 (stable 4.0-5.0)  Opt Temp.: 60ºC (stable up to 60ºC) | Zhang C, Li D, Yu H, Zhang B, Jin F: **Purification and characterization of piceid-β-d-glucosidase from *Aspergillus oryzae*.** *Process Biochemistry* 2007*,* **42:**83–88 |
| *Aspergillus oryzae* | Fungi | K_m_: 0.55 mM (pNPβGlu)  K_m_: 7 mM (cellobiose)  V_max_: 353 μmolmin^-1^mg^-1^ (cellobiose)  Spec. act.: 1066 μmolmin^-1^mg ^-1^ (pNPβGlu)  Spec. act.: 938 μmolmin^-1^mg^-1^ (cellobiose)  Spec. act.: 800 μmolmin^-1^mg^-1^ (cellotriose)  Spec. act.: 661 μmolmin^-1^mg ^-1^ (cellotetraose)  Spec. act.: 512 μmolmin^-1^mg ^-1^ (cellopentaose) | Riou C, Salmon JM, Vallier MJ, Günata Z, Barre P: **Purification, characterization, and substrate specificity of a novel highly glucose-tolerant β-glucosidase from *Aspergillus oryza*e**. *Appl Environ Microbiol* 1998, **64:**3607-3614 |
| *Aspergillus niger* | Fungi | K_m_: 21.7 mM (pNPβGlu)  V_max_: 124.4 μmolmin^-1^mg^-1^ (pNPβGlu)  Spec. act.: 198.5 μmolmin^-1^mg ^-1^ (pNPβGlu)  Spec. act.: 5.27 μmolmin^-1^mg ^-1^ (cellobiose)  Spec. act.: 4.59 μmolmin^-1^mg ^-1^ (cellotriose)  Spec. act.: 3.24 μmolmin^-1^mg ^-1^ (cellotetraose)  Spec. act.: 2.30 μmolmin^-1^mg ^-1^ (cellopentaose)  Opt. pH: 5.0  Opt Temp.: 50ºC | Yan TR, Lin CL: **Purification and characterization of a glucose-tolerant β-glucosidase from *Aspergillus niger* CCRC 31494**. *Biosci Biotechnol Biochem* 1997, **61:**965-970 |
| *Aspergillus niger* | Fungi | K_m_: 1.1 mM (pNPβGlu)  V_max_: - μmolmin^-1^mg^-1^ (pNPβGlu)  Opt. pH: 4.6-5.3  Opt Temp.: 70ºC | Rashid MH, Siddiqui KS: **Purification and characterization of a beta-glucosidase from *Aspergillus niger***. *Folia Microbiol (Praha)* 1997, **42:**544–550 |
| Aspergillus niger (Culture) | Fungi | K_m_: 0.20-1.9 mM (pNPβGlu)  No activity reported for cellooligosaccharides  Opt. pH: 4.0-5.0  Opt Temp.: 60-65ºC | [Chauve M](http://www.ncbi.nlm.nih.gov/pubmed?term=Chauve M%5BAuthor%5D&cauthor=true&cauthor_uid=20181208), [Mathis H](http://www.ncbi.nlm.nih.gov/pubmed?term=Mathis H%5BAuthor%5D&cauthor=true&cauthor_uid=20181208), [Huc D](http://www.ncbi.nlm.nih.gov/pubmed?term=Huc D%5BAuthor%5D&cauthor=true&cauthor_uid=20181208), [Casanave D](http://www.ncbi.nlm.nih.gov/pubmed?term=Casanave D%5BAuthor%5D&cauthor=true&cauthor_uid=20181208), [Monot F](http://www.ncbi.nlm.nih.gov/pubmed?term=Monot F%5BAuthor%5D&cauthor=true&cauthor_uid=20181208), [Lopes Ferreira N](http://www.ncbi.nlm.nih.gov/pubmed?term=Lopes Ferreira N%5BAuthor%5D&cauthor=true&cauthor_uid=20181208): **Comparative kinetic analysis of two fungal beta-glucosidases**. *[Biotechnol Biofuels](http://www.ncbi.nlm.nih.gov/pubmed/20181208#%23)* 2010, **3:**3; Decker CH, Visser J, Schreier P: **beta-glucosidases from five black *Aspergillus species*: Study of their physico-chemical and biocatalytic properties.** *J Agric Food Chem*. 2000, **48:**4929–4936 |
| **Plant** | | | |
| Almond | Plant | K_m_: 2.24 mM (pNPβGlu)  V_max_: 588 μmolmin^-1^mg^-1^ (pNPβGlu)  *k*_cat_: - s^-1^ (pNPβGlu)  Opt. pH: 6.0  Opt Temp.: 60ºC | Yu HL, Xu JH, Lu WY, Lin GQ: **Identification, purification and characterization of β-glucosidase from apple seed as a novel catalyst for synthesis of *O*-glucosides**. *Enzyme Microb Technol* 2007, **40:**354–361; Romeu A, Montero MA, Ruiz JA, De Maria MJ, Jimenez JA, Rojas A, et al: **Effects of glucose, ethanol Hg(II) and Cu (II) on almond β-glucosidase**. *Biochem Mol Biol Int* 1994, **33:**939–946 |
| Apple seed | Plant | K_m_: 1.20 mM (pNPβGlu)  V_max_: 52.4 μmolmin^-1^mg^-1^ (pNPβGlu)  *k*_cat_: - s^-1^ (pNPβGlu)  Opt. pH: 6.0  Opt Temp.: 50ºC | Yu HL, Xu JH, Lu WY, Lin GQ: **Identification, purification and characterization of β-glucosidase from apple seed as a novel catalyst for synthesis of *O*-glucosides**. *Enzyme Microb Technol* 2007, **40:**354–361 |
| *Putranjiva roxburghii* seeds | Plant | K_m_: 0.53 mM (pNPβGlu)  V_max_: 0.181 μkatmg^-1^ (pNPβGlu)  *k*_cat_: 12 s^-1^ (pNPβGlu)  *k*_cat_/Km: 2.27 × 10^4^ s^-1^M^-1^  No activity reported for cellooligosaccharides  Opt. pH: 4.6  Opt Temp.: 65ºC (t_1/2_ of 40 min at 65ºC) | Patel GK, Kar B, Sharma AK: [**Characterization of a thermostable family 1 glycosyl hydrolase enzyme from *Putranjiva roxburghii* seeds.**](http://www.ncbi.nlm.nih.gov/pubmed/22086564) *[Appl Biochem Biotechnol](http://www.ncbi.nlm.nih.gov/pubmed/22086564#%23)* 2012, **166:**523-535. |
| *Arabidopsis thaliana* | Plant | K_m_: 1.9 mM (pNPβGlu)  *k*_cat_: 114 nkatmg^-1^ (pNPβGlu)  Opt. pH: 5.6 (stable from pH 5.0-8.5)  Opt Temp.: - | [Turan Y](http://www.ncbi.nlm.nih.gov/pubmed?term=Turan Y%5BAuthor%5D&cauthor=true&cauthor_uid=18774938): **A pseudo-beta-glucosidase in *Arabidopsis thaliana*: correction by site-directed mutagenesis, heterologous expression, purification, and characterization.** *[Biochemistry (Mosc)](http://www.ncbi.nlm.nih.gov/pubmed/18774938#%23)* 2008, **73:**912-919. |
| Corn stover | Plant | K_m_: 2.3 mM (pNPβGlu)  V_max_: 18.6 μmolmin^-1^mg^-1^ (pNPβGlu)  K_m_: 4.6 mM (cellobiose)  V_max_: 36.2 μmolmin^-1^mg^-1^ (cellobiose)  No information reported for cellooligosaccharides longer than cellobiose  Opt. pH: 4.8  Opt Temp.: 37ºC | [Han Y](http://www.ncbi.nlm.nih.gov/pubmed?term=Han Y%5BAuthor%5D&cauthor=true&cauthor_uid=18282703), [Chen H](http://www.ncbi.nlm.nih.gov/pubmed?term=Chen H%5BAuthor%5D&cauthor=true&cauthor_uid=18282703): **Characterization of beta-glucosidase from corn stover and its application in simultaneous saccharification and fermentation.** *[Bioresour Technol](http://www.ncbi.nlm.nih.gov/pubmed/18282703#%23)* 2008, **99:**6081-6087. |
| **Insect and mollusc** | | | |
| *Neotermes koshunensis* (in salivary glands of termite) | Insect (termite) | K_m_: 0.77 mM (pNPβGlu)  V_max_: 1.6 μmolmin^-1^mg^-1^ (pNPβGlu)  Spec. act.: 12.4 μmolmin^-1^ml ^-1^ (pNPβGlu)  Spec. act.: 10.3 μmolmin^-1^ml ^-1^ (cellobiose)  Spec. act.: 6.8 μmolmin^-1^ml ^-1^ (cellotriose)  Spec. act.: 5.9 μmolmin^-1^ml ^-1^ (cellotetraose)  Spec. act.: 5.6 μmolmin^-1^ml ^-1^ (cellopentaose)  Spec. act.: 5.3 μmolmin^-1^ml ^-1^ (cellohexaose)  Opt. pH: 5.0 (stable from pH 5.0 to 9.0)  Opt Temp.: 50ºC (80% activity up to 45°C and lost its activity at temperatures above 55°C) | [Uchima CA](http://www.ncbi.nlm.nih.gov/pubmed?term=Uchima CA%5BAuthor%5D&cauthor=true&cauthor_uid=21057947), [Tokuda G](http://www.ncbi.nlm.nih.gov/pubmed?term=Tokuda G%5BAuthor%5D&cauthor=true&cauthor_uid=21057947), [Watanabe H](http://www.ncbi.nlm.nih.gov/pubmed?term=Watanabe H%5BAuthor%5D&cauthor=true&cauthor_uid=21057947), [Kitamoto K](http://www.ncbi.nlm.nih.gov/pubmed?term=Kitamoto K%5BAuthor%5D&cauthor=true&cauthor_uid=21057947), [Arioka M](http://www.ncbi.nlm.nih.gov/pubmed?term=Arioka M%5BAuthor%5D&cauthor=true&cauthor_uid=21057947): **Heterologous expression and characterization of a glucose-stimulated β-glucosidase from the termite *Neotermes koshunensis* in *Aspergillus oryzae*.** *[Appl Microbiol Biotechnol](http://www.ncbi.nlm.nih.gov/pubmed/21057947#%23)* 2011, **89:**1761-1771. |
| Alkaline midgut of *Spodoptera frugiperda* larvae | Insect ([worm](http://en.wikipedia.org/wiki/Lepidoptera)) | K_m_: 0.12 % (laminarin)  *k*_cat_: 29 s^-1^ (laminarin)  *k*_cat_/Km: 241 s^-1^/% (laminarin)  No information reported for cellooligosaccharides  Opt. pH: 9.0  Opt Temp.: - | [Bragatto I](http://www.ncbi.nlm.nih.gov/pubmed?term=Bragatto I%5BAuthor%5D&cauthor=true&cauthor_uid=20816775), [Genta FA](http://www.ncbi.nlm.nih.gov/pubmed?term=Genta FA%5BAuthor%5D&cauthor=true&cauthor_uid=20816775), [Ribeiro AF](http://www.ncbi.nlm.nih.gov/pubmed?term=Ribeiro AF%5BAuthor%5D&cauthor=true&cauthor_uid=20816775), [Terra WR](http://www.ncbi.nlm.nih.gov/pubmed?term=Terra WR%5BAuthor%5D&cauthor=true&cauthor_uid=20816775), [Ferreira C](http://www.ncbi.nlm.nih.gov/pubmed?term=Ferreira C%5BAuthor%5D&cauthor=true&cauthor_uid=20816775): **Characterization of a β-1,3-glucanase active in the alkaline midgut of *Spodoptera frugiperda* larvae and its relation to β-glucan-binding proteins.** *[Insect Biochem Mol Biol](http://www.ncbi.nlm.nih.gov/pubmed/20816775#%23)* 2010, **40:**861-872. |
| *Tenebrio molitor* | Insect (beetle) | K_m_: 1.5 mg/ml (laminarin)  *k*_cat_: 1.4 s^-1^ (laminarin)  *k*_cat_/Km: 0.97 s^-1^(g/l)^-1^ (laminarin)  No information reported for cellooligosaccharides  Opt. pH: 4.7-7.9  Opt Temp.: - | Genta FA, Bragatto I, Terra WR, Ferreira C: **Purification, characterization and sequencing of the major beta-1,3-glucanase from the midgut of *Tenebrio molitor* larvae.** *Insect Biochem Mol Biol* 2009, **39:**861–874 |
| *Abracris flavolineata* midgut | Insect (langosta) | K_m_: -  V_max_: -  *k*_cat_: -  *k*_cat_/Km: 5.4 s^-1^M^-1^ (pNPβGlu)  K_m_: 0.19 % (laminarin)  *k*_cat_: 29 s^-1^ (laminarin)  *k*_cat_/Km: 153 s^-1^/% (laminarin)  No information reported for cellooligosaccharides  Opt. pH: stable in the pH range 3.9–9  Opt Temp.: - | Genta FA, Dumont AF, Marana SR, Terra WR, Ferreira C: **The interplay of processivity, substrate inhibition and a secondary substrate binding site of an insect exo-beta-1,3-glucanase.** *Biochim Biophys Acta* 2007, **1774:**1079–1091 |
| *Periplaneta Americana* (Lam) | Insect (cockroach) | K_m_: 0.074 % (laminarin)  *k*_cat_: 81 s^-1^ (laminarin)  *k*_cat_/Km: 1100 s^-1^/% (laminarin)  No information reported for cellooligosaccharides  Opt. pH: 6.5  Opt Temp.: - | Genta FA, Terra WR, Ferreira C: **Action pattern, specificity, lytic activities, and physiological role of five digestive beta-glucanases isolated from *Periplaneta americana*.** *Insect Biochem Mol Biol* 2003, **33:**1085–1097 |
| *Periplaneta Americana* (Liq 1) | Insect (cockroach) | K_m_: 0.16 % (laminarin)  *k*_cat_: 750 s^-1^ (laminarin)  *k*_cat_/Km: 4700 s^-1^/% (laminarin)  No information reported for cellooligosaccharides  Opt. pH: 6.0  Opt Temp.: - | Genta FA, Terra WR, Ferreira C: **Action pattern, specificity, lytic activities, and physiological role of five digestive beta-glucanases isolated from *Periplaneta americana*.** *Insect Biochem Mol Biol* 2003, **33:**1085–1097 |
| *Periplaneta Americana* (Liq 2) | Insect (cockroach) | K_m_: 0.36 % (laminarin)  *k*_cat_: 4.6 s^-1^ (laminarin)  *k*_cat_/Km: 12.8 s^-1^/% (laminarin)  No information reported for cellooligosaccharides  Opt. pH: 5.8  Opt Temp.: - | Genta FA, Terra WR, Ferreira C: **Action pattern, specificity, lytic activities, and physiological role of five digestive beta-glucanases isolated from *Periplaneta americana*.** *Insect Biochem Mol Biol* 2003, **33:**1085–1097 |
| Midgut of *Tenebrio molitor* larvae | Insect (beetle) | K_m_: 1.5 mg/ml (laminarin)  *k*_cat_: 1.40 s^-1^ (laminarin)  *k*_cat_/Km: 0.97 (g/l)-1/% (laminarin)  No information reported for cellooligosaccharides  Opt. pH: 5.8-6.6  Opt Temp.: - | [Genta FA](http://www.ncbi.nlm.nih.gov/pubmed?term=Genta FA%5BAuthor%5D&cauthor=true&cauthor_uid=19840850), [Bragatto I](http://www.ncbi.nlm.nih.gov/pubmed?term=Bragatto I%5BAuthor%5D&cauthor=true&cauthor_uid=19840850), [Terra WR](http://www.ncbi.nlm.nih.gov/pubmed?term=Terra WR%5BAuthor%5D&cauthor=true&cauthor_uid=19840850), [Ferreira C](http://www.ncbi.nlm.nih.gov/pubmed?term=Ferreira C%5BAuthor%5D&cauthor=true&cauthor_uid=19840850): Purification, characterization and sequencing of the major beta-1,3-glucanase from the midgut of *Tenebrio molitor* larvae. ***[Insect Biochem Mol Biol](http://www.ncbi.nlm.nih.gov/pubmed?term=The major beta-1%2C3-glucanase from Tenebrio molitor (TLam) was purified to homogeneity (yield%2C 6%25%3B enrichment%2C 113 fold%3B specific activity%2C " \l "%23)*** 2009, 39:861-874. |
| *Aplysia kurodai* | Mollusc (sea hare) | K_m_: 0.12-1.1 mg/ml (laminarin)  *k*_cat_: 0.05-23.1 s^-1^ (laminarin)  No information reported for cellooligosaccharides  Opt. pH: 5.7-6.0  Opt Temp.: 40-50ºC | [Kumagai Y](http://www.ncbi.nlm.nih.gov/pubmed?term=Kumagai Y%5BAuthor%5D&cauthor=true&cauthor_uid=19883786), [Ojima T](http://www.ncbi.nlm.nih.gov/pubmed?term=Ojima T%5BAuthor%5D&cauthor=true&cauthor_uid=19883786): **Isolation and characterization of two types of beta-1,3-glucanases from the common sea hare *Aplysia kurodai*.** *[Comp Biochem Physiol B Biochem Mol Bio.](http://www.ncbi.nlm.nih.gov/pubmed/19883786#%23)* 2010, 155:138-144. |
| **Unknown and ulcultured microorganisms** | | | |
| Uncultured microorganisms from yak rumen metagenome | Unknown and uncultured microorganism | K_m_: 1.06 mM (pNPβGlu)  *k*_cat_: 126.6 s^-1^ (pNPβGlu)  *k*_cat_/Km: 11900 s^-1^M^-1^  Spec. act.: 26-77 μmolmin^-1^ml ^-1^ (pNPβGlu)  Spec. act.: 5.3-33.5 μmolmin^-1^ml ^-1^ (cellobiose)  Able to hydrolase cellotriose and cellotetraose as revealed by thin layer chromatography (not data given)  Opt. pH: 4.6  Opt Temp.: 35- 40°C | [Bao L](http://www.ncbi.nlm.nih.gov/pubmed?term=Bao L%5BAuthor%5D&cauthor=true&cauthor_uid=22020745), [Huang Q](http://www.ncbi.nlm.nih.gov/pubmed?term=Huang Q%5BAuthor%5D&cauthor=true&cauthor_uid=22020745), [Chang L](http://www.ncbi.nlm.nih.gov/pubmed?term=Chang L%5BAuthor%5D&cauthor=true&cauthor_uid=22020745), [Sun Q](http://www.ncbi.nlm.nih.gov/pubmed?term=Sun Q%5BAuthor%5D&cauthor=true&cauthor_uid=22020745), [Zhou J](http://www.ncbi.nlm.nih.gov/pubmed?term=Zhou J%5BAuthor%5D&cauthor=true&cauthor_uid=22020745), [Lu H](http://www.ncbi.nlm.nih.gov/pubmed?term=Lu H%5BAuthor%5D&cauthor=true&cauthor_uid=22020745): **Cloning and characterization of two β-glucosidase/xylosidase enzymes from yak rumen metagenome.** *[Appl Biochem Biotechnol](http://www.ncbi.nlm.nih.gov/pubmed/22020745#%23)* 2012, **166:**72-86. |
| Uncultured bacteria from termite (*Reticulitermes santonensis*) gut | Unknown and uncultured microorganism | K_m_: 1.34 mM (pNPβGlu)  V_max_: 5.8 μmolL^-1^ (pNPβGlu)  Spec. act.: 0.441 units mg^-1^ (pNPβGlu)  Spec. act.: <0.02 units mg^-1^ (pNPβCel)  No information reported for cellooligosaccharides  Opt. pH: 6.0  Opt Temp.: 40ºC | [Mattéotti C](http://www.ncbi.nlm.nih.gov/pubmed?term=Mattéotti C%5BAuthor%5D&cauthor=true&cauthor_uid=21114521), [Haubruge E](http://www.ncbi.nlm.nih.gov/pubmed?term=Haubruge E%5BAuthor%5D&cauthor=true&cauthor_uid=21114521), [Thonart P](http://www.ncbi.nlm.nih.gov/pubmed?term=Thonart P%5BAuthor%5D&cauthor=true&cauthor_uid=21114521), [Francis F](http://www.ncbi.nlm.nih.gov/pubmed?term=Francis F%5BAuthor%5D&cauthor=true&cauthor_uid=21114521), [De Pauw E](http://www.ncbi.nlm.nih.gov/pubmed?term=De Pauw E%5BAuthor%5D&cauthor=true&cauthor_uid=21114521), [Portetelle D](http://www.ncbi.nlm.nih.gov/pubmed?term=Portetelle D%5BAuthor%5D&cauthor=true&cauthor_uid=21114521), [Vandenbol M](http://www.ncbi.nlm.nih.gov/pubmed?term=Vandenbol M%5BAuthor%5D&cauthor=true&cauthor_uid=21114521): **Characterization of a new β-glucosidase/β-xylosidase from the gut microbiota of the termite (*Reticulitermes santonensis*).** *[FEMS Microbiol Lett](http://www.ncbi.nlm.nih.gov/pubmed/21114521#%23)* 2011, **314:**147-157. |
| Uncultured microorganisms from soil metagenome | Unknown and uncultured microorganism | K_m_: 0.54-2.11 mM (pNPβGlu)  V_max_: 20.1-41.5 μmolmin^-1^mg^-1^ (pNPβGlu)  *k*_cat_: 804-1660 min^-1^ (pNPβGlu)  *k*_cat_/Km: 787-1489 min^-1^mM^-1^ (pNPβGlu)  Spec. act.: 10.8-12.6 units mg^-1^ (pNPβGlu)  Spec. act.: 5.6-8.5 units mg^-1^ (pNPβCel)  Opt. pH: 10.0  Opt Temp.: 25-30ºC | [Jiang C](http://www.ncbi.nlm.nih.gov/pubmed?term=Jiang C%5BAuthor%5D&cauthor=true&cauthor_uid=20971635), [Li SX](http://www.ncbi.nlm.nih.gov/pubmed?term=Li SX%5BAuthor%5D&cauthor=true&cauthor_uid=20971635), [Luo FF](http://www.ncbi.nlm.nih.gov/pubmed?term=Luo FF%5BAuthor%5D&cauthor=true&cauthor_uid=20971635), [Jin K](http://www.ncbi.nlm.nih.gov/pubmed?term=Jin K%5BAuthor%5D&cauthor=true&cauthor_uid=20971635), [Wang Q](http://www.ncbi.nlm.nih.gov/pubmed?term=Wang Q%5BAuthor%5D&cauthor=true&cauthor_uid=20971635), [Hao ZY](http://www.ncbi.nlm.nih.gov/pubmed?term=Hao ZY%5BAuthor%5D&cauthor=true&cauthor_uid=20971635), [Wu LL](http://www.ncbi.nlm.nih.gov/pubmed?term=Wu LL%5BAuthor%5D&cauthor=true&cauthor_uid=20971635), [Zhao GC](http://www.ncbi.nlm.nih.gov/pubmed?term=Zhao GC%5BAuthor%5D&cauthor=true&cauthor_uid=20971635), [Ma GF](http://www.ncbi.nlm.nih.gov/pubmed?term=Ma GF%5BAuthor%5D&cauthor=true&cauthor_uid=20971635), [Shen PH](http://www.ncbi.nlm.nih.gov/pubmed?term=Shen PH%5BAuthor%5D&cauthor=true&cauthor_uid=20971635), [Tang XL](http://www.ncbi.nlm.nih.gov/pubmed?term=Tang XL%5BAuthor%5D&cauthor=true&cauthor_uid=20971635), [Wu B](http://www.ncbi.nlm.nih.gov/pubmed?term=Wu B%5BAuthor%5D&cauthor=true&cauthor_uid=20971635): **Biochemical characterization of two novel β-glucosidase genes by metagenome expression cloning.** *[Bioresour Technol](http://www.ncbi.nlm.nih.gov/pubmed/20971635#%23)* 2011, **102:**3272-3278. |
| Uncultured microorganisms from marine metagenome | Unknown and uncultured microorganism | K_m_: 0.39 mM (pNPβGlu)  V_max_: 50.7 μmolmin^-1^mg^-1^ (pNPβGlu)  K_m_: 20.4 mM (cellobiose)  V_max_: 15.5 μmolmin^-1^mg^-1^ (cellobiose)  No information reported for cellooligosaccharides longer than cellobiose  Opt. pH: 6.0 (80% activity at pH 5.5-7.5)  Opt Temp.: 40ºC (85% activity from 35-45ºC) | [Fang Z](http://www.ncbi.nlm.nih.gov/pubmed?term=Fang Z%5BAuthor%5D&cauthor=true&cauthor_uid=20890102), [Fang W](http://www.ncbi.nlm.nih.gov/pubmed?term=Fang W%5BAuthor%5D&cauthor=true&cauthor_uid=20890102), [Liu J](http://www.ncbi.nlm.nih.gov/pubmed?term=Liu J%5BAuthor%5D&cauthor=true&cauthor_uid=20890102), [Hong Y](http://www.ncbi.nlm.nih.gov/pubmed?term=Hong Y%5BAuthor%5D&cauthor=true&cauthor_uid=20890102), [Peng H](http://www.ncbi.nlm.nih.gov/pubmed?term=Peng H%5BAuthor%5D&cauthor=true&cauthor_uid=20890102), [Zhang X](http://www.ncbi.nlm.nih.gov/pubmed?term=Zhang X%5BAuthor%5D&cauthor=true&cauthor_uid=20890102), [Sun B](http://www.ncbi.nlm.nih.gov/pubmed?term=Sun B%5BAuthor%5D&cauthor=true&cauthor_uid=20890102), [Xiao Y](http://www.ncbi.nlm.nih.gov/pubmed?term=Xiao Y%5BAuthor%5D&cauthor=true&cauthor_uid=20890102): **Cloning and characterization of a beta-glucosidase from marine microbial metagenome with excellent glucose tolerance.** *[J Microbiol Biotechnol](http://www.ncbi.nlm.nih.gov/pubmed/20890102#%23)* 2010, **20:**1351-1358. |
| Uncultured microorganisms from bioreactor sludge metagenome | Unknown and uncultured microorganism | K_m_: 1.45 mM (pNPβGlu)  V_max_: 20.5 μmolmin^-1^mg^-1^ (pNPβGlu)  *k*_cat_: 1370 min^-1^ (pNPβGlu)  *k*_cat_/Km: 943 min^-1^mM^-1^ (pNPβGlu)  Able to hydrolase cellobiose (data not given); no information reported for cellooligosaccharides longer than cellobiose  Opt. pH: 4.5  Opt Temp.: 37ºC (65% activity at <30ºC and >70% from 32-55ºC) | Jiang C, Hao ZY, Jin K, Li SX, Che ZQ, Ma GF, Wu B: Identification of a metagenome-derived β-glucosidase from bioreactor contents. *J Mol Catal B Enzym* 2010, 63:11–16 |
| Uncultured microorganisms from soil metagenome | Unknown and uncultured microorganism | K_m_: 0.19 mM (pNPβGlu)  V_max_: 4.75 μmolmin^-1^mg^-1^ (pNPβGlu)  *k*_cat_: 316.7 min^-1^ (pNPβGlu)  Spec. act.: 3.36 μmolmin^-1^μmol ^-1^ (pNPβGlu)  Spec. act.: 1.14 μmolmin^-1^μmol ^-1^ (pNPβCel)  No information reported for cellooligosaccharides  Opt. pH: 8.0  Opt Temp.: 42ºC | [Jiang C](http://www.ncbi.nlm.nih.gov/pubmed?term=Jiang C%5BAuthor%5D&cauthor=true&cauthor_uid=19851726), [Ma G](http://www.ncbi.nlm.nih.gov/pubmed?term=Ma G%5BAuthor%5D&cauthor=true&cauthor_uid=19851726), [Li S](http://www.ncbi.nlm.nih.gov/pubmed?term=Li S%5BAuthor%5D&cauthor=true&cauthor_uid=19851726), [Hu T](http://www.ncbi.nlm.nih.gov/pubmed?term=Hu T%5BAuthor%5D&cauthor=true&cauthor_uid=19851726), [Che Z](http://www.ncbi.nlm.nih.gov/pubmed?term=Che Z%5BAuthor%5D&cauthor=true&cauthor_uid=19851726), [Shen P](http://www.ncbi.nlm.nih.gov/pubmed?term=Shen P%5BAuthor%5D&cauthor=true&cauthor_uid=19851726), [Yan B](http://www.ncbi.nlm.nih.gov/pubmed?term=Yan B%5BAuthor%5D&cauthor=true&cauthor_uid=19851726), [Wu B](http://www.ncbi.nlm.nih.gov/pubmed?term=Wu B%5BAuthor%5D&cauthor=true&cauthor_uid=19851726). **Characterization of a novel beta-glucosidase-like activity from a soil metagenome.** *[J Microbiol](http://www.ncbi.nlm.nih.gov/pubmed/19851726#%23)* 2009, **47:**542-548. |
| Digestive fluid of larvae of the palm weevil, *Rhynchophorus palmarum* | Unknown and uncultured microorganism | K_m_: 0.25 mM (pNPβGlu)  *k*_cat_: 60.12 s^-1^ (pNPβGlu)  *k*_cat_/Km: 240.48 s^-1^mM^-1^ (pNPβGlu)  K_m_: 0.31 mM (cellobiose)  *k*_cat_: 41.79 s^-1^ (cellobiose)  *k*_cat_/Km: 134.8 s^-1^mM^-1^ (cellobiose)  Spec. act.: 25.1 μmolmin^-1^μmol ^-1^ (cellobiose)  Spec. act.: 32.6 μmolmin^-1^μmol ^-1^ (cellotriose)  Spec. act.: 30.1 μmolmin^-1^μmol ^-1^ (cellotetraose)  Spec. act.: 22.8 μmolmin^-1^μmol ^-1^ (cellopentaose)  Opt. pH: 5.0 (stable from pH 5.0-6.0)  Opt Temp.: 55ºC | [Yapi Assoi Yapi D](http://www.ncbi.nlm.nih.gov/pubmed?term=Yapi Assoi Yapi D%5BAuthor%5D&cauthor=true&cauthor_uid=19611239), [Gnakri D](http://www.ncbi.nlm.nih.gov/pubmed?term=Gnakri D%5BAuthor%5D&cauthor=true&cauthor_uid=19611239), [Lamine Niamke S](http://www.ncbi.nlm.nih.gov/pubmed?term=Lamine Niamke S%5BAuthor%5D&cauthor=true&cauthor_uid=19611239), [Patrice Kouame L](http://www.ncbi.nlm.nih.gov/pubmed?term=Patrice Kouame L%5BAuthor%5D&cauthor=true&cauthor_uid=19611239): **Purification and biochemical characterization of a specific beta-glucosidase from the digestive fluid of larvae of the palm weevil, *Rhynchophorus palmarum*.** *[J Insect Sci](http://www.ncbi.nlm.nih.gov/pubmed/19611239#%23)* 2009, **9:**4. |
| Uncultured microorganisms from buffalo rumen metagenome | Unknown and uncultured microorganism | Spec. act.: 22.8 μmolmin^-1^mg^-1^ (pNPβGlu)  Opt. pH: 6.0-6.5  Opt Temp.: 45ºC | [Guo H](http://www.ncbi.nlm.nih.gov/pubmed?term=Guo H%5BAuthor%5D&cauthor=true&cauthor_uid=18464606), [Feng Y](http://www.ncbi.nlm.nih.gov/pubmed?term=Feng Y%5BAuthor%5D&cauthor=true&cauthor_uid=18464606), [Mo X](http://www.ncbi.nlm.nih.gov/pubmed?term=Mo X%5BAuthor%5D&cauthor=true&cauthor_uid=18464606), [Duan C](http://www.ncbi.nlm.nih.gov/pubmed?term=Duan C%5BAuthor%5D&cauthor=true&cauthor_uid=18464606), [Tang J](http://www.ncbi.nlm.nih.gov/pubmed?term=Tang J%5BAuthor%5D&cauthor=true&cauthor_uid=18464606), [Feng :](http://www.ncbi.nlm.nih.gov/pubmed?term=Feng J%5BAuthor%5D&cauthor=true&cauthor_uid=18464606). **Cloning and expression of a beta-glucosidase gene umcel3G from metagenome of buffalo rumen and characterization of the translated product.**  *[Sheng Wu Gong Cheng Xue Bao](http://www.ncbi.nlm.nih.gov/pubmed/18464606#%23)* 2008, **24:**232-238. |
| Uncultured microorganisms from rabbit cecum metagenome | Unknown and uncultured microorganism | Spec. act.: 0.324 μmolmin^-1^μmol ^-1^ (pNPβGlu)  Spec. act.: 0.0011 μmolmin^-1^μmol ^-1^ (pNPβCel)  Spec. act.: 0.00257 μmolmin^-1^μmol ^-1^ (cellobiose)  Spec. act.: 9.41×10^-4^  μmolmin^-1^μmol ^-1^ (cellotriose)  Spec. act.: 9.90×10^-5^  μmolmin^-1^μmol ^-1^ (cellotetraose)  No activity for cellopentaose and cellohexaose  Opt. pH: 6.0 (90% activity at pH 5.0-9.0)  Opt Temp.: 40ºC (90% activity <30ºC and had low stability above 30ºC) | [Feng Y](http://www.ncbi.nlm.nih.gov/pubmed?term=Feng Y%5BAuthor%5D&cauthor=true&cauthor_uid=19584532), [Duan CJ](http://www.ncbi.nlm.nih.gov/pubmed?term=Duan CJ%5BAuthor%5D&cauthor=true&cauthor_uid=19584532), [Liu L](http://www.ncbi.nlm.nih.gov/pubmed?term=Liu L%5BAuthor%5D&cauthor=true&cauthor_uid=19584532), [Tang JL](http://www.ncbi.nlm.nih.gov/pubmed?term=Tang JL%5BAuthor%5D&cauthor=true&cauthor_uid=19584532), [Feng JX](http://www.ncbi.nlm.nih.gov/pubmed?term=Feng JX%5BAuthor%5D&cauthor=true&cauthor_uid=19584532): **Properties of a metagenome-derived beta-glucosidase from the contents of rabbit cecum**. *[Biosci Biotechnol Biochem](http://www.ncbi.nlm.nih.gov/pubmed/19584532?dopt=Abstract#%23)* 2009, **73:**1470-1473. |
| Uncultured microorganisms from faecal samples from earthworm species, *Aporrectodea caliginosa* and *Lumbricus terrestris* | Unknown and uncultured microorganism | G05-26  K_m_: 12.25 mM (pNPβGlu)  *k*_cat_: 360.8 s^-1^ (pNPβGlu)  *k*_cat_/Km: 2.9 × 10^4^ s^-1^M^-1^ (pNPβGlu)  K_m_: 14.60 mM (pNPβCel)  *k*_cat_: 43.4 s^-1^ (pNPβCel)  *k*_cat_/Km: 2.9 × 10^3^ s^-1^M^-1^ (pNPβCel)  K_m_: 12.63 mM (cellobiose)  *k*_cat_: 24.4 s^-1^ (cellobiose)  *k*_cat_/K_m_: 1.9 × 10^3^ s^-1^M^-1^ (cellobiose)  Able to hydrolase from cellotriose to cellopentaose: K_m_ from 17.6 to 24.9 mM; *k*_cat_ from 1.8 to 12.6 s^-1^; and *k*_cat_/K_m_ from 72.2 to 720 s^-1^M^-1^  Opt. pH: 8.5-9.0  Opt Temp.: 30-70ºC  G05-27  K_m_: 28.40 mM (pNPβGlu)  *k*_cat_: 43.5 s^-1^ (pNPβGlu)  *k*_cat_/Km: 1.5 × 10^3^ s^-1^M^-1^ (pNPβGlu)  No activity for cellooligosaccharides  Opt. pH: 8.5-9.0  Opt Temp.: 30-70ºC  G06-24  K_m_: 0.37 mM (pNPβGlu)  *k*_cat_: 204.9 s^-1^ (pNPβGlu)  *k*_cat_/Km: 5.5 × 10^5^ s^-1^M^-1^ (pNPβGlu)  No activity for cellooligosaccharides  Opt. pH: 5.5-9.0  Opt Temp.: 50ºC  G07-33  K_m_: 27.75 mM (pNPβGlu)  *k*_cat_: 23.4 s^-1^ (pNPβGlu)  *k*_cat_/Km: 0.8 × 10^3^ s^-1^M^-1^ (pNPβGlu)  No activity for cellooligosaccharides  Opt. pH: 8.0-9.0  Opt Temp.: 40-50ºC  G08-17  K_m_: 16.84 mM (pNPβGlu)  *k*_cat_: 20.0 s^-1^ (pNPβGlu)  *k*_cat_/Km: 1.2 × 10^3^ s^-1^M^-1^ (pNPβGlu)  K_m_: 6.64 mM (pNPβCel)  *k*_cat_: 119.0 s^-1^ (pNPβCel)  *k*_cat_/Km: 1.8 × 10^4^ s^-1^M^-1^ (pNPβCel)  K_m_: 9.21 mM (cellobiose)  *k*_cat_: 59.6 s^-1^ (cellobiose)  *k*_cat_/Km: 6.5 × 10^3^ s^-1^M^-1^ (cellobiose)  Opt. pH: 8.0-9.0  Opt Temp.: 55ºC  G10-10  K_m_: 10.52 mM (pNPβGlu)  *k*_cat_: 41.0 s^-1^ (pNPβGlu)  *k*_cat_/Km: 3.9 × 10^3^ s^-1^M^-1^ (pNPβGlu)  K_m_: 17.61 mM (pNPβCel)  *k*_cat_: 18.2 s^-1^ (pNPβCel)  *k*_cat_/Km: 1.0 × 10^3^ s^-1^M^-1^ (pNPβCel)  K_m_: 16.75 mM (cellobiose)  *k*_cat_: 13.7 s^-1^ (cellobiose)  *k*_cat_/K_m_: 8.2 × 10^2^ s^-1^M^-1^ (cellobiose)  Able to hydrolase cellotetraose (K_m_: 12.92 mM; *k*_cat_: 10.0 s^-1^; *k*_cat_/K_m_: 770 s^-1^M^-1^) and cellopentaose K_m_: 29.87 mM; *k*_cat_: 5.7 s^-1^; *k*_cat_/K_m_: 190 s^-1^M^-1^), but not cellotriose  Opt. pH: 9.0  Opt Temp.: 35-55ºC | Beloqui A, Nechitaylo TY, López-Cortés N, Ghazi A, Guazzaroni ME, Polaina J, Strittmatter AW, Reva O, Waliczek A, Yakimov MM, Golyshina OV, Ferrer M, Golyshin PN: [**Diversity of glycosyl hydrolases from cellulose-depleting communities enriched from casts of two earthworm species**.](http://www.ncbi.nlm.nih.gov/pubmed/20622123) *Appl Environ Microbiol* 2010, **76:**5934-5946 |
| **COMMERCIAL PREPARATIONS** | | | |
| Sigma (Aspergillus niger) | Fungi | K_m_: 1.0 mM (pNPβGlu)  K_m_: 2.7 mM (cellobiose)  No activity reported for cellooligosaccharides longer than cellobiose  Opt. pH: -  Opt Temp.: 25ºC | [Chauve M](http://www.ncbi.nlm.nih.gov/pubmed?term=Chauve M%5BAuthor%5D&cauthor=true&cauthor_uid=20181208), [Mathis H](http://www.ncbi.nlm.nih.gov/pubmed?term=Mathis H%5BAuthor%5D&cauthor=true&cauthor_uid=20181208), [Huc D](http://www.ncbi.nlm.nih.gov/pubmed?term=Huc D%5BAuthor%5D&cauthor=true&cauthor_uid=20181208), [Casanave D](http://www.ncbi.nlm.nih.gov/pubmed?term=Casanave D%5BAuthor%5D&cauthor=true&cauthor_uid=20181208), [Monot F](http://www.ncbi.nlm.nih.gov/pubmed?term=Monot F%5BAuthor%5D&cauthor=true&cauthor_uid=20181208), [Lopes Ferreira N](http://www.ncbi.nlm.nih.gov/pubmed?term=Lopes Ferreira N%5BAuthor%5D&cauthor=true&cauthor_uid=20181208): **Comparative kinetic analysis of two fungal beta-glucosidases**. *[Biotechnol Biofuels](http://www.ncbi.nlm.nih.gov/pubmed/20181208#%23)* 2010, **3:**3; Himmel ME, Adney WS, Fox JW, Mitchell DJ, Baker JO: **Isolation and characterization of 2 forms of beta-d-glucosidase from *Aspergillus niger***. *Appl Biochem Biotechnol* 1993, **39:**213–225. |
| Novozymes S188 (*Aspergillus niger*) | Fungi | K_m_: 1.03 mM (pNPβGlu)  K_m_: 5.63 mM (cellobiose)  V_max_: 3.76 μmolmin^-1^mg^-1^ (pNPβGlu)  V_max_: 33.74 μmolmin^-1^mg^-1^ (cellobiose)  No activity reported for cellooligosaccharides longer than ellobiose  Opt. pH: 4.5 (stable 4.0-4.5)  Opt Temp.: 60-70ºC (stable from 0-60º; t_1/2_ of >48 h at 50ºC) | [Chauve M](http://www.ncbi.nlm.nih.gov/pubmed?term=Chauve M%5BAuthor%5D&cauthor=true&cauthor_uid=20181208), [Mathis H](http://www.ncbi.nlm.nih.gov/pubmed?term=Mathis H%5BAuthor%5D&cauthor=true&cauthor_uid=20181208), [Huc D](http://www.ncbi.nlm.nih.gov/pubmed?term=Huc D%5BAuthor%5D&cauthor=true&cauthor_uid=20181208), [Casanave D](http://www.ncbi.nlm.nih.gov/pubmed?term=Casanave D%5BAuthor%5D&cauthor=true&cauthor_uid=20181208), [Monot F](http://www.ncbi.nlm.nih.gov/pubmed?term=Monot F%5BAuthor%5D&cauthor=true&cauthor_uid=20181208), [Lopes Ferreira N](http://www.ncbi.nlm.nih.gov/pubmed?term=Lopes Ferreira N%5BAuthor%5D&cauthor=true&cauthor_uid=20181208): **Comparative kinetic analysis of two fungal beta-glucosidases**. *[Biotechnol Biofuels](http://www.ncbi.nlm.nih.gov/pubmed/20181208#%23)* 2010, **3:**3; Dekker RFH: **Kinetic, inhibition, and stability properties of a commercial beta-D-glucosidase (cellobiase) preparation from aspergillus-niger and its suitability in the hydrolysis of lignocellulose**. *Biotechnold Bioeng* 1986, 28:1438–1442. |
| Novozym 188 (produced in *Aspergillus* *niger*) | Fungi | K_m_: 0.45 mM (pNPβGlu)  V_max_: - μmolmin^-1^mg^-1^ (pNPβGlu)  K_m_: 0.35 mM (cellobiose)  V_max_: - μmolmin^-1^mg^-1^ (cellobiose)  No activity reported for cellooligosaccharides longer than cellobiose  Opt. pH: 4.0-5.0; 20% residual activity outside these pH values  Opt Temp.: 50ºC | [Krogh KB](http://www.ncbi.nlm.nih.gov/pubmed?term=Krogh KB%5BAuthor%5D&cauthor=true&cauthor_uid=19756584), [Harris PV](http://www.ncbi.nlm.nih.gov/pubmed?term=Harris PV%5BAuthor%5D&cauthor=true&cauthor_uid=19756584), [Olsen CL](http://www.ncbi.nlm.nih.gov/pubmed?term=Olsen CL%5BAuthor%5D&cauthor=true&cauthor_uid=19756584), [Johansen KS](http://www.ncbi.nlm.nih.gov/pubmed?term=Johansen KS%5BAuthor%5D&cauthor=true&cauthor_uid=19756584), [Hojer-Pedersen J](http://www.ncbi.nlm.nih.gov/pubmed?term=Hojer-Pedersen J%5BAuthor%5D&cauthor=true&cauthor_uid=19756584), [Borjesson J](http://www.ncbi.nlm.nih.gov/pubmed?term=Borjesson J%5BAuthor%5D&cauthor=true&cauthor_uid=19756584), [Olsson L](http://www.ncbi.nlm.nih.gov/pubmed?term=Olsson L%5BAuthor%5D&cauthor=true&cauthor_uid=19756584): **Characterization and kinetic analysis of a thermostable GH3 beta-glucosidase from *Penicillium brasilianum*.** *[Appl Microbiol Biotechnol](http://www.ncbi.nlm.nih.gov/pubmed/19756584#%23)* 2010, 86:143-154. |
| NS50010 | Fungi | Spec. act.: 1.3 μmolmin^-1^mg ^-1^ (pNPβGlu; 0.5 g/l) at 50ºC | Commercial β-glucosidase (Novozymes AS) |
| NS50031 | Fungi | Spec. act.: 74.6 μmolmin^-1^ml ^-1^ (pNPβGlu)  Spec. act.: 601 μmolmin^-1^ml ^-1^ (cellobiose) | Commercial cellulase (Novozymes AS) |
| NS50073 | Fungi | Spec. act.: 177.3 μmolmin^-1^ml ^-1^ (pNPβGlu)  Spec. act.: 1691 μmolmin^-1^ml ^-1^ (cellobiose) | Commercial cellulase (Novozymes AS) |
| E-BGOSAG (*Agrobacterium* sp.) |  | Spec. act. (as reported): 211 μmolmin^-1^mg ^-1^ (pNPβGlu)  Spec. act. (as reported): 233 μmolmin^-1^mg ^-1^ (cellobiose)  Spec. act. measured (in-house) at 50ºC and pH 5.0: 109000 μmolmin^-1^mg ^-1^ (pNPβGlu; 0.1 g/l))  Spec. act. measured (in-house) at 50ºC and pH 5.0: 119 μmolmin^-1^mg ^-1^ (cellobiose; 0.1 g/l))  No activity reported for cellooligosaccharides longer than ellobiose  Opt. pH: 6.5-7.0 (at 40ºC)  Opt Temp.: 50ºC (10 min, pH 7.0) (unstable above 50ºC) | Commercial β-glucosidase (Megazyme) |
| G0395 (almond) |  | Spec. act. measured (in-house) at 50ºC and pH 5.0: 42 μmolmin^-1^mg ^-1^ (pNPβGlu)  Spec. act.: 2.3 μmolmin^-1^mg ^-1^ (lyophilized powder) (cellobiose)  Resuspended in water, the solution shows a low activity at 50ºC and pH 5.0 (0.29 units mg^-1^ powder or 42 mUmg^-1^ protein)  The enzyme was shown to be non thermostable | Commercial β-glucosidase (Sigma Chemical Co.) |

Abbreviations as follows: *p*NPαGlu: *p*-nitrophenyl-α-D-glucoside; *p*NPβCel: *p*-nitrophenyl-β-D-cellobioside.

^1^One unit (U) of enzyme activity is defined as the amount of enzyme producing 1 μmol of reaction products in 1 min under the assay conditions
