# Supplementary figures and images for: Microbial β-glucosidases from cow rumen metagenome enhance the saccharification of lignocellulose in combination with commercial cellulase cocktail
Source: Biotechnol Biofuels. 2012 Sep 21;5:73. doi: 10.1186/1754-6834-5-73 (PMC3477023; doi:10.1186/1754-6834-5-73)

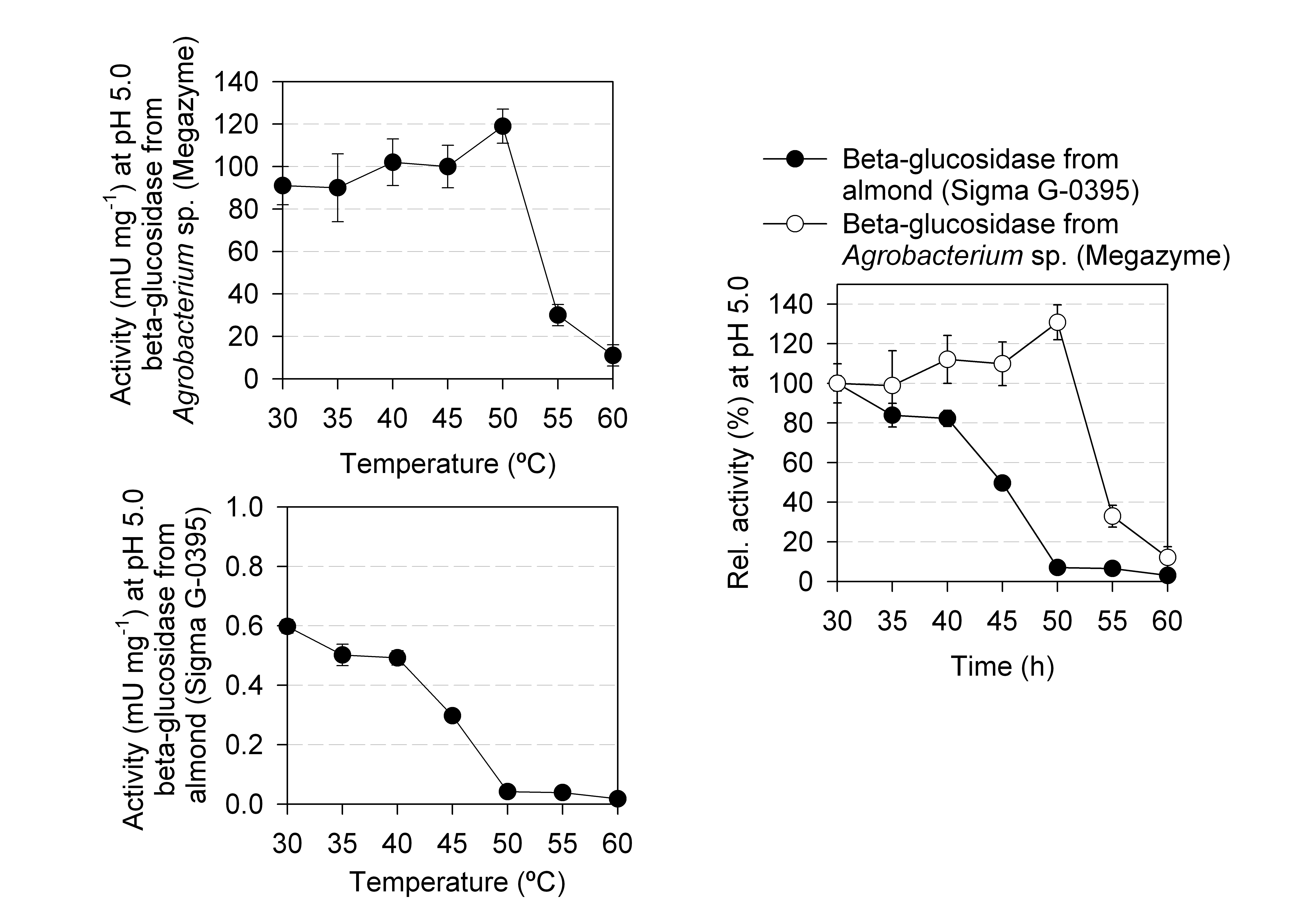

Supplement: Additional file 3 — Figure S1.Temperature optima of commercial β-glucosidases from almond (G0395; Sigma Chemical Co.) and Agrobacterium sp. (E-BGOSAG; Megazyme). The parameters were determined using pNPβG (0.1 mg ml-1) as the substrate at pH 5.0 (sodium acetate). All measurements were analysed in triplicate and error bars are indicated. Left panel shows the specific activity and right panel the relative activity referred to that at 30°C. [file 1754-6834-5-73-S3.jpeg]
